# Social determinants of multimorbidity patterns: A systematic review

**DOI:** 10.3389/fpubh.2023.1081518

**Published:** 2023-03-27

**Authors:** Javier Álvarez-Gálvez, Esther Ortega-Martín, Jesús Carretero-Bravo, Celia Pérez-Muñoz, Víctor Suárez-Lledó, Begoña Ramos-Fiol

**Affiliations:** ^1^Department of Biomedicine, Biotechnology and Public Health, University of Cadiz, Cádiz, Spain; ^2^The University Research Institute for Sustainable Social Development (Instituto Universitario de Investigación para el Desarrollo Social Sostenible), University of Cadiz, Jerez de la Frontera, Spain; ^3^Department of Nursing and Physiotherapy, University of Cadiz, Cádiz, Spain

**Keywords:** multimorbidity, comorbidity, chronic conditions, chronicity, social determinants, behavioral determinants

## Abstract

Social determinants of multimorbidity are poorly understood in clinical practice. This review aims to characterize the different multimorbidity patterns described in the literature while identifying the social and behavioral determinants that may affect their emergence and subsequent evolution. We searched PubMed, Embase, Scopus, Web of Science, Ovid MEDLINE, CINAHL Complete, PsycINFO and Google Scholar. In total, 97 studies were chosen from the 48,044 identified. Cardiometabolic, musculoskeletal, mental, and respiratory patterns were the most prevalent. Cardiometabolic multimorbidity profiles were common among men with low socioeconomic status, while musculoskeletal, mental and complex patterns were found to be more prevalent among women. Alcohol consumption and smoking increased the risk of multimorbidity, especially in men. While the association of multimorbidity with lower socioeconomic status is evident, patterns of mild multimorbidity, mental and respiratory related to middle and high socioeconomic status are also observed. The findings of the present review point to the need for further studies addressing the impact of multimorbidity and its social determinants in population groups where this problem remains invisible (e.g., women, children, adolescents and young adults, ethnic groups, disabled population, older people living alone and/or with few social relations), as well as further work with more heterogeneous samples (i.e., not only focusing on older people) and using more robust methodologies for better classification and subsequent understanding of multimorbidity patterns. Besides, more studies focusing on the social determinants of multimorbidity and its inequalities are urgently needed in low- and middle-income countries, where this problem is currently understudied.

## 1. Introduction

Multimorbidity can be defined as the co-occurrence of two or more chronic diseases or long-term medical conditions in an individual (1–4). This condition is associated with increased disability and functional impairment, lower quality of life, increased health services utilization, fragmentation of care, polypharmacy, complex treatment, and increased mortality (5–7), particularly among older people. The progressive increase in the number of patients with multimorbidity represents a major global challenge for daily clinical practice, as well as for health systems, governments, and epidemiological research (8–11). Recent evidence has highlighted the limitations of current healthcare systems for addressing the complex needs of patients with comorbidity and multimorbidity due to inadequate or absent attention to coexisting chronic conditions (12–15).

The prevalence of chronic conditions among older populations, such as obesity, hypertension, diabetes, heart disease, chronic obstructive pulmonary disease, musculoskeletal disease, mental disorders, or cancer is growing due to the progressive increase in life expectancy. However, the increase in multimorbidity is only partially explained by population aging (11, 16). Multimorbidity also affects the young population in Western countries, particularly those living in low- and middle-income countries (LMIC) (17). According to the World Health Survey, the average prevalence of multimorbidity is 7.8% in 28 LMICs (11). Similarly, the WHO Study on global AGEing and adult health (SAGE) indicates that more than one-fifth of participants in six LMICs had multimorbidity (18). Recent studies show that social determinants play an essential role in these differences, showing an advance of 10–15 years in the age of onset of multimorbidity among populations with fewer socioeconomic resources (19–21).

Social determinants shape the distribution of health inequalities and chronicity (22). In the same way as communicable diseases, non-communicable diseases are also determined by social and economic differences between populations (socioeconomic status, educational level, economic hardship, lifestyles, and risk behaviors, among others) (23–25). Although the impact of social inequalities on health is clearly identified in the specialized literature, the pattern of association of the different chronic conditions that make up multimorbidity and their respective social and behavioral determinants are not so well-defined (1, 15, 26–29), even though these are the factors that social and health policies could most easily address. Recent work shows that multimorbidity is strongly associated with social and economic factors that condition the early onset of chronicity and subsequent multimorbidity in disadvantaged social groups (1, 19). Indeed, those of low socioeconomic status experience the most negative effects of multimorbidity (30). However, to date, there is little evidence that provides us with information on other types of social and behavioral determinants (such as ethnic characteristics, lifestyles, income, living area, among others) that could be fundamental in the emergence and evolution of multimorbidity patterns that are less prevalent than cardiovascular and musculoskeletal ones, which are the most studied in the literature (for example, multimorbidity among younger groups, mental health patterns, and combined or complex patterns). Therefore, further research is needed to improve our current knowledge of multimorbidity patterns and the social and behavioral determinants that may characterize the different multimorbidity profiles, which is a fundamental step for policy planning and the future sustainability of health systems (31–33).

Current clinical guidelines do not cover all the needs of patients with multimorbidity due to health care that is not tailored to the needs of people with multimorbidity (10). In fact, understanding the different pathological processes of chronicity could improve treatment by focusing on the disease cluster rather than treating individual diseases separately (34). Since there is consistent evidence that chronic diseases tend to accumulate according to certain patterns (15, 19), the identification of specific multimorbidity classes and the social and behavioral factors that give rise to them could help healthcare providers to predict the probability of the occurrence of co-joint chronic conditions, and thus be able to control the appearance of future comorbidities while improving patients' quality of life (15, 35, 36).

Some of these early studies have addressed the social determinants of certain multimorbidity patterns (35). The multimorbidity profiles described in these studies range from well-known chronic disease combinations that conform the most prevalent patterns (e.g., cardiovascular, musculoskeletal, or mental disorders) to more complex disease conglomerates that are usually appear in older age groups (15, 19). The great diversity in the occurrence of these groups of conditions leads to a lack of studies that provide a complete description of the wide range of different multimorbidity patterns (i.e., understood in this context as the specific combination of co-joint chronic conditions) identified through different classification methods, nor do we find studies that comprehensively address the association with the social and behavioral determinants that could affect the different chronicity profiles (34, 38). While some studies have been commonly focused on determinants such as gender and/or age, other studies have been particularly oriented toward the global indicators of multimorbidity (i.e., commonly counting the number of chronic conditions) without taking into account the specific aggregation pattern of chronic diseases (15, 19, 37–39). In this regard, to the best of our knowledge, to date there is no study that provides a comprehensive characterization of the social and behavioral determinants of multimorbidity patterns that have been discovered using diverse methods.

The present systematic review aims to fill this knowledge gap. Specifically, with this work, we aim to (1) characterize the different multimorbidity patterns described in specialized literature; (2) identify the social and behavioral determinants of these multimorbidity profiles (or classes); and (3) describe and compare the different methods used for the classification of multimorbidity profiles, their advantages, and possible limitations. Thus, studies analyzing multimorbidity patterns related to social and behavioral determinants were systematically reviewed.

## 2. Materials and methods

This systematic review was registered on 10 May 2022 in the PROSPERO database (CRD4202232328140). This systematic review was conducted according to the Preferred Reporting Items for Systematic Reviews and Meta-Analyses (PRISMA) guidelines (40).

### 2.1. Inclusion and exclusion criteria

Our inclusion criteria were as follow:

- Studies with defined patterns of physical and/or mental multimorbidity. Following the World Health Organization definition, we used a broad concept of multimorbidity as the coexistence of two or more chronic conditions in the same individual.
- In addition, we selected studies that associate multimorbidity patterns to social determinants. Specifically, we considered sociodemographic (age, gender, marital status), socioeconomic (education, income, working status, among other) and behavioral (lifestyles) determinants.
- We only reviewed studies written in English and Spanish.
- Finally, we selected articles published from January 1, 2011 to December 31, 2021. We consider this 10-year time frame broad enough to ensure comparability of studies. In fact, although there are earlier studies on multimorbidity it is not until 2012–2014 that the literature begins to focus specifically on multimorbidity patterns (15).

Our exclusion criteria were:

- Studies that were not research articles published in scientific journals (i.e., abstracts, doctoral theses, editorials, press articles, commentaries, journal letters, books and all types of reviews).
- We excluded studies with multimorbidity patterns with a specific underlying disease (i.e., patterns in people with diabetes, with dementia, or similar).
- Moreover, multimorbidity studies with communicable and non-chronic diseases were excluded.

### 2.2. Search strategy

The search was performed by B.R.-F in the databases PubMed/MedLine, Embase, Ovid MEDLINE, PsycINFO, Web of Science, Scopus, and CINAHL Complete on January, 2022. Google Scholar was used to supplement the main databases. This search was repeated in July 2022. The search strategy for this review was performed by combining three sets of keywords (multimorbidity, social-behavioral determinants, and patterns) to search for relevant literature using Boolean operators. The search strategy, which can be found in Supplementary Table 1, was adapted to each database. The references obtained from the search strategy were exported to Microsoft Excel 2019 for deduplication of the results and screening.

### 2.3. Quality assessment

The quality of the studies was evaluated using Axis Tool quality assessment (41), a critical appraisal tool to systematically assess the methodological quality of selected studies. This tool consists of 20 questions with expanded explanations that are assessed with yes/no/don't know (for more information see the Supplementary Table 2). These questions evaluate general aspects of the research and reporting, such as the introduction, methodology, and quality of the discussion. The purpose of these questions is to guarantee the quality of the selected studies. The tool was implemented by two reviewers (BR-F, EO-M) that, in the case of disagreement, solved the possible discrepancies using the scores of a third reviewer. The three reviewers jointly analyzed the articles with the lowest methodological score (in our case, two articles with a score of 16/20). After a full evaluation, these articles were retained by the agreement of the reviewers.

### 2.4. Data extraction and analysis

First, titles and abstracts were examined by four reviewers (JC-B, EO-M, CP-M, BR-F). Subsequently, the full texts of the retrieved articles were reviewed according to the eligibility criteria. For data extraction from the selected articles, tables created for their organization were used. The information extracted from each publication was: author, title, year of publication, abstract, objective, conclusions, method of pattern extraction, multimorbidity patterns, social-behavioral factors, and population characteristics. Due to the breadth and variety of results the data were divided into different tables. The first table contained the identifying data of the articles (title, authors, and year) (Supplementary Table 3) as well as the summarized content of the article (objectives, topics, and conclusions). The second table compiled the pattern extraction methods and the results obtained, such as multimorbidity patterns, associated social-behavioral factors and the characteristics of the population studied (Supplementary Table 4). The third table combined the articles presenting each social-behavioral determinant according to the pattern extraction method (Supplementary Table 5). For the data analysis, a narrative synthesis and summary of the different methods of identifying patterns and their association with different social determinants were described in order the identify the work done so far and knowledge gaps in this field.

## 3. Results

**Figure 1:**
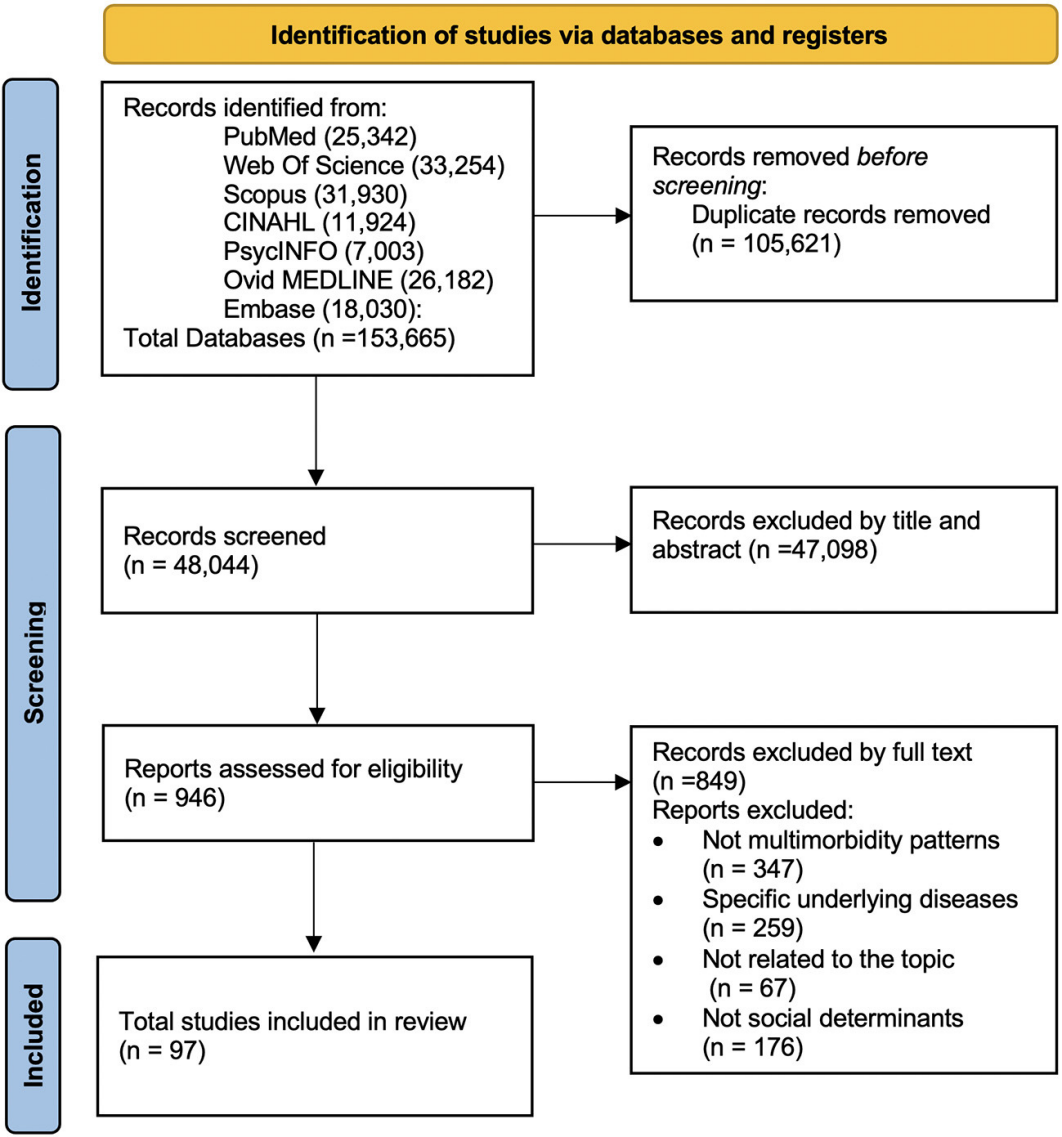
PRISMA flow diagram (40).

A total of 135,665 articles were identified, of which 48,044 remained after removing duplicates. After screening titles and abstracts, 946 articles were examined for full text. Finally, a total of 97 articles were selected for review after full-text reading and review of methodological quality (Figure 1).

Among the selected studies, five main groups of classification techniques for the identification of multimorbidity patterns were found: (1) latent class analysis (42–78); (2) cluster analysis techniques (79–100); (3) factor analysis (101–119); (4) machine learning methods (120–129); and (5) based on expert knowledge (130–137), from the most to the least frequent. The cluster technique included studies using k-means, fuzzy c-means and hierarchical clustering. Factor analysis comprised exploratory factor analysis and principal component analysis. Finally, machine learning tools comprised methods based on network techniques, self-organizing maps, and non-negative matrix factorization.

In total, 41 social and behavioral determinants were found, which were grouped and subsequently analyzed around six principal domains: (1) sociodemographics (including sex, age, and marital status); (2) socioeconomic status (education, poverty, deprivation index, income management, household wealth, occupation, socioeconomic level, occupational status, index of relative socioeconomic disadvantage, financial wealth, skin color/race, and migrate status); (3) lifestyle (smoke, physical activity, drink, sleep quality, substance use, body mass index (BMI), lifestyle risk factor, diet and waist circumference); (4) social networks and social relationships (degree of social relationships, size of social network, and loneliness); (5) living area characteristics (residence place, violence area, area deprivation, area level-income, area level education, area infrastructure); and (6) health service use (type of health insurance, visits, etc.). Table 1 summarizes the main social determinants associated with multimorbidity according to the identified methods for multimorbidity pattern extraction. As we can observe, sociodemographic, socioeconomic and behavioral determinants have been the factors more commonly studied through methods such as latent class analysis, cluster and factor analysis techniques and, recently, through machine learning tools.

**Table 1:** Social determinants by multimorbidity pattern extraction method.

**Social determinants**	**Latent class analysis (*n* = 37)**	**Cluster techniques (*n* = 22)**	**Factor analysis (*n* = 19)**	**Machine learning (*n* = 10)**	**Expert knowledge (*n* = 8)**
Sociodemographic	34	21	17	9	8
Socioeconomic status	34	8	10	2	2
Lifestyle and behavioral	20	6	2	0	2
Living area characteristics	7	2	3	0	1
Health service use	9	2	1	2	0
Social networks and relationships	3	2	1	0	1

### 3.1. Sociodemographic characteristics

A broad set studies detected a stronger association of mental and musculoskeletal multimorbidity with female sex, including neurological diseases (64, 130), musculoskeletal and osteoarticular (42, 64, 67, 71, 93, 102, 126, 130, 131); and common mental disorders (42, 132), like anxiety (44, 67, 86, 93, 114) depression (43, 67, 86, 93, 114, 119), dementia (85) and emotional disorders (47). While cardiometabolic patterns (53, 71, 73, 78, 82, 90, 94, 114, 123), musculoskeletal (53, 96), digestive and genitourinary patterns (99, 110, 124, 131) were more frequent in men. As expected, a positive association between multimorbidity and age was found (67, 74, 105, 110, 132, 133). Although it was also possible to find multimorbidity among the young population, this group was usually classified in patterns called “relatively healthy” (44, 50, 60, 70, 78, 90, 125). The more prevalent multimorbidity patterns in older population were: cardiovascular (44, 58, 60, 71, 72, 81, 86), cardiometabolic (61, 73, 76, 94), musculoskeletal (81, 86, 94), cognitive impairment or dementia-related (60, 86), arthritis-cataracts (53, 58, 72, 87) and respiratory-mental-articular (56, 61). Mental health patterns linked to anxiety and depression were more frequent in younger people (44, 47, 50, 58, 86). However, multimorbidity patterns linked to dementia and cognitive impairment increase significantly with age (111, 128).

In several studies, being married was a protective factor against multimorbidity. In fact, those married were commonly classified in the relatively healthy class (55, 60, 66, 70, 72, 78, 97), while being divorced, separated, or cohabiting couples were more likely to be linked to mental health multimorbidity patterns such as the denominated “depression cluster” (66) and divorced people were more likely to be in the “psychiatric class” (97). In contrast, single people were more likely to belong to the complex multimorbidity pattern (42, 59). Widowers were more likely to belong to dementia, geriatric or neurodegenerative disease classes (78, 128), depression (66) and cardiorespiratory or musculoskeletal patterns (56).

### 3.2. Socioeconomic status

Positive associations were found between household income and multimorbidity (107, 133). In particular, people with lower income presented a higher risk of being in the depression-arthritis (58), depression-chronic pain (48), respiratory (107), and mental health patterns (138). Overall, they were relatively healthy (52, 74) even though having multimorbidity. Conversely, having a low income was associated with a higher probability of belonging to the cardiometabolic pattern (53, 60, 65, 76), suffering musculoskeletal disease (sometimes combined with mental health conditions) (53, 107), and respiratory patterns (53, 76). The proportion of patients with multimorbidity who had physical-mental comorbidities increased substantially with greater socioeconomic deprivation (132, 138). In particular, high-income groups were less likely to present comorbidities such as depression-arthritis (58), depression-chronic pain (48), rhinitis-allergies (107), heart-related diseases or belonging to severe (or complex) multimorbidity classes (44). In general, the higher their income, the more likely they were to belong to the “relatively healthy” class (52, 74). Conversely, having a low income was found to be associated with a higher likelihood of belonging to the cardiometabolic pattern (60), depression-insomnia and musculoskeletal-mental-functional (107), cardiopulmonary and cerebrovascular (65), arthritis, asthma, allergy, depression, and thyroid-related diseases (76), cardiorespiratory-arthritis-cataracts and metabolic classes (63). They were also associated with classes linked to alcohol and substance abuse problems (43). In general, having a low income increased the likelihood of belonging to the classes with the highest rate of disease and mortality (44, 48, 49). In parallel, chronic disease in adulthood tended to increase the risk of poverty (104). Material deprivation was a risk factor for all multimorbidity patterns (59).

Different studies show that low educational levels increase the risk of suffering multimorbidity (42, 70, 75). Low education is associated with cardiometabolic (42, 60), musculoskeletal (75), respiratory (42) and mental health multimorbidity patterns (58), which are commonly organized around depressive disorders. On the other hand, high education was found to be a protective factor among the severely impaired population, metabolic and joint-COPD-ulcer patterns in the study by Zacarías-Pons et al. (59). However, a high educational level also presented a positive association with mental health patterns (specifically, psychiatric disorders and depression) (58). Similarly, being a woman with good education and high income was mainly related to the anxiety-related classes (44). Patterns were also found to be associated with low socioeconomic status (i.e., low education level and low income), such as cardiometabolic (76, 104), metabolic (74), or respiratory-mental-musculoskeletal ones (103). Low educational level and difficulty managing income were also significantly associated with a higher likelihood of having psychosomatic and musculoskeletal patterns (108).

Concerning occupational status, never working increased the risk of belonging to the cardiometabolic class (108) and being unemployed of being classified in multimorbidity patterns linked to substance use (i.e., alcohol, tobacco, and other drugs) (77). Similarly, non-working (42), being unemployed, or pre-retired (70) increased the odds of belonging to any multimorbidity class except the respiratory ones (i.e., characterized by asthma-allergy conditions). Civil servants presented a higher prevalence of musculoskeletal patterns (75) similar to manual workers, which even had a higher probability of belonging to the cognitive and sensory impairment-related classes (97). Also, the vascular-inflammatory class was associated with a low educational level and unemployment (74).

In terms of ethnicity, most studies assessed were focused on Caucasian population (44, 47, 48, 52, 60, 86, 123, 128). Race or ethnicity was found to be associated with certain chronic diseases. Van Cleave et al. (65) found a higher prevalence of cardiopulmonary and cerebrovascular patterns among black men, while Bisquera et al. (86) found that pain and liver disease conditions were also most probably linked with this population group. On the other hand, Janssen et al. (128) found associations with liver patterns and other physical injuries among Native Americans. Kalgotra et al. (123) found associations between endocrine, nutritional, immune, injury, and poisoning-related diseases. The Asian population was linked to cerebrovascular patterns and gastrointestinal cancer (128), and the Caucasian population was commonly associated with mental and musculoskeletal patterns (123).

Regarding migration status, Diaz et al. (109) found that the patterns among Norwegians and immigrants from Western Europe and North America were similar. However, multimorbidity patterns were not the same when compared with other immigrant groups. Janssen et al. (128) found that cardiovascular, cancer and geriatric patterns were more prevalent among local US residents, while urological, gastrointestinal, or respiratory disease patterns were more frequent among non-residents.

### 3.3. Lifestyle and behavioral factors

Risk behaviors such as alcohol consumption or smoking increased the risk of belonging to all multimorbidity classes, especially in men (54, 79, 108), and also in complex mental health patterns in young men (138). However, several articles report that smoking is also a risk factor for the cardiometabolic class (60, 73), which is associated with other determinants such as less education, lower income and related overweight (60) or higher waist circumference, physical activity, and a history of asthma or heart attack (73). In the study by Barile et al. (68), the mental health class was the most likely to smoke currently. In fact, 46% of people aged 18–44 in the depression class were regular smokers (43). Smoking was a risk factor only for the respiratory-mental-articular class (61). Other studies found that smoking increased the risk for the musculoskeletal class (75, 108). And, as expected, being a current or former smoker also increases the risk of belonging to respiratory multimorbidity patterns (108). Simoes et al. (67) found a direct association between smoking and obesity in the respiratory class. In the cardiorespiratory-arthritis-cataract pattern, the probability of membership increased with increasing age and being a smoker (53).

The relationship between alcohol consumption and multimorbidity was mediated by the age groups and related chronic conditions. Hernández et al. (49) found variations by the country for the relationship between alcohol and multimorbidity patterns. In the USA, the association was negative for all patterns. In contrast, in Canada and England, it was harmful to the patterns of high disease probability and metabolic and arthritis. Finally, positive associations were found in certain patterns such as osteoporosis, arthritis, hypertension, and metabolic in Ireland. According to the study by Jackson et al. (108), people who did not drink alcohol were more likely to belong to the cardiometabolic pattern. Specifically, 50% of people with complex cardiometabolic and cognitive impairment patterns were abstainers. This figure increased in the respiratory patterns where the number of moderate-strong drinkers was highest, while the younger heaviest drinking group were also the healthy subjects (60).

Physical activity was a protective factor for the respiratory-mental-articular class (61). However, physical activity significantly decreased in several groups having certain chronic conditions such as diabetes (75), respiratory and vascular-inflammatory (74). Likewise, some studies (58, 59) found more frequent intense physical activity among relatively healthy classes. On the contrary, lower physical activity was more characteristic in population groups with cardiovascular, musculoskeletal and mental health conditions (68, 97).

Although we did not find studies that analyzed the impact and/or relationship of diet with multimorbidity patterns, a clear association was observed between BMI and the subsequent occurrence of chronic comorbidities. For this reason, we used this indicator as a proxy variable for diet. In particular, obesity increases the risk of belonging to all multimorbidity classes (67, 75). Overweight and obesity were found to be risk factors for musculoskeletal or severe impairment (44) and cardiometabolic (108). Similarly, patterns of physical and mental conditions (68), complex metabolic, respiratory, and age-related conditions (60) were also associated with an increased risk of obesity. Depression was strongly associated with overweight or obesity among females (67).

### 3.4. Living area characteristics

Regarding the type of area of residence, we found that multimorbidity was more prevalent in urban areas (98). For instance, the cardiometabolic burden was lower in rural areas. Territorial characteristics such as the level of crime and violence in the area were also found to be associated with a pattern of chronic pain and respiratory diseases in men (101). In these areas of higher economic deprivation and lower socioeconomic status (i.e., lower household income and lower education levels), cardiometabolic, respiratory, mental and musculoskeletal patterns were more prevalent (104). Consequently, the possible effect of the area of residence over multimorbidity patterns was linked to specific socioeconomic conditions of the groups. In any case, the existing literature does not provide a clear characterization of either the social mechanisms or the ecological effects involved in the association between the local area of residence and multimorbidity patterns.

### 3.5. Health service use

Being insured at the medical level presented important variations depending on the different countries and the respective health systems, so no clear association pattern was detected (74, 125). For instance, in the US, people with Medicaid and an additional private insurance were more likely to be found in the oncology and neurological patterns (45). Regarding the use of health services, in the study by Craig et al. (74), all classes of multimorbidity were significantly associated with higher use of health services. Similarly, patterns associated with cardiometabolic complications in pregnancy, vascular and cancer were directly related to unplanned readmissions (121). More specifically, Buja et al. (57) found that patterns such as cardiac, metabolic-ischaemic, cardiorespiratory, or cancer impairment were more at risk of hospitalization than the neurological class. Patterns linked to substance use were also associated with having more than three emergency department visits (77). Egan et al. (79) associated more hospitalizations in congestive heart failure (35%), neurological (27%), and chronic kidney-related disease patterns (24%).

### 3.6. Social networks and relationships

In the study of 2020 by Marengoni et al. (97), the patterns of cognitive and sensory impairment had the highest percentage of people with a poor (or reduced) social network and inadequate physical activity levels. In addition, a large network of friends and having social support were found to reduce the risk of presenting patterns of degenerative-digestive diseases while increasing adherence to medications (62). Moreover, in the same way that social support has been identified as a protective factor against chronicity, the suicide of a family member has also been identified as a risk factor in the development and consolidation of severe patterns of mental multimorbidity (138). On the other hand, the cardiometabolic and respiratory-mental-articular patterns between the ages of 50 and 64 years were associated with a greater presence of feelings of loneliness, limitations in activities of daily living, and a worse state of health (61). On the other hand, the probability of suffering from degenerative-digestive diseases seemed to increase in patients with poor sleep quality and an unbalanced diet, despite having a large family network and a medium-high income (62).

Poor quality of life was associated with belonging to the complex cardiometabolic and respiratory classes (42), and activities of daily living were also affected to a greater extent by specific patterns such as cardiovascular, respiratory, mental and articular (56, 61).

**Figure 2:**
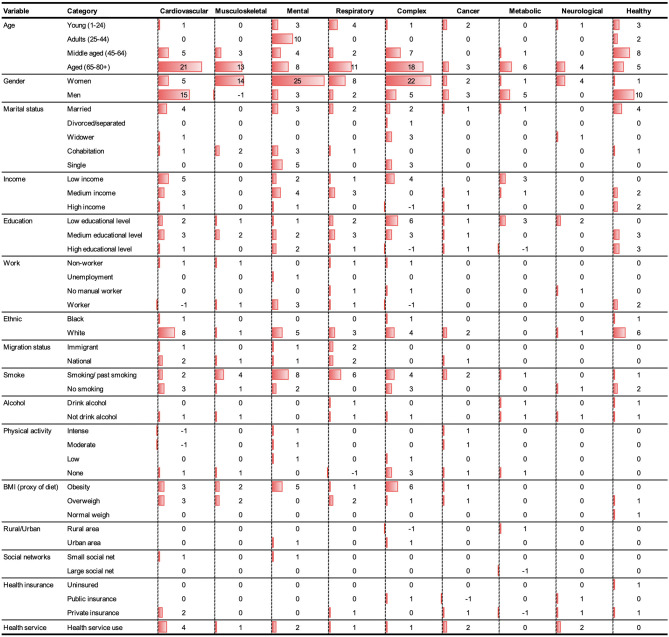
Number of associations identified between different social determinants and the main multimorbidity patterns.

With the intention of reducing the diversity of multimorbidity patterns that we have found in the different studies analyzed up to this point, the most prevalent and relevant profiles were synthesize around nine global classes (in decreasing order): (1) Cardiovascular; (2) Musculoskeletal; (3) Mental; (4) Respiratory; (5) Complex (characterized by many simultaneous chronic diseases or complicated multimorbidity); (6) Cancer; (7) Metabolic; (8) Neurological; and (9) Relatively Healthy (i.e., those who present minor or less serious chronic conditions). Figure 2 describes the number of relationships identified between the most frequent patterns and the respective social determinants (negative values denote that an inverse statistical association was identified).

## 4. Discussion

To the best of our knowledge, this is the first systematic review to perform a comprehensive study of the association between multimorbidity and its social and behavioral determinants while considering the statistical methods employed to extract multimorbidity patterns. However, it is possible to find other review papers that separately address multimorbidity patterns (17, 35, 139, 140) or their association (141–143) with specific patterns. Until now, no work has integrated the wide diversity of multimorbidity patterns, social determinants and classification methods. The results yielded 97 publications from which 41 different social determinants (i.e., including sociodemographics, socioeconomic and individual behavioral ones) and the nine most prevalent multimorbidity patterns described in the literature were synthesized. The articles were very diverse regarding extraction methods, determinants studied and the resulting multimorbidity patterns. Nevertheless, some interesting findings were identified.

Although in the present study, we have considered the “relatively healthy” class to characterize individuals with less severe conditions of this problem (e.g., allergies related), the most commonly observed patterns have been the following: cardiovascular, musculoskeletal, mental; respiratory, complex, cancer, metabolic, and neurological. Among these global multimorbidity domains, the three most prevalent patterns identified were generally characterized by musculoskeletal, mental and cardiovascular conditions (17). However, recent studies have identified a fourth highly prevalent pattern characterized by the presence of different allergy related diseases (i.e., seasonal allergies, asthma, digestive or skin problems) (144–146), which in our search is mainly diluted in the respiratory (20, 73, 101, 108) and complex (44, 69) patterns.

From a sociodemographic point of view, our study reveals important differences in the multimorbidity patterns of men and women, which, according to the results described, seem to be linked to avoidable social inequalities that can be linked to the social and economic conditions of these groups. Thus, while musculoskeletal (42, 64, 67, 71, 93, 102, 126, 130, 131) and mental patterns (43, 67, 86, 93, 114, 119) are more typical of women, cardiovascular patterns are more prevalent in men (24, 53, 71, 73, 78, 82, 90, 94, 123), which leads us to believe that behind these results there are also important differences in the individual behaviors and lifestyles of these groups (e.g., considering the differences among married and individuals living alone) (55, 60, 66, 70, 72, 78, 97). Likewise, in the case of age, the present results show that multimorbidity is not only a matter of older people (although it is more visible in these age groups) (89, 104, 105, 137) since certain types of multimorbidity are also observed in young and middle age people, such as mental and allergy multimorbidity patterns (42, 95). In addition, we must consider that most of the studies were focused on the older population. Hence, multimorbidity patterns in children and young are clearly under-represented and under-studied (45, 46).

Similarly, with a few exceptions (51, 65), most studies did not consider ethnic or racial differences. In fact, most reviewed works were oriented to Western countries' populations and mainly applied to white Caucasians groups, even though specific multimorbidity patterns have been found to be more prevalent in African Americans (147). Therefore, multimorbidity patterns described in many of the studies and their association with specific social determinants may be biased in terms of race and ethnicity. Therefore, studies with a more heterogeneous population would be needed to provide a better characterization and measurement of this problem.

Most of the studies in this review demonstrate that a lower socioeconomic status commonly increases the risk of suffering severe multimorbidity (e.g., musculoskeletal, cardiovascular, complex patterns) (44, 48, 49, 53, 59, 60, 63, 65, 76, 107, 132, 133). Multimorbidity patterns can also be linked to social and living conditions and individual lifestyles. For instance, low educational attainment and living in a deprived area were associated with a higher risk of multimorbidity (40, 42, 58, 70, 74–76, 103, 104, 108). People with a low level of education may have a higher risk of health illiteracy and difficulties in finding, understanding and applying health care information (148). In addition, our results show that the behaviors and lifestyles associated with morbidity patterns are in parallel mediated by population socioeconomic conditions. The results show that alcohol and tobacco consumption can substantially impact the appearance of cardiometabolic, musculoskeletal, mental and respiratory multimorbidity patterns (43, 49, 53, 54, 60, 61, 67, 68, 77, 108). At the same time, healthy behaviors such as physical activity seem to slow down the early appearance of these multimorbidity patterns or, at least, lessen their impact on the patient's quality of life (58, 59, 61, 68, 74, 75, 97). Likewise, although no studies have been found that specifically address the impact of diet on multimorbidity, the findings highlight the strong relationship between obesity and overweight with the appearance of the most prevalent patterns (cardiovascular and musculoskeletal) and subsequent mortality in the population (44, 60, 67, 68, 75, 108). In this sense, the need for specific studies to address the relationship between nutrition and multimorbidity (particularly with cancer-related patterns), as well as identifying preventive strategies to intervene in the problem of multimorbidity from an early age, is evident.

Although we have not found many studies that analyse the impact of social networks and personal relationships on multimorbidity, we have identified some works indicating the relevance of social connections in multimorbidity (61, 62, 97). On the other hand, although the impact of multimorbidity on the use of services is clearly reflected in the literature (149), no evidence has been found that points to the possible positive and/or adverse effects that the use of health services can have on specific patterns of multimorbidity. In this sense, we believe this line of work should be addressed in future research.

### 4.1. Limitations and strengths

Although one of our initial objectives was to be able to concisely compare the multimorbidity patterns identified in each investigation, the high heterogeneity of the data and classification techniques used, in addition to the fact that there is no general nomenclature to define the patterns, has not allowed us to synthesize the findings in a generic way, nor to estimate the prevalence of the different patterns. Due to the diversity of articles on multimorbidity and the variety of ways of measuring this health condition and its associated terms, it was difficult to identify an integrative definition of the concept that covered the full spectrum and complexity of mental and physical chronic conditions. Consequently, the pattern extraction methodology was diverse among the selected articles. In the grouping into five categories, the most frequent method was the LCA technique, and the least frequent was expert opinion, which seems to be a classification criterion that is progressively disappearing as new machine learning methods appear that enable the use of different levels of variable measurement, the use of data with high dimensionality and, consequently, facilitate the classification of complex multimorbidity patterns (139). In any case, despite this limitation, we consider this finding to be beneficial, since it highlights the need to work on future research that will make it possible to create a clear conceptual framework and an interoperable taxonomy of multimorbidity patterns in the field, as well as new methods that will allow us to better measure and compare chronicity profiles (146).

The present review has some advantages over previous work. On the one hand, this is the first time that a comprehensive review has been carried out that synthesizes the different classification methods used for the extraction of co-joint chronic disease patterns, together with a detailed description of the different typologies of multimorbidity described in the literature and their respective social and behavioral determinants (including individual sociodemographic and socioeconomic factors, lifestyles and risk behaviors, ethnic group, as well as contextual aspects such as social connectivity, place of residence, or use of health services). On the other hand, despite the difficulties in synthesizing the results of studies based on different populations, chronic pathologies and classification methods, the breadth of our work has allowed us to identify gaps in knowledge on topics that may be fundamental for future approaches to multimorbidity, such as chronicity patterns in children and young people or, for example, the influence of relationships and social networks on the emergence and evolution of chronic pluri-pathology.

## 5. Conclusions

The findings of the present review evidence the need for further studies addressing the impact of multimorbidity and its social determinants in population groups where this problem remains invisible (e.g., women, children, adolescents and young adults, ethnic groups, disabled population, older people living alone and/or with few social relations), as well as further work with more heterogeneous samples (i.e., not only focusing on older people) and using more robust methodologies for better classification and subsequent understanding of multimorbidity patterns. In addition, more studies focusing on the social determinants of multimorbidity and its inequalities are urgently needed in LMIC, where this problem is currently understudied. Finally, our results point to the need for the development of multidisciplinary multimorbidity research and new social and health policies tailored to address social inequalities in population groups with different needs, in order to improve health outcomes and increase the efficiency of health services at the local level.

## Author contributions

JÁ-G lead the DEMMOCAD project, conceived the study, interpreted the results, and developed the final version of the manuscript. JC-B, EO-M, CP-M, BR-F, and VS-L searched the different databases, extracted the data, assessed the results, and develop the first draft of the document. The manuscript, figures, and final tables were read and approved by all authors.
