# Supplementary material for: Social determinants of multimorbidity patterns: A systematic review
Source: Front Public Health. 2023 Mar 27;11:1081518. doi: 10.3389/fpubh.2023.1081518 (PMC10084932; doi:10.3389/fpubh.2023.1081518)
Supplement: Supplementary file 1 [file Table_1.DOCX]

Supplementary Material 1

# Table 1: Search strategy

1. Multimorbid* [All Fields]
2. multi-morbid* [All Fields]
3. “multiple morbid*” [All Fields]
4. “multiple chronic condition*” [All Fields]
5. “multiple chronic disease*” [All Fields]
6. “multiple chronic illnes*” [All Fields]
7. “multiple chronic patholog*” [All Fields]
8. “multiple diagnos*” [All Fields]
9. “several chronic condition*” [All Fields]
10. “several health problem*” [All Fields]
11. comorbid* [All Fields]
12. co-morbid* [All Fields]
13. “coexisting condition*” [All Fields]
14. “coexisting diagnos*” [All Fields]
15. “coexisting illnes*” [All Fields]
16. “coexisting patholog*” [All Fields]
17. “concurrent condition*” [All Fields]
18. “concurrent diagnos*” [All Fields]
19. “concurrent illnes*” [All Fields]
20. “concurrent patholog*” [All Fields]
21. polypatholog* [All Fields]
22. poly-patholog* [All Fields]
23. multipatholog* [All Fields]
24. multi-patholog* [All Fields]
25. pluripatholog* [All Fields]
26. pluri-patholog*[All Fields]
27. 1 or 2 or 3 or 4 or 5 or 6 or 7 or 8 or 9 or 10 or 11 or 12 or 13 or 14 or 15 or 16 or 17 or 18 or 19 or 20 or 21 or 22 or 23 or 24 or 25 or 26
28. Income* [All Fields]
29. savings ("income"[MeSH Terms] OR "income"[All Fields] OR "savings"[All Fields] OR "saved"[All Fields] OR "saves"[All Fields] OR "saving"[All Fields] )
30. “wealth index” [All Fields]
31. household ("family characteristics"[MeSH Terms] OR ("family"[All Fields] AND "characteristics"[All Fields]) OR "family characteristics"[All Fields] OR "household"[All Fields] OR "households"[All Fields] OR "household's"[All Fields] OR "householder"[All Fields] OR "householder's"[All Fields] OR "householders"[All Fields])
32. “financial problem” [All Fields]
33. "financial difficulties" [All Fields]
34. inequal* [All Fields]
35. inequit* [All Fields]
36. “social inequal*” [All Fields]
37. poverty ("poverty"[MeSH Terms] OR "poverty"[All Fields] OR "poverty's"[All Fields])
38. "social determinant*” [All Fields]
39. socioeconomic* [All Fields]
40. factor* [All Fields]
41. socio-economic* [All Fields]
42. disparit* [All Fields]
43. socio-demographic* [All Fields]
44. sociodemographic* [All Fields]
45. age ("Age"[Journal:__jid101090771] OR "Age (Omaha)"[Journal:__jid7801686] OR "Age (Dordr)"[Journal:__jid101250497] OR "Adv Genet Eng"[Journal:__jid101600827] OR "age"[All Fields])
46. sex ("sex"[MeSH Terms] OR "sex"[All Fields])
47. gender ("gender identity"[MeSH Terms] OR ("gender"[All Fields] AND "identity"[All Fields]) OR "gender identity"[All Fields] OR "gendered"[All Fields] OR "gender's"[All Fields] OR "gendering"[All Fields] OR "genderized"[All Fields] OR "genders"[All Fields] OR "sex"[MeSH Terms] OR "sex"[All Fields] OR "gender"[All Fields])
48. education* [All Fields]
49. “marital status” [All Fields]
50. marriage ("marriage"[MeSH Terms] OR "marriage"[All Fields] OR "marriages"[All Fields] OR "marriageability"[All Fields] OR "marriageable"[All Fields])
51. single* [All Fields]
52. widow* [All Fields]
53. divorce* [All Fields]
54. occupation* [All Fields]
55. labor ("labor's"[All Fields] OR "labored"[All Fields] OR "laborer"[All Fields] OR "laborer's"[All Fields] OR "laborers"[All Fields] OR "laboring"[All Fields] OR "labors"[All Fields] OR "labour"[All Fields] OR "work"[MeSH Terms] OR "work"[All Fields] OR "labor"[All Fields] OR "labor, obstetric"[MeSH Terms] OR ("labor"[All Fields] AND "obstetric"[All Fields]) OR "obstetric labor"[All Fields] OR "laboured"[All Fields] OR "labourer"[All Fields] OR "labourers"[All Fields] OR "labouring"[All Fields] OR "labours"[All Fields])
56. employment ("employability"[All Fields] OR "employable"[All Fields] OR "employer"[All Fields] OR "employer's"[All Fields] OR "employers"[All Fields] OR "employment"[MeSH Terms] OR "employment"[All Fields] OR "employments"[All Fields])
57. ethnia [All Fields]
58. ethnicity ("ethnical"[All Fields] OR "ethnically"[All Fields] OR "ethnicities"[All Fields] OR "ethnicity"[MeSH Terms] OR "ethnicity"[All Fields] OR "ethnic"[All Fields] OR "ethnics"[All Fields] OR "ethnology"[Subheading] OR "ethnology"[All Fields] OR "ethnology"[MeSH Terms])
59. “ethnic groups” [All Fields]
60. nationality ("ethnicity"[MeSH Terms] OR "ethnicity"[All Fields] OR "nationalities"[All Fields] OR "nationality"[All Fields] OR "federal government"[MeSH Terms] OR ("federal"[All Fields] AND "government"[All Fields]) OR "federal government"[All Fields] OR "national"[All Fields] OR "nation"[All Fields] OR "nation's"[All Fields] OR "nationalism"[All Fields] OR "nationalisms"[All Fields] OR "nationalization"[All Fields] OR "nationalized"[All Fields] OR "nationally"[All Fields] OR "nationals"[All Fields] OR "nations"[All Fields] OR "nations's"[All Fields])
61. migrat* [All Fields]
62. “skin color” [All Fields]
63. “skin colour” [All Fields]
64. neighbourhood* [All Fields]
65. neighborhood* [All Fields]
66. ghetto* [All Fields]
67. slum* [All Fields]
68. "census tracts" [All Fields]
69. region* [All Fields]
70. “residence characteristic*” [All Fields]
71. “residence area” [All Fields]
72. “income area” [All Fields]
73. “area deprivation” [All Fields]
74. “geographical area” [All Fields]
75. lifestyle ("life style"[MeSH Terms] OR ("life"[All Fields] AND "style"[All Fields]) OR "life style"[All Fields] OR "lifestyle"[All Fields] OR "lifestyles"[All Fields])
76. “quality of life” [All Fields]
77. HRQOL ("hrqols"[All Fields] OR "quality of life"[MeSH Terms] OR ("quality"[All Fields] AND "life"[All Fields]) OR "quality of life"[All Fields] OR "hrqol"[All Fields])
78. “life quality” [All Fields]
79. “social network*” [All Fields]
80. "community cohesion" [All Fields]
81. "community connect*" [All Fields]
82. "community network*" [All Fields]
83. "community support" [All Fields]
84. “health service*” [All Fields]
85. 28 or 29 or 30 or 31 or 32 or 33 or 34 or 35 or 36 or 37 or 38 or 39 or 40 or 41 or 42 or 43 or 44 or 45 or 46 or 47 or 48 or 49 or 50 or 51 or 52 or 53 or 54 or 55 or 56 or 57 or 58 or 59 or 60 or 61 or 62 or 63 or 64 or 65 or 66 or 67 or 68 or 69 or 70 or 71 or 72 or 73 or 74 or 75 or 76 or 77 or 78 or 79 or 80 or 81 or 82 or 83 or 84
86. pattern* [All Fields]
87. trend* [All Fields]
88. latent* [All Fields]
89. “class analysis” [All Fields]
90. LCA [All Fields]
91. “neural network*” [All Fields]
92. “bayesian network*” [All Fields]
93. cluster* [All Fields]
94. k-means [All Fields]
95. “fuzzy c-means” [All Fields]
96. hierarchic* [All Fields]
97. “self-organizing map” [All Fields]
98. SOM [All Fields]
99. conglomerate* [All Fields]
100. partition* [All Fields]
101. “factor analysis” [All Fields]
102. “mixture model*” [All Fields]
103. “finite mixture” [All Fields]
104. “non-random association” [All Fields]
105. “nonrandom association” [All Fields]
106. “observed/expected” [All Fields]
107. O/E (o/e[Author] OR o/e[Investigator])
108. 86 or 87 or 88 or 89 or 90 or 91 or 92 or 93 or 94 or 95 or 96 or 97 or 98 or 99 or 100 or 101 or 102 or 103 or 104 or 105 or 106 or 107
109. 27 and 85 and 108
110. limit 109 to (english and spanish language and yr=”2011-2021”)

| **Database** | **Search Strategy** | **Limit to** | **References** |
| --- | --- | --- | --- |
| PubMed and PsycINFO | (Multimorbid* OR multi-morbid* OR “multiple morbid*” OR “multiple chronic condition*” OR “multiple chronic disease*” OR “multiple chronic illnes*” OR “multiple chronic patholog*” OR “multiple diagnos*” OR “several chronic condition*” OR “several health problem*” OR comorbid* OR co-morbid* OR “coexisting condition*” OR “coexisting diagnos*” OR “coexisting illnes*” OR “coexisting patholog*” OR “concurrent condition*” OR “concurrent diagnos*” OR “concurrent illnes*” OR “concurrent patholog*” OR polypatholog* OR poly-patholog* OR multipatholog* OR multi-patholog* OR pluripatholog* OR pluri-patholog*) AND (Income* OR savings OR “wealth index” OR household OR “financial problem” OR “financial difficulties” OR inequal* OR inequit* OR “social inequal*” OR poverty OR “social determinant*” OR socioeconomic* OR factor* OR socio-economic* OR disparit* OR socio-demographic* OR sociodemographic* OR age OR sex OR gender OR education* OR “marital status” OR marriage OR single* OR widow* OR divorce* OR occupation* OR labor OR employment OR ethnia OR ethnicity OR “ethnic groups” OR nationality OR migrat* OR “skin color” OR “skin colour” OR neighbourhood* OR neighborhood* OR ghetto* OR slum* OR “census tracts” OR region* OR “residence characteristic*” OR “residence area” OR “income area” OR “area deprivation” OR “geographical area” OR lifestyle OR “quality of life” OR HRQOL OR “life quality” OR “social network*” OR “community cohesion” OR “community connect*” OR “community network*” OR “community support” OR “health service*”) AND (pattern* OR trend* OR latent* OR “class analysis” OR LCA OR “neural network*” OR “bayesian network*” OR cluster* OR k-means OR “fuzzy c-means” OR hierarchic* OR “self-organizing map” OR SOM OR conglomerate* OR partition* OR “factor analysis” OR “mixture model*” OR “finite mixture” OR “non-random association” OR “nonrandom association” OR “observed/expected” OR O/E) | English, Spanish, humans, 2011-2021 | 25,342 + 7,003 |
| Ovid MEDLINE | (Multimorbid* OR multi-morbid* OR “multiple morbid*” OR “multiple chronic condition*” OR “multiple chronic disease*” OR “multiple chronic illnes*” OR “multiple chronic patholog*” OR “multiple diagnos*” OR “several chronic condition*” OR “several health problem*” OR comorbid* OR co-morbid* OR “coexisting condition*” OR “coexisting diagnos*” OR “coexisting illnes*” OR “coexisting patholog*” OR “concurrent condition*” OR “concurrent diagnos*” OR “concurrent illnes*” OR “concurrent patholog*” OR polypatholog* OR poly-patholog* OR multipatholog* OR multi-patholog* OR pluripatholog* OR pluri-patholog*) AND (Income* OR savings OR “wealth index” OR household OR “financial problem” OR “financial difficulties” OR inequal* OR inequit* OR “social inequal*” OR poverty OR “social determinant*” OR socioeconomic* OR factor* OR socio-economic* OR disparit* OR socio-demographic* OR sociodemographic* OR age OR sex OR gender OR education* OR “marital status” OR marriage OR single* OR widow* OR divorce* OR occupation* OR labor OR employment OR ethnia OR ethnicity OR “ethnic groups” OR nationality OR migrat* OR “skin color” OR “skin colour” OR neighbourhood* OR neighborhood* OR ghetto* OR slum* OR “census tracts” OR region* OR “residence characteristic*” OR “residence area” OR “income area” OR “area deprivation” OR “geographical area” OR lifestyle OR “quality of life” OR HRQOL OR “life quality” OR “social network*” OR “community cohesion” OR “community connect*” OR “community network*” OR “community support” OR “health service*”) AND (pattern* OR trend* OR latent* OR “class analysis” OR LCA OR “neural network*” OR “bayesian network*” OR cluster* OR k-means OR “fuzzy c-means” OR hierarchic* OR “self-organizing map” OR SOM OR conglomerate* OR partition* OR “factor analysis” OR “mixture model*” OR “finite mixture” OR “non-random association” OR “nonrandom association” OR “observed/expected”) | English, Spanish, humans, 2011-2021 | 26,182 |
| Web Of Science, Scopus and Cinahl | (Multimorbid* OR multi-morbid* OR “multiple morbid*” OR “multiple chronic condition*” OR “multiple chronic disease*” OR “multiple chronic illnes*” OR “multiple chronic patholog*” OR “multiple diagnos*” OR “several chronic condition*” OR “several health problem*” OR comorbid* OR co-morbid* OR “coexisting condition*” OR “coexisting diagnos*” OR “coexisting illnes*” OR “coexisting patholog*” OR “concurrent condition*” OR “concurrent diagnos*” OR “concurrent illnes*” OR “concurrent patholog*” OR polypatholog* OR poly-patholog* OR multipatholog* OR multi-patholog* OR pluripatholog* OR pluri-patholog*) AND (Income* OR savings OR “wealth index” OR household OR “financial problem” OR “financial difficulties” OR inequal* OR inequit* OR “social inequal*” OR poverty OR “social determinant*” OR socioeconomic* OR factor* OR socio-economic* OR disparit* OR socio-demographic* OR sociodemographic* OR age OR sex OR gender OR education* OR “marital status” OR marriage OR single* OR widow* OR divorce* OR occupation* OR labor OR employment OR ethnia OR ethnicity OR “ethnic groups” OR nationality OR migrat* OR “skin color” OR “skin colour” OR neighbourhood* OR neighborhood* OR ghetto* OR slum* OR “census tracts” OR region* OR “residence characteristic*” OR “residence area” OR “income area” OR “area deprivation” OR “geographical area” OR lifestyle OR “quality of life” OR HRQOL OR “life quality” OR “social network*” OR “community cohesion” OR “community connect*” OR “community network*” OR “community support” OR “health service*”) AND (pattern* OR trend* OR latent* OR “class analysis” OR LCA OR “neural network*” OR “bayesian network*” OR cluster* OR k-means OR “fuzzy c-means” OR hierarchic* OR “self-organizing map” OR SOM OR conglomerate* OR partition* OR “factor analysis” OR “mixture model*” OR “finite mixture” OR “non-random association” OR “nonrandom association” OR “observed/expected” OR O/E) | English, Spanish, 2011-2021 | 33,254 + 31,930 + 11,924 |
| Embase | (Multimorbid* OR multi-morbid* OR “multiple morbid*” OR “multiple chronic condition*” OR “multiple chronic disease*” OR “multiple chronic illnes*” OR “multiple chronic patholog*” OR “multiple diagnos*” OR “several chronic condition*” OR “several health problem*” OR comorbid* OR co-morbid* OR “coexisting condition*” OR “coexisting diagnos*” OR “coexisting illnes*” OR “coexisting patholog*” OR “concurrent condition*” OR “concurrent diagnos*” OR “concurrent illnes*” OR “concurrent patholog*” OR polypatholog* OR poly-patholog* OR multipatholog* OR multi-patholog* OR pluripatholog* OR pluri-patholog*) AND (Income* OR savings OR “wealth index” OR household OR “financial problem” OR “financial difficulties” OR inequal* OR inequit* OR “social inequal*” OR poverty OR “social determinant*” OR socioeconomic* OR factor* OR socio-economic* OR disparit* OR socio-demographic* OR sociodemographic* OR age OR sex OR gender OR education* OR “marital status” OR marriage OR single* OR widow* OR divorce* OR occupation* OR labor OR employment OR ethnia OR ethnicity OR “ethnic groups” OR nationality OR migrat* OR “skin color” OR “skin colour” OR neighbourhood* OR neighborhood* OR ghetto* OR slum* OR “census tracts” OR region* OR “residence characteristic*” OR “residence area” OR “income area” OR “area deprivation” OR “geographical area” OR lifestyle OR “quality of life” OR HRQOL OR “life quality” OR “social network*” OR “community cohesion” OR “community connect*” OR “community network*” OR “community support” OR “health service*”) AND (pattern* OR trend* OR latent* OR “class analysis” OR LCA OR “neural network*” OR “bayesian network*” OR cluster* OR k-means OR “fuzzy c-means” OR hierarchic* OR “self-organizing map” OR SOM OR conglomerate* OR partition* OR “factor analysis” OR “mixture model*” OR “finite mixture” OR “non-random association” OR “nonrandom association” OR “observed/expected”) | English, Spanish, humans, 2011-2021 | 18,030 |
| TOTAL |  |  | 153,665 |
