# Supplementary material for: Social determinants of multimorbidity patterns: A systematic review
Source: Front Public Health. 2023 Mar 27;11:1081518. doi: 10.3389/fpubh.2023.1081518 (PMC10084932; doi:10.3389/fpubh.2023.1081518)
Supplement: Supplementary file 3 [file Table_3.DOCX]

Supplementary Material

# Table 3: Summary of studies and main findings

| TITLE | AUTHORS | YEAR | DOI | OBJECTIVES | CONCLUSIONS (AUTHOR) |
| --- | --- | --- | --- | --- | --- |
| A cluster-based approach for integrating clinical management of Medicare beneficiaries with multiple chronic conditions [78] | Egan, Brent M.; Sutherland, Susan E.; Tilkemeier, Peter L.; Davis, Robert A.; Rutledge, Valinda; Sinopoli, Angelo | 2019 | 10.1371/journal.pone.0217696 | To determine if a limited number of groups, based on similar within group patterns of ccs, could be identified in a relatively large group of Medicare beneficiaries. | Identifying a limited number of groups with high burdens of ccs that disproportionately drive costs may help inform a practical number of integrated guidelines and resources required for comprehensive management. Cluster informed guideline integration may improve care quality and outcomes, while reducing costs. |
| A Latent Class Analysis of Multimorbidity and the Relationship to Socio-Demographic Factors and Health-Related Quality of Life. A National Population-Based Study of 162,283 Danish Adults. [41] | Larsen, Finn Breinholt; Pedersen, Marie Hauge; Friis, Karina; Glümer, Charlotte; Lasgaard, Mathias | 2017 | 10.1371/journal.pone.0169426 | To identify patterns of multimorbidity in the general population and examine how these patterns are related to socio-demographic factors and health-related quality of life. | The results clearly support those diseases tend to compound and interact, which suggests that a differentiated public health and treatment approach towards multimorbidity is needed. |
| Age density patterns in patients medical conditions: A clustering approach. [79] | Alhasoun F, Aleissa F, Alhazzani M, Moyano LG, Pinhanez C, González MC. | 2018 | 10.1371/journal.pcbi.1006115 | To investigate the relationship of comorbidity coefficient values to the discovered clusters of conditions. | Our findings build prior knowledge related to age and sex for automated diagnostics in a Bayesian setting to predict the condition of a patient given their symptoms. |
| Analysis of multimorbidity networks associated with different factors in Northeast China: a cross-sectional analysis [119] | Yu J, Li Y, Zheng Z, Jia H, Cao P, Qiangba Y, Yu X. | 2021 | 10.1136/bmjopen-2021-051050 | To identify and study the associations and co-occurrence of multimorbidity, and assessed the associations of diseases with sex, age, and hospitalization duration. | CD, hyd, IHD and OFHD were the central points of disease clusters and directly or indirectly related to other diseases or factors. Thus, effective interventions for these diseases should be adopted. Furthermore, different intervention strategies should be developed according to multimorbidity patterns in different age groups. |
| Bayesian networks to identify potential high-risk multimorbidity and intervention clusters in inpatients: an explorative data mining study [120] | Roth, JA; Thomas, S; Martin, G; Hug, BL; PATREC Study Grp | 2020 | 10.44147/SMW.2020.20299 | To show high risks for in-hospital mortality and unplanned readmissions using data-driven analytical methods. | Bayesian network analysis may be used as a tool to mine large healthcare databases to explore intervention targets for quality improvement programs. |
| Burden of multimorbidity in relation to age, gender and immigrant status: a cross-sectional study based on administrative data [116] | Lenzi J, Avaldi VM, Rucci P, Pieri G, Fantini MP. | 2016 | 10.1136/bmjopen-2016-012812 | To estimate the prevalence of multimorbidity in a Northern Italian region, to investigate its distribution by age, gender, and citizenship and to analyze the correlations of diseases. | Multimorbidity was highly prevalent in Emilia-Romagna and strongly associated with age. This finding highlights the need for healthcare providers to adopt individualized care plans and ensure continuity of care. |
| Characteristics, service use and mortality of clusters of multimorbid patients in England: a population-based study [42] | Zhu Y, Edwards D, Mant J, Payne RA, Kiddle S. | 2020 | 10.1186/s12916-020-01543-8 | To identify clusters of multimorbidity patients and how they are differentially associated with mortality and service use across age groups in a population-representative sample | This work has highlighted patterns of multimorbidity that have implications for health services. These include the importance of psychoactive substance and alcohol misuse in people under the age of 65, of co-morbid depression and coronary heart disease in people aged 65–84 and of cardiovascular disease in people aged 85+ |
| Chronic condition patterns in the US population and their association with health related quality of life [43] | Zheng DD, McCollister KE, Christ SL, Lam BL, Feaster DJ, Lee DJ. | 2020 | 10.1016/j.ypmed.2020.106102 | To identify chronic disease patterns and their relationship to health-related quality of life (HRQL) in the US population | The relationship between physical and mental health functioning varied across different multi-morbidity groups, and the discordance was more pronounced in younger ages and females. Our research also identified an older age group that was mentally robust and maintained a strong HRQL. Findings can inform the development of targeted interventions to improve physical and mental health functioning in vulnerable populations. |
| Clustering of 27,525,663 death records from the united states based on health conditions associated with death: An example of big health data exploration [127] | Janssen, D.J.A., Rechberger, S., Wouters, E.F.M., Schols, J.M.G.A., Johnson, M.J., Currow, D.C., Curtis, J.R., Spruit, M.A. | 2019 | 10.3390/jcm8070922 | To cluster 27,525,663 deceased people based on the health conditions associated with death to study the associations between the health condition clusters, demographics, the recorded underlying cause and place of death | The prevalence of multiple health conditions at death requires a shift from disease-oriented towards person-centered palliative care at the end of life, including timely advance care planning. Understanding differences in population-based patterns and clusters of end-of-life experiences is an important step toward developing a strategy for implementing population-based palliative care. |
| Clustering of Mental and Physical Comorbidity and the Risk of Frailty in Patients Aged 60 Years or More in Primary Care. [80] | Bekić S, Babič F, Filipčić I, Trtica Majnarić L. | 2019 | 10.12659/MSM.915063 | To identify the clustering of comorbidities, cognitive, and mental factors associated with increased risk of pre-frailty and frailty in patients ≥60 years in a primary healthcare setting in eastern Croatia. | In patients ≥60 years in a primary healthcare setting, multimorbidity predictors of pre-frailty and frailty included a decline in cognitive function and renal function. |
| Clusters of Multiple Complex Chronic Conditions: A Latent Class Analysis of Children at End of Life [44] | Lindley, L.C., Mack, J.W., Bruce, D.J. | 2016 | 10.1016/j.jpainsymman.2015.12.310 | To understand the clusters of complex chronic conditions, present among children in the last year of life. | This analysis presented a novel way of understanding patterns of multiple complex chronic conditions among children that may inform tailored and targeted end-of-life care for different clusters. |
| Comorbidity patterns and socioeconomic inequalities in children under 15 with medical complexity: a population-based study [45] | Carrilero, Neus; Dalmau-Bueno, Albert; García-Altés, Anna | 2020 | 10.1186/s12887-020-02253-z | To describe the pathologic patterns of CMC and their socioeconomic inequalities to better manage their needs, plan healthcare services accordingly, and improve the care models in place. | Our findings show the existence of four different patterns of comorbidities in CMC and a significantly high proportion of lower SEP children in all classes. These results could benefit CMC management by creating more efficient multidisciplinary medical teams according to each comorbidity class and a holistic perspective considering its socioeconomic vulnerability. |
| Comorbidity profile of mental disorders among adolescents: A latent class analysis [46] | Essau CA, de la Torre-Luque A. | 2019 | 10.1016/j.psychres.2019.06.007 | To identify the number of comorbidity profiles among adolescents. Sociodemographic factors associated with the comorbidity profiles were also examined | Personalized protocols of assessments may help to uncover individual-specific markers which underpin disorder attenuation or exacerbation. Knowledge about comorbidity profile and related factors may provide therapeutic choices which in turn could influence treatment response and prognosis |
| Comorbidity Profiles Identified in Older Primary Care Patients Who Attempt Suicide [47] | Morin RT, Li Y, Mackin RS, Whooley MA, Conwell Y, Byers AL. | 2019 | 10.1111/jgs.16126 | To identify comorbidity profiles of older patients last seen in primary care before a suicide attempt and assess attempt and clinical factors (eg, means and lethality of attempt) associated with these profiles | This study provides evidence that most comorbidity profiles (>50%) in primary care patients attempting suicide were characterized by minimal depression diagnoses and fatal attempts, mostly with firearms. These findings suggest that more than a depression diagnosis contributes to risk and that conversations about firearm safety by medical providers may play an important role in suicide intervention and prevention. |
| Comparative analysis of methods for identifying multimorbidity patterns: a study of 'real-world' data [81] | Roso-Llorach A, Violán C, Foguet-Boreu Q, Rodriguez-Blanco T, Pons-Vigués M, Pujol-Ribera E, Valderas JM. | 2018 | 10.1136/bmjopen-2017-018986 | To compare multimorbidity patterns identified with the two most used methods: hierarchical cluster analysis (HCA) and exploratory factor analysis (EFA) in a large primary care database | This study showed that multimorbidity patterns vary depending on the method of analysis used (HCA vs EFA) and provided new evidence about the known limitations of attempts to compare multimorbidity patterns in real-world data studies. We found that EFA was useful in describing comorbidity relationships and HCA could be useful for in-depth study of multimorbidity. Our results suggest possible applications for each of these methods in clinical and research settings and add information about some aspects that must be considered in standardization of future studies: spectrum of diseases, data usage and methods of analysis. |
| Comparing Multimorbidity Patterns Among Discharged Middle-Aged and Older Inpatients Between Hong Kong and Zurich: A Hierarchical Agglomerative Clustering Analysis of Routine Hospital Records [82] | Lai, F.T.T., Beeler, P.E., Yip, B.H.K., Cheetham, M., Chau, P.Y.K., Chung, R.Y., Wong, E.L.Y., Yeoh, E.-K., Battegay, E., Wong, S.Y.S. | 2021 | 10.3389/fmed.2021.651925 | To derive and compare multimorbidity profiles in Hong Kong (HK, PRC) and Zurich (ZH, Switzerland) | His is the first study using hierarchical agglomerative clustering analysis to profile multimorbid inpatients from two different populations to identify universalities and differences of multimorbidity patterns. Our findings may inform the coordination of integrated/collaborative healthcare services. |
| Comparisons of disease cluster patterns, prevalence and health factors in the USA, Canada, England and Ireland [48] | Hernández, Belinda; Voll, Stacey; Lewis, Nathan A.; McCrory, Cathal; White, Arthur; Stirland, Lucy; Kenny, Rose Anne; Reilly, Richard; Hutton, Craig P.; Griffith, Lauren E.; Kirkland, Susan A.; Terrera, Graciela Muniz; Hofer, Scott M. | 2021 | 10.1186/s12889-021-11706-8 | To provide a cross-country comparison of disease prevalence as well as the unique patterns of multimorbidity as disease clusters and associated risk factors to uncover how differences in demographics, socio-economic status and health behaviors affect the combi-nations of diseases within and across four countries:United States, Canada, England, and Ireland. | The U.S. had significantly higher prevalence of multimorbidity and nearly all medical conditions studied compared to Canada, England and Ireland. This trend persisted even after controlling for age, sex, socio-economic and lifestyle factors. |
| Complex comorbidity clusters in OEF/OIF veterans: the polytrauma clinical triad and beyond [49] | Pugh MJ, Finley EP, Copeland LA, Wang CP, Noel PH, Amuan ME, Parsons HM, Wells M, Elizondo B, Pugh JA. | 2014 | 10.1097/MLR.0000000000000059 | To identify comorbidity clusters among diagnoses of deployment specific (TBI, PTSD, pain) and chronic (eg, hypertension, diabetes) conditions, and to examine the association of these clusters with health care utilization and adverse outcomes. | These comorbidity clusters extend beyond the PCT and may be used as a foundation to examine coordination/quality of care and outcomes for OEF/OIF Veterans with different patterns of comorbidity. |
| Contextual and individual inequalities of multimorbidity in Brazilian adults: a cross-sectional national-based study. [103] | Nunes, Bruno P.; Chiavegatto Filho, Alexandre D. P.; Pati, Sanghamitra; Cruz Teixeira, Doralice S.; Flores, Thaynã R.; Camargo-Figuera, Fabio A.; Munhoz, Tiago N.; Thumé, Elaine; Facchini, Luiz A.; Rodrigues Batista, Sandro R. | 2017 | 10.1136/bmjopen-2017-015885 | The study aims to evaluate the magnitude of multimorbidity in Brazilian adults, as well to measure their association with individual and contextual factors stratified by Brazilian states and regions. | In Brazil, at least 19million adults had multimorbidity. Frequency is like that found in other Low and Middle-Income Countries. Contextual and individual social inequalities were observed. |
| Deconstructing Complex Multimorbidity in the Very Old: Findings from the Newcastle 85+ Study [83] | Collerton J, Jagger C, Yadegarfar ME, Davies K, Parker SG, Robinson L, Kirkwood TB. | 2016 | 10.1155/2016/8745670 | To examine the extent and complexity of the morbidity burden in 85-year-olds; identify patterns within multimorbidity; and explore associations with medication and healthcare use | The majority of 85- year-olds had extensive and complex morbidity. Elaborating participant clusters sharing similar morbidity profiles will help inform future healthcare provision and the identification of common underlying biological mechanisms |
| Description of multimorbidity clusters of admitted patients in medical departments of a general hospital [84] | Matesanz-Fernández, M., Seoane-Pillado, T., Iñiguez-Vázquez, I., Suárez-Gil, R., Pértega-Díaz, S., Casariego-Vales, E. | 2021 | 10.1136/postgradmedj-2020-139361 | To identify patterns of disease clusters among inpatients of a general hospital and to describe the characteristics and evolution of each group | We identify for the first time in a hospital environment five clusters of disease combinations among the inpatients. These clusters contain several high incidence diseases related to both age and gender that express their own evolution and clinical characteristics over time |
| Differences in Clinical Outcomes of Adults Referred to a Homeless Transitional Care Program Based on Multimorbid Health Profiles: A Latent Class Analysis [50] | Smith, C.M., Feigal, J., Sloane, R., Biederman, D.J. | 2021 | 10.3389/fpsyt.2021.780366 | To delineate unique groups of individuals based on medical, psychiatric, and substance use disorder profiles, and compare clinical outcomes across groups | These data suggest that distinct groups of people experiencing homelessness are affected differently by comorbidities, thus health care programs for this population should address their risk factors accordingly |
| Differences in psychiatric comorbidities and gender distribution among three clusters of personality disorders: A nationwide population-based study [133] | Hsu, C.-W., Wang, L.-J., Lin, P.-Y., Hung, C.-F., Yang, Y.-H., Chen, Y.-M., Kao, H.-Y. | 2021 | 10.3390/jcm10153294 | To investigate the distribution pattern across the three clusters of pds with a population-based cohort study | The gender subgroups revealed significant male predominance in neurodevelopmental disorders and female predominance in sleep–wake disorders across all three clusters of pds. Our findings support that some psychiatric comorbidities are more prevalent in specified cluster pds and that gender differences exist across the three clusters of pds. |
| Disability, quality of life and all-cause mortality in older Mexican adults: association with multimorbidity and frailty [114] | Rivera-Almaraz A, Manrique-Espinoza B, Ávila-Funes JA, Chatterji S, Naidoo N, Kowal P, Salinas-Rodríguez A. | 2018 | 10.1186/s12877-018-0928-7 | To assess the association of frailty and multimorbidity with the disability, quality of life and all-cause mortality as well as to analyze a potential interaction between these conditions | Multimorbidity and frailty are important predictors of poor health outcomes. These results highlight the importance of carrying out health promotion and prevention actions as well as specific interventions aimed at older adults who suffer from multimorbidity and frailty, in such a way that deleterious effects on health can be avoided. |
| Effect of Multimorbidity on Health-Related Quality of Life in Adults Aged 55 Years or Older: Results from the SU.VI.MAX 2 Cohort [104] | Walker V, Perret-Guillaume C, Kesse-Guyot E, Agrinier N, Hercberg S, Galan P, Assmann KE, Briançon S, Rotonda C. | 2016 | 10.1371/journal.pone.0169282 | To describe overall multimorbidity patterns in adults aged 55 years or older and assess their effect on health-related quality of life (hrqol) | Our study used a novel methodological approach to account for multimorbidity patterns in determining the link with chronic conditions. These multimorbidity scores (counted and weighted) can be used in clinical research to control for the effect of multimorbidity on patients’ hrqol and may be useful for clinical practice |
| Ethnic and geographic variations in multimorbidty: Evidence from three large cohorts [51] | Gebregziabher, Mulugeta; Ward, Ralph C.; Taber, David J.; Walker, Rebekah J.; Ozieh, Mukoso; Dismuke, Clara E.; Axon, Robert N.; Egede, Leonard E. | 2018 | 10.1016/j.socscimed.2018.06.020 | To examine the magnitude and patterns of multimorbidity by race/ethnicity and geography; and compare the level of variation explained by these factors in three multimorbidity measures across three large cohorts | Multimorbidity risk was higher for nhb in urban areas compared to rural areas in all three cohorts; multimorbidity risk was higher for hispanics in urban areas compared to rural areas in the dm and ckd cohorts; and the highest overall multimorbidity risk of any race group or location exists for hispanics in insular islands for all three disease cohorts |
| Ethnicity and psychiatric comorbidity in a national sample: evidence for latent comorbidity factor invariance and connections with disorder prevalence [102] | Eaton, Nicholas R.; Keyes, Katherine M.; Krueger, Robert F.; Noordhof, Arjen; Skodol, Andrew E.; Markon, Kristian E.; Grant, Bridget F.; Hasin, Deborah S. | 2013 | 10.1007/s00127-012-0595-5 | To understand the nature of ethnicity-related health disparities in mental health | These findings, taken together, indicated that observed prevalence rate differences between ethnic groups reflect ethnic differences in latent internalizing and externalizing factor means. We discuss implications for classification (DSM-5 and ICD-11 meta-structure), health disparities research, and treatment. |
| Examining health disparities by gender: A multimorbidity network analysis of electronic medical record [121] | Kalgotra, Pankush; Sharda, Ramesh; Croff, Julie M. | 2017 | 10.1016/j.ijmedinf.2017.09.014 | To address the co-occurrences of diseases using network analysis. | Our multimorbidity network analysis by gender identifies specific differences in disease diagnosis by gender, and presents questions for biological, behavioral, clinical, and policy research. |
| Examining multimorbidity differences across racial groups: a network analysis of electronic medical records [122] | Kalgotra, Pankush; Sharda, Ramesh; Croff, Julie M. | 2020 | 10.1038/s41598-020-70470-8 | To study health disparities by identifying the networks of multimorbidities among individuals from seven population groups based on race, including White, African American, Asian, Hispanic, Native American, Bi- or Multi-racial and Pacific Islander | Our multimorbidity network analysis identifies specific differences in diagnoses among different population groups, and presents questions for biological, behavioral, clinical, social science, and policy research. |
| Factors Associated With Multimorbidity Patterns in Older Adults in England: Findings From the English Longitudinal Study of Aging (ELSA) [52] | Nguyen, Hai; Chua, Kia-Chong; Dregan, Alexandru; Vitoratou, Silia; Bayes-Marin, Ivet; Olaya, Beatriz; Prina, A. Matthew | 2020 | 10.1177/0898264319891026 | To identify the patterns of multimorbidity in older adults and explored their association with sociodemographic and lifestyle risk factors | Future research should investigate these patterns further to gain more insights into the needs of people with multimorbidity. |
| General practitioners records are epidemiological predictors of comorbidities: An analytical cross-sectional 10-year retrospective study [123] | Cavallo, P., Pagano, S., De Santis, M., Capobianco, E. | 2018 | 10.3390/jcm7080184 | To study comorbidity patterns in a general population, focusing on diabetic and non-diabetic patients. | GPR are usually combined with other data types in EHR studies, but we have shown that prescription data have value as standalone predictive tools, useful to anticipate trends observed at epidemiological level on large populations. This study is thus relevant to policy makers seeking inference tools for an efficient use of massive administrative database resources, and suggests a strategy for detecting comorbidities and investigating their evolution. |
| Global Multimorbidity Patterns: A Cross-Sectional, Population-Based, Multi-Country Study [105] | Garin, Noe; Koyanagi, Ai; Chatterji, Somnath; Tyrovolas, Stefanos; Olaya, Beatriz; Leonardi, Matilde; Lara, Elvira; Koskinen, Seppo; Tobiasz-Adamczyk, Beata; Ayuso-Mateos, Jose Luis; Haro, Josep Maria | 2016 | 10.1093/gerona/glv128 | To identify and describe multimorbidity patterns in low-, middle-, and high-income countries | A high prevalence of multimorbidity occurs in older adults across countries, with low- and middle-income countries gradually approaching the figures of richer countries. Certain multimorbidity patterns are present in several countries, which suggest that common underlying etiopathogenic factors may play a role. Deeper understanding of these patterns may lead to the development of preventive actions to diminish their prevalence and give rise to new, comprehensive approaches for the management of these co-occurring conditions |
| Identifying co-occurrence and clustering of chronic diseases using latent class analysis: cross-sectional findings from SAGE South Africa Wave 2 [53] | Chidumwa, Glory; Maposa, Innocent; Corso, Barbara; Minicuci, Nadia; Kowal, Paul; Micklesfield, Lisa K.; Ware, Lisa Jayne | 2021 | 10.1136/bmjopen-2020-041604 | To classify South African adults with chronic health conditions for multimorbidity (MM) risk, and to determine sociodemographic, anthropometric, and behavioral factors associated with identified patterns of MM, using data from the WHO’s Study on global Ageing and adult health South Africa Wave 2. | Ncds with similar pathophysiological risk profiles tend to cluster together in older people. Risk factors for MM in South African adults include sex, age and tobacco use. |
| Identifying longitudinal clusters of multimorbidity in an urban setting: A population-based cross-sectional study [85] | Bisquera, A., Gulliford, M., Dodhia, H., Ledwaba-Chapman, L., Durbaba, S., Soley-Bori, M., Fox-Rushby, J., Ashworth, M., Wang, Y. | 2021 | 10.1016/j.lanepe.2021.100047 | To identify ltcs which tend to cooccur, in an inner-city primary care setting and to find groups of conditions that are as correlated as possible among themselves and with as little correlation as possible with other groups in the data using Multiple correspondence analysis (MCA), a statistical technique to analyze clustering of multimorbidity | This study has identified the co-morbidity between substance/ alcohol dependency and HIV; liver disease and viral hepatitis; anxiety and depression; cardiometabolic diseases and chronic pain; heart conditions and dementia. These key relationships characterize the young urban population of south London. When considering interventions or medications for one condition, clinicians should account for the increased risk of the patient belonging to one cluster acquiring other ltcs within the same cluster |
| Identifying multimorbidity patterns of non-communicable diseases in paediatric inpatients: a cross-sectional study in Shanghai, China [130] | Chen N, Zhou L, Huang J, Yu W, Chen C, Jin H, Shi X, Yu Z, Liu Q, Yang Y, Wang Z, Shi J. | 2021 | 10.1136/bmjopen-2020-042679 | To enhance the understanding of noncommunicable disease (NCD) multimorbidity in children who are inpatients by delineating the characteristics of and identifying patterns among pediatric inpatients with multimorbidity in China. | Multimorbidity in pediatric inpatients suggests that decisions about reasonable allocation of pediatric inpatient resources should be fully considered. Multimorbidity patterns in pediatric inpatients revealed that prevention, including innovative treatments targeting children, should be further studied. |
| Identifying Patterns of Multimorbidity in Older Americans: Application of Latent Class Analysis [54] | Whitson HE, Johnson KS, Sloane R, Cigolle CT, Pieper CF, Landerman L, Hastings SN. | 2016 | 10.1111/jgs.14201 | To define multimorbidity “classes” empirically based on patterns of disease co-occurrence in older Americans and to examine how class membership predicts healthcare use. | Although recognition of general patterns of disease co-occurrence is useful for policy planning, the heterogeneity of persons with significant multimorbidity (≥3 conditions) defies neat classification. A simple count of conditions may be preferable for predicting usage. |
| Inequalities in multimorbidity among elderly: a population-based study in a city in Southern Brazil [106] | Dos Santos Costa, Caroline; Flores, Thaynã Ramos; Wendt, Andrea; Neves, Rosália Garcia; Tomasi, Elaine; Cesar, Juraci A.; Bertoldi, Andrea Dâmaso; Ramires, Virgílio Viana; Nunes, Bruno Pereira | 2018 | 10.1590/0102-311X00040718 | The aim of this study was to identify the prevalence of multimorbidity and clusters of health conditions among elderly, in relation to the underlying socioeconomic inequalities. | Three clusters of health conditions were found, involving musculoskeletal/ mental/functional disorders, cardiometabolic, and respiratory factors. Higher inequalities were found the higher amount of health conditions (5+), when considering economic level, and for 3+, 4+ and 5+, when considering educational level. These findings show high multimorbidity prevalence among elderly, highlighting the persistence of health inequalities in Southern Brazil. Strategies by the health services need to focus on elderly at lower socioeconomic levels. |
| Latent class analysis of multimorbidity patterns and associated outcomes in Spanish older adults: a prospective cohort study [55] | Olaya, Beatriz  Moneta, María Victoria  Caballero, Francisco Félix, Tyrovolas, Stefanos, Bayes, Ivet Ayuso-Mateos, José Luis, Haro, Josep Maria | 2017 | 10.1186/s12877-017-0586-1 | To identify multimorbidity patterns and determine the association between these latent classes with several outcomes, including health, functioning, disability, quality of life and use of services, at baseline and after 3 years of follow-up. | Common chronic conditions in older people cluster together in broad categories. These broad clusters are qualitatively distinct and are important predictors of several health and functioning outcomes. Future studies are needed to understand underlying mechanisms and common risk factors for patterns of multimorbidity and to propose more effective treatments. |
| Learning multimorbidity patterns from electronic health records using Non-negative Matrix Factorisation [128] | Hassaine A, Canoy D, Solares JRA, Zhu Y, Rao S, Li Y, Zottoli M, Rahimi K, Salimi-Khorshidi G. | 2020 | 10.1016/j.jbi.2020.103606 | To treat these clusters as nodes and use their time-courses to define their connectivity | Lastly, there have been various developments in methods related to our study that can provide multiple new directions for future works. For instance, deep learning’s success in the past few years has led to “deep phenotyping” research on EHR; while such models can help the study of MPs, their use has been limited to learning disease representations (or embeddings) for disease/event predictions. |
| Lifestyle and Socioeconomic Determinants of Multimorbidity Patterns among Mid-Aged Women: A Longitudinal Study [107] | Jackson, Caroline A.; Dobson, Annette J.; Tooth, Leigh R.; Mishra, Gita D. | 2016 | 10.1371/journal.pone.0156804 | Sought to ascertain patterns of associative multimorbidity in a cohort of mid aged women and to identify the associations between lifestyle and socioeconomic factors at baseline with the subsequent development of these multimorbidity patterns. | In conclusion, our study contributes to understanding the nature of multimorbidity among mid-aged women by identifying which conditions group together and which lifestyle and socioeconomic factors are potentially involved in the etiology of these multimorbidity. Patterns. In terms of preventive approaches among mid-aged women, improving physical activity levels and reducing the proportion of women who are overweight or obese may be the most appropriate approach to reducing the risk of these disease groups. |
| Multilevel Analysis of the Patterns of Physical-Mental Multimorbidity in General Population of São Paulo Metropolitan Area, Brazil [100] | Wang, Yuan-Pang; Nunes, Bruno P.; Coêlho, Bruno M.; Santana, Geilson L.; do Nascimento, Carla F.; Viana, Maria Carmen; Benseñor, Isabela M.; Andrade, Laura H.; Chiavegatto Filho, Alexandre D. P. | 2019 | 10.1038/s41598-019-39326-8 | We aim to investigate patterns of multimorbidity in the non-elderly general population of the metropolitan area of São Paulo26, located in a middle-income country. We also examine the association of multimorbidity with health-care utilization, as well as the determinants of individual- and area-level variables. | Multilevel analyses showed associations between multimorbidity patterns and both individual- and area-level determinants. Our fundings call for a reformulation of health-care systems worldwide, especially in low-resource countries. Replacing the single-disease framework by multi-disease patterns in health-care settings can improve the ability of general practitioners in the healthcare of person-centered needs. |
| Multimorbidity among two million adults in China [86] | Wang, X., Yao, S., Wang, M., Cao, G., Chen, Z., Huang, Z., Wu, Y., Han, L., Xu, B., Hu, Y. | 2020 | 10.3390/ijerph17103395 | To explore the multimorbidity prevalence and patterns among middle-aged and older adults from China | Visual impairment clusters, a mixed cluster of OARA, IHD, COPD, and cardiometabolic clusters were detected. Multimorbidity is prevalent among middle-aged and older Chinese individuals. The observations of multimorbidity patterns have implications for improving preventive care and developing appropriate guidelines for morbidity treatment. |
| Multimorbidity Analysis of 13 Systemic Diseases in Northeast China [124] | Yu, JX; Song, FY; Li, YY; Zheng, Z; Jia, HH; Sun, YZ; Jin, LN; Yu, XH | 2020 | 10.3390/ijerph17061817 | To reveal the connections between diseases, especially the important role each disease played in the entire multimorbidity network | Multimorbidity with respiratory system diseases in young people should not be overlooked. Additionally, effective prevention efforts that target endocrine, nutritional, and metabolic diseases and circulatory system diseases are needed in middle aged and old people |
| Multimorbidity and functional status in older people: a cluster analysis [87] | Machón M, Mateo-Abad M, Clerencia-Sierra M, Güell C, Poblador-Pou B, Vrotsou K, Gimeno-Miguel A, Prados-Torres A, Vergara I. | 2020 | 10.1007/s41999-020-00291-5 | To identify clusters of chronic diseases in robust and frail individuals and compare the sociodemographic and health characteristics between these clusters. | This exploratory study may provide relevant information for the clinical management of older patients with multimorbidity, even though the chronic disease clusters identified were similar in robust and frail individuals. |
| Multimorbidity and health-related quality of life (HRQoL) in a nationally representative population sample: implications of count versus cluster method for defining multimorbidity on HRQoL [88] | Wang L, Palmer AJ, Cocker F, Sanderson K. | 2017 | 10.1186/s12955-016-0580-x | To examine the performance of the count and cluster definitions of multimorbidity on the sociodemographic profile and health-related quality of life (hrqol) in a general population. | Our findings confirm the existence of an inverse relationship between multimorbidity and hrqol in the Australian population and indicate that the hierarchical clustering approach is validated when the outcome of interest is hrqol from this head-to-head comparison. Moreover, a simple count fails to identify if there are specific conditions of interest that are driving poorer hrqol. Researchers should exercise caution when selecting a definition of multimorbidity because it may significantly influence the study outcomes. |
| Multimorbidity and Hospital Admissions in High-Need, High-Cost Elderly Patients [56] | Buja, Alessandra; Rivera, Michele; De Battisti, Elisa; Corti, Maria Chiara; Avossa, Francesco; Schievano, Elena; Rigon, Stefano; Baldo, Vincenzo; Boccuzzo, Giovanna; Ebell, Mark H. | 2020 | 10.1177/0898264318817091 | To clarify which pairs or clusters of diseases predict the hospital-related events and death in a population of patients with complex health care needs (PCHCN) | Unlike morbidity counts, analyzing morbidity clusters and dyads reveals which combinations of morbidities are associated with the highest hospitalization rates or death. |
| Multimorbidity and Its Patterns according to Immigrant Origin. A Nationwide Register-Based Study in Norway [108] | Diaz, Esperanza; Poblador-Pou, Beatriz; Gimeno-Feliu, Luis-Andrés; Calderón-Larrañaga, Amaia; Kumar, Bernadette N.; Prados-Torres, Alexandra | 2015 | 10.1371/journal.pone.0145233 | This nationwide multi-register study in Norway enabled us i) to study the associations between multimorbidity and immigrant status as classified by area of origin, accounting for other known risk factors for multimorbidity, and ii) to identify patterns of multimorbidity in Norway for immigrants and Norwegian-born at different ages. Based on the previously described existing theories and on our earlier studies on use of health care services in Norway. | Our study confirmed the associations between multimorbidity and immigrant’s area of origin. Immigrants showed a lower prevalence of multimorbidity compared to Norwegian-born, despite the former frequently having lower socio economic and literacy levels. The similarities regarding the type and composition of the multimorbidity patterns found in both groups confirm the common physio pathological basis of diseases. The greater complexity of multimorbidity patterns for some immigrant groups requires further investigation. These complexities imply that health care policies and practice will require a more holistic approach for specific population groups to meet their health needs and curb and prevent diseases. |
| Multimorbidity gender patterns in hospitalized elderly patients [129] | Almagro P, Ponce A, Komal S, de la Asunción Villaverde M, Castrillo C, Grau G, Simon L, de la Sierra A. | 2020 | 10.1371/journal.pone.0227252 | To evaluate differences in patterns of multimorbidity by gender in this population and their possible prognostic implications, measured as in-hospital mortality, 1-month readmissions, and 1-year mortality. | Our study shows differing patterns of multimorbidity by gender, with greater functional impairment in women and more comorbidity in men, although without differences in the prognosis. Moreover, some of these prognostic indicators had differing accuracy for the genders in predicting mortality. |
| Multimorbidity in the community-dwelling elderly in urban China [117] | Gu J, Chao J, Chen W, Xu H, Wu Z, Chen H, He T, Deng L, Zhang R. | 2017 | 10.1016/j.archger.2016.09.001 | To investigate the prevalence and patterns of multimorbidity in the community-dwelling elderly in urban China. | A significant proportion of elderly populations was affected by multimorbidity in urban China. Specific patterns of multimorbidity were found at group level and the prevalence was associated with age. |
| Multimorbidity Patterns and 6-Year Risk of Institutionalization in Older Persons: The Role of Social Formal and Informal Care [89] | Marengoni, A., Tazzeo, C., Calderón-Larrañaga, A., Roso-Llorach, A., Onder, G., Zucchelli, A., Rizzuto, D., Vetrano, D.L. | 2021 | 10.1016/j.jamda.2020.12.040 | To evaluate patterns of multimorbidity that increase the risk of institutionalization in older persons, also exploring the potential buffering effect of formal and informal care. | Older persons suffering from specific multimorbidity patterns have a higher risk of institutionalization, especially if they lack formal or informal care. Interventions aimed at preventing the clustering of diseases could reduce the associated burden on residential long-term care. Formal and informal care provision may be effective strategies in reducing the risk of institutionalization. |
| Multimorbidity Patterns and Memory Trajectories in Older Adults: Evidence From the English Longitudinal Study of Aging [57] | Bendayan R, Zhu Y, Federman AD, Dobson RJB. | 2021 | 10.1093/gerona/glab009 | To examine the multimorbidity patterns within a representative sample of UK older adults and their association with concurrent and subsequent memory | These findings suggest that individuals with certain combinations of health conditions are more likely to have lower levels of memory compared to those with no multimorbidity and their memory scores tend to differ between combinations. Sociodemographic and health behaviors have a key role to understand who is more likely to be at risk of an accelerated decline. |
| Multimorbidity patterns and risk of frailty in older community-dwelling adults: a population-based cohort study [77] | Tazzeo C, Rizzuto D, Calderón-Larrañaga A, Roso-Llorach A, Marengoni A, Welmer AK, Onder G, Trevisan C, Vetrano DL. | 2021 | 10.1093/ageing/afab138 | To examine the cross-sectional and longitudinal associations of different multimorbidity patterns with physical frailty in older adults | We found that older adults with multimorbidity characterized by cardiovascular and neuropsychiatric disease patterns are most susceptible to developing physical frailty. |
| Multimorbidity patterns and their related characteristics in European older adults: A longitudinal perspective [58] | Zacarías-Pons, Lluís; Vilalta-Franch, Joan; Turró-Garriga, Oriol; Saez, Marc; Garre-Olmo, Josep | 2021 | 10.1016/j.archger.2021.104428 | To identify multimorbidity patterns and their related characteristics from a longitudinal perspective. | We reported information of multimorbidity classes and their characteristics that may help to develop targeted health strategies. Within a time window of four years, the identified latent classes were consistent between time points. |
| Multimorbidity patterns and their relationship to mortality in the US older adult population [59] | Zheng DD, Loewenstein DA, Christ SL, Feaster DJ, Lam BL, McCollister KE, Curiel-Cid RE, Lee DJ. | 2021 | 10.1371/journal.pone.0245053 | To employ LCA techniques to identify patterns of multimorbidity in the US 50 years and older population and evaluate their relationships with all-cause and cause-specific mortalities by utilizing the recent linkage between the National Health Interview Survey (NHIS) and the National Death Index (NDI) | We demonstrated that LCA method is effective in classifying clinically meaningful multimorbidity subgroup. Specific combinations of conditions including cognitive impairment and depressive symptoms have a substantial detrimental impact on the mortality of older adults. The numbers of chronic conditions experienced by older adults is not always proportional to mortality risk. Our findings provide valuable information for identifying high risk older adults with multimorbidity to facilitate early intervention to treat chronic conditions and reduce mortality. |
| Multimorbidity Patterns in Elderly Primary Health Care Patients in a South Mediterranean European Region: A Cluster Analysis [90] | Foguet-Boreu, Quinti; Violan, Concepcion; Rodriguez-Blanco, Teresa; Roso-Llorach, Albert; Pons-Vigues, Mariona; Pujol-Ribera, Enriqueta; Cossio Gil, Yolima; Valderas, Jose M. | 2015 | 10.1371/journal.pone.0141155 | The purpose of this study was to identify clusters of diagnoses in elderly patients with MM in the primary health care system in Catalonia, by sex and age group (65–79 years and 80 years). | We identified several clusters of diagnoses that are most prevalent by age group and sex in older adults. Some of these clusters were not previously observed but show a high degree of consistency across all strata. This study included a broad range of diagnoses, and corroborated. Some clusters of diseases that do not co-occur by chance. In all strata, hypertensive diseases and metabolic disorders consistently made up the most prevalent cluster, followed by the musculoskeletal diseases cluster. The results of this study offer the opportunity to shape future research on combined preventive measures for the different conditions within a given cluster and to inform clinical practice guidelines as well as diagnostic procedures and algorithms in the primary care setting. In summary, the identification of MM patterns facilitates the holistic approach to health care, focusing not only on a specific disease, but on the whole person and health promotion. The results of our study add knowledge to encourage this paradigm shift in health care. |
| Multimorbidity patterns in low-middle and high income regions: a multiregion latent class analysis using ATHLOS harmonised cohorts [60] | Bayes-Marin, Ivet; Sanchez-Niubo, Albert; Egea-Cortés, Laia; Nguyen, Hai; Prina, Matthew; Fernández, Daniel; Haro, Josep Maria; Olaya, Beatriz | 2020 | 10.1136/bmjopen-2019-034441 | Our aim was to determine clusters of non- communicable diseases (ncds) in a very large, population-based sample of middle-aged and older adults from low- and middle-income (lmics) and high-income (hics) regions. Additionally, we explored the associations with several covariates. | The results of this study suggest that NCDS cluster together in non-random associations across several regions worldwide. The three qualitatively distinct entities are also linked to several socio- demographic and economic characteristics, lifestyles and health status variables. A deeper understanding of the interactions across regions and the studied variables is needed. Knowledge regarding broad patterns of conditions may contribute to the creation and implementation of guidelines that consider clusters of conditions instead of single diseases, since multimorbidity has become an unavoidable reality. Future efforts should focus on the underlying mechanisms of these clusters as well as their stability over time using longitudinal data. Moreover, cohort and age effects should be explored as might influence the likelihood of reporting some diagnosis and hence result in different multimorbidity patterns. |
| Multimorbidity patterns in old adults and their associated multi-layered factors: a cross-sectional study [61] | Lu, Jiao; Wang, Yuan; Hou, Lihong; Zuo, Zhenxing; Zhang, Na; Wei, Anle | 2021 | 10.1186/s12877-021-02292-w | To determine the multi-layered factors associated with their various multimorbidity patterns. | In management of old patients with multimorbidity, we should prioritize both the “lifestyle change”- centered systematic management strategy and group-customized intervention programs. |
| Multimorbidity patterns in primary care: interactions among chronic diseases using factor analysis [101] | Prados-Torres A, Poblador-Plou B, Calderón-Larrañaga A, Gimeno-Feliu LA, González-Rubio F, Poncel-Falcó A, Sicras-Mainar A, Alcalá-Nalvaiz JT. | 2012 | 10.1371/journal.pone.0032190 | To identify the existence of chronic disease multimorbidity patterns in the primary care population, describing their clinical components and analyzing how these patterns change and evolve over time both in women and men | Non-random associations between chronic diseases result in clinically consistent multimorbidity patterns affecting a significant proportion of the population. Underlying pathophysiological phenomena were observed upon which action can be taken both from a clinical, individual-level perspective and from a public health or population-level perspective. |
| Multimorbidity patterns in the elderly: a prospective cohort study with cluster analysis [91] | Guisado-Clavero, M., Roso-Llorach, A., López-Jimenez, T. et al. | 2018 | 10.1186/s12877-018-0705-7 | To identify multimorbidity patterns over a six-year study period in electronic health records from a Mediterranean urban population older than 65 years and with multimorbidity, attended in primary health care centres in Barcelona (Spain). | This study identified six multimorbidity patterns per each group, one nonspecific pattern and five of them with a specific pattern related to an organic system. The multimorbidity patterns obtained had similar characteristics throughout the study period. These data are useful to improve clinical management of each specific subgroup of patients showing a particular multimorbidity pattern |
| Multimorbidity Patterns in the General Population: Results from the EpiChron Cohort Study [109] | Ioakeim-Skoufa I, Poblador-Plou B, Carmona-Pírez J, Díez-Manglano J, Navickas R, Gimeno-Feliu LA, González-Rubio F, Jureviciene E, Dambrauskas L, Prados-Torres A, Gimeno-Miguel A. | 2020 | 10.3390/ijerph17124242 | To analyze the existence of multimorbidity patterns in the general population based on gender and age | Our results demonstrate the potential of using real-world data to conduct large-scale epidemiological studies to assess the complex interactions among chronic conditions. This could be useful in designing clinical interventions for patients with multimorbidity, as well as recommendations for healthcare professionals on how to handle these types of patients in clinical practice. |
| Multimorbidity patterns of chronic conditions and geriatric syndromes in older patients from the MoPIM multicentre cohort study [92] | Baré M, Herranz S, Roso-Llorach A, Jordana R, Violán C, Lleal M, Roura-Poch P, Arellano M, Estrada R, Nazco GJ; MoPIM study group. | 2021 | 10.1136/bmjopen-2021-049334 | To estimate the frequency of chronic conditions and geriatric syndromes in older patients admitted to hospital because of an exacerbation of their chronic conditions, and to identify multimorbidity clusters in these patients. | In older patients admitted to hospital because of the exacerbation of chronic health problems, it is possible to define multimorbidity clusters using soft clustering techniques. These clusters are clinically relevant and could be the basis to reorganise healthcare circuits or processes to tackle the increasing number of older, multimorbid patients. |
| Multimorbidity patterns with K-means nonhierarchical cluster analysis [93] | Violán, C., Roso-Llorach, A., Foguet-Boreu, Q. et al. | 2018 | 10.1186/s12875-018-0790-x | The purpose of this study was to ascertain multimorbidity patterns using a non-hierarchical cluster analysis in adult primary patients with multimorbidity attended in primary care centers in Catalonia. | Non-hierarchical cluster analysis identified multimorbidity patterns consistent with clinical practice, identifying phenotypic subgroups of patients. |
| Multimorbidity patterns, all-cause mortality and healthy aging in older English adults: Results from the English Longitudinal Study of Aging [62] | Nguyen H, Wu YT, Dregan A, Vitoratou S, Chua KC, Prina AM. | 2020 | 10.1111/ggi.14051 | To investigate the relationships between multimorbidity, healthy aging and mortality. | Although it is not impossible for people with multimorbidity to age healthily, those with the most complex combination of diseases are at higher risk of death and have lower levels of healthy aging |
| Multimorbidity Patterns, Frailty, and Survival in Community-Dwelling Older Adults [63] | Nguyen QD, Wu C, Odden MC, Kim DH. | 2019 | 10.1093/gerona/gly205 | To investigate the importance of multimorbidity patterns in older adults with the same level of frailty phenotype | Considering both multimorbidity patterns and frailty is important for identifying older adults at greater risk of mortality. Of the 5 patterns identified, the neuropsychiatric class was associated with lower survival across all frailty levels. |
| Multiple Chronic Conditions and Hospitalizations Among Recipients of Long-Term Services and Supports [64] | Van Cleave, Janet H.; Egleston, Brian L.; Abbott, Katherine M.; Hirschman, Karen B.; Rao, Aditi; Naylor, Mary D. | 2016 | 10.1097/NNR.0000000000000185 | To determine the association between classes of mccs in newly enrolled LTSS recipients and the number of hospitalizations over a 1-year period following enrollment. | Older LTSS recipients with a combination of mccs that includes cardiopulmonary conditions have increased risk for hospitalization |
| Multiple chronic conditions: Implications for cognition - Findings from the Wisconsin Registry for Alzheimer's Prevention (WRAP) [65] | Bratzke LC, Carlson BA, Moon C, Brown RL, Koscik RL, Johnson SC. | 2018 | 10.1016/j.apnr.2018.06.004 | To examine the relationship between multiple chronic conditions (MCC) and cognitive decline. | Findings offer evidence of an association between specific MCC groups and the development of cognitive decline. Nurses should monitor and screen for cognitive decline in the presence of MCC to better target self-management interventions. |
| Patrones de multimorbilidad en adultos jóvenes en Cataluna: un análisis de clústeres [94] | Violán C, Foguet-Boreu Q, Roso-Llorach A, Rodriguez-Blanco T, Pons-Vigués M, Pujol-Ribera E, Valderas JM. | 2016 | 10.1016/j.aprim.2015.10.006 | To identify multimorbidity patterns in patients aged 19-44 years seen in primary care in Catalonia in 2010. Primary care in Catalonia in 2010. | Multimorbidity affects more than half of people between 19-44 years of age. The most prevalent cluster is formed by diagnoses that group common diseases (dental caries, common cold, smoking, anxiety disorders and back pain). Another pattern to highlight is cardiovascular-endocrine-metabolic in the 25-44 group. Knowledge of multimorbidity patterns in young adults would allow for a preventive preventive approach |
| Patterns and Consequences of Multimorbidity in the General Population: There is No Chronic Disease Management Without Rheumatic Disease Management [66] | Simões D, Araújo FA, Severo M, Monjardino T, Cruz I, Carmona L, Lucas R. | 2017 | 10.1002/acr.22996 | To identify empirical model-based patterns of multimorbidity from chronic noncommunicable diseases in the general population, with a focus on the contribution of rheumatic and musculoskeletal diseases (RMDS), and to quantify their association with adverse health outcomes. | Our study emphasizes RMDS as a major presence in multimorbidity in the general population. All multimorbidity patterns were associated with a wide set of adverse health outcomes. Management strategies for the patient with chronic cardiometabolic, respiratory or depressive conditions should also target RMDS |
| Patterns of Chronic Conditions and Their Associations With Behaviors and Quality of Life, 2010 [67] | Barile JP, Mitchell SA, Thompson WW, Zack MM, Reeve BB, Cella D, Smith AW. | 2015 | 10.5888/pcd12.150179 | To identify patterns of chronic conditions and to explore associations of latent class membership with sociodemographic characteristics, behavioral risk factors, and health. | Subgroups with distinct patterns of chronic conditions can provide direction for screening and surveillance, guideline development, and the delivery of complex care services |
| Patterns of chronic physical multimorbidity in psychiatric and general population [68] | Filipčić I, Šimunović Filipčić I, Grošić V, Bakija I, Šago D, Benjak T, Uglešić B, Bajić Ž, Sartorius N. | 2018 | 10.1016/j.jpsychores.2018.09.011 | To assess differences in the prevalence and patterns of self-reported chronic physical illness and multimorbidity in the general and psychiatric populations. | These findings indicate that mental disorders are associated with an increased risk of a wide range of chronic physical illnesses and multimorbidity. There is an urgent need for the development of the guidelines regarding the physical healthcare of all individuals with mental disorders with multimorbidity in focus. |
| Patterns of multimorbidity and demographic profile of latent classes in a Danish population-A register-based study [69] | Møller, Sanne Pagh; Laursen, Bjarne; Johannesen, Caroline Klint; Tolstrup, Janne S.; Schramm, Stine | 2020 | 10.1371/journal.pone.0237375 | The aim of the study was to identify and describe demographic characteristics of multimorbidity classes in three age groups (16–44 years, 45–64 years, and 65+ years). | The results of the study suggest that there are social inequalities in multimorbidity but that these inequalities are not universal to all types of multimorbidity. This supports that multimorbidity is diverse and should be prevented and treated accordingly. |
| Patterns of multi-morbidity and prediction of hospitalisation and all-cause mortality in advanced age [95] | Teh, Ruth O.; Menzies, Oliver H.; Connolly, Martin J.; Doughty, Rob N.; Wilkinson, Tim J.; Pillai, Avinesh; Lumley, Thomas; Ryan, Cristin; Rolleston, Anna; Broad, Joanna B.; Kerse, Ngaire | 2018 | 10.1093/ageing/afx184 | We aim to identify specific patterns of conditions of octogenarians living in NZ, contrasting two ethnic groups, and report associated prescribing appropriateness and subsequent hospitalizations and mortality over 4 years. | In octogenarians, hospitalization and mortality are better predicted by profiles of clusters of conditions rather than the presence or absence of a specific condition. Further research is required to determine if the cluster approach can be used to target patients to optimize resource allocation and improve outcomes. |
| Patterns of multimorbidity in 4588 older adults: Implications for a nongeriatrician specialist [110] | Piotrowicz, K., Pac, A., Skalska, A., Mossakowska, M., Chudek, J., Zdrojewski, T., Wiecek, A., Grodzicki, T., Gasowski, J. | 2021 | 10.20452/pamw.16128 | To assess the prevalence and patterns of multimorbidity in older subjects drawn from general population. | The age-specific analysis of clustering revealed differences in prevalence and patterns of comorbidities, which stresses the importance of individual approach to older patients. |
| Patterns of Multimorbidity in a Population-Based Cohort of Older People: Sociodemographic, Lifestyle, Clinical, and Functional Differences [96] | Marengoni, Alessandra; Roso-Llorach, Albert; Vetrano, Davide L.; Fernández-Bertolín, Sergio; Guisado-Clavero, Marina; Violán, Concepción; Calderón-Larrañaga, Amaia | 2020 | 10.1093/gerona/glz137 | To identify clusters of older people based on their multimorbidity patterns and to analyze differences among clusters according to sociodemographic, lifestyle, clinical, and functional characteristics. | In the present study, half of a cohort of older adults could be classified into five clinically meaningful clusters. These clusters showed significantly different sociodemographic, lifestyle, clinical, and functional profiles. This and similar approaches to the epidemiological study of multimorbidity are needed, not only to better understand the complex interactions among co-occurring diseases but also, even more importantly, to improve preventive interventions and optimally address individuals’ care needs and the risk of adverse outcomes. |
| Patterns of Multimorbidity in Adults: An Association Rules Analysis Using the Korea Health Panel [125] | Lee Y, Kim H, Jeong H, Noh Y. | 2020 | 10.3390/ijerph17082618 | To identify the prevalence and patterns of multimorbidity among Korean adults. | The results of the network analysis in four groups divided according to gender and age showed different characteristics for each group. Public health practitioners should adopt an integrated approach to manage multimorbidity rather than an individual disease-specific approach, along with different strategies according to age and gender groups. |
| Patterns of multimorbidity in the aged population. Results from the KORA-Age study [111] | Kirchberger I, Meisinger C, Heier M, Zimmermann AK, Thorand B, Autenrieth CS, Peters A, Ladwig KH, Döring A. | 2012 | 10.1371/journal.pone.0030556 | To explore patterns of comorbidity and multimorbidity in a Southern German population aged 65–94 years. | Our results confirmed the existence of co-occurrence of certain diseases in elderly persons, which is not caused by chance. Some of the identified patterns of multimorbidity and their overlap may indicate common underlying pathological mechanisms. |
| Patterns of patients with multiple chronic conditions in primary care: A cross-sectional study [70] | Tan XW, Xie Y, Lew JK, Lee PSS, Lee ES. | 2020 | 10.1371/journal.pone.0238353 | To identify the patterns of multimorbidity among a group of patients who visited primary care in Singapore | This study demonstrated that patients with multimorbidity in primary care could be classified into eight patterns. This knowledge could be useful for more precise management of these patients in the multiethnic Asian population of Singapore. Programs for early intervention for at-risk groups can be developed based on the findings. |
| Physical multimorbidity, depressive symptoms, and social participation in adults over 50 years of age: findings from the English Longitudinal Study of Ageing [71] | Ronaldson, A., Arias de la Torre, J., Bendayan, R., Yadegarfar, M.E., Rhead, R., Douiri, A., Armstrong, D., Hatch, S., Hotopf, M., Dregan, A. | 2021 | 10.1080/13607863.2021.2017847 | To identify specific patterns of physical multimorbidity and examine how these patterns associated with changes in social participation over time. | Physical multimorbidity reduced some aspects of social participation over time, with specific combinations of conditions having increased risk of reductions. |
| Prevalence and Patterns of Multimorbidity in a Nationally Representative Sample of Older Chinese: Results From the China Health and Retirement Longitudinal Study [97] | Yao, Shan-Shan; Cao, Gui-Ying; Han, Ling; Chen, Zi-Shuo; Huang, Zi-Ting; Gong, Ping; Hu, Yonghua; Xu, Beibei | 2020 | 10.1093/gerona/glz185 | To examine the prevalence of multimorbidity and explore its common patterns among a nationally representative sample of older Chinese | The prevalence and patterns of multimorbidity vary by gender and residential regions among older Chinese. Women and urban residents are more vulnerable to multimorbidity. Future studies are needed to understand the mechanisms underlying the identified multimorbidity patterns and their policy and interventional implications. |
| Prevalence and patterns of multimorbidity in Australian baby boomers: the Busselton healthy ageing study [72] | Hunter ML, Knuiman MW, Musk BAW, Hui J, Murray K, Beilby JP, Hillman DR, Hung J, Newton RU, Bucks RS, Straker L, Walsh JP, Zhu K, Bruce DG, Eikelboom RH, Davis TME, Mackey DA, James AL. | 2021 | 10.1186/s12889-021-11578-y | To assess the prevalence and patterns of multimorbidity using extensive individual phenotyping in a general population of Australian middle-aged adults. | Multimorbidity is common among middle-aged adults from a general population. Some conditions associated with ageing such as arthritis, bowel disease and depression-anxiety co-occur in clinically distinct patterns and at higher prevalence than expected by chance. These findings may inform further studies into shared biological and environmental causes of co-occurring conditions of ageing. Recognition of distinct patterns of multimorbidity may aid in a holistic approach to care management in individuals presenting with multiple chronic conditions, while also guiding health resource allocation in ageing populations. |
| Prevalence and Patterns of Multi-Morbidity in Serbian Adults: A Cross-Sectional Study [112] | Jovic D, Vukovic D, Marinkovic J. | 2016 | 10.1371/journal.pone.0148646 | To investigate the prevalence of multi-morbidity in the Serbia population and assessed the co-occurrence of chronic diseases by age and gender. | Multi-morbidity is a common occurrence among adults in Serbia, especially in the elderly. While several patterns may be explained by underlying pathophysiologies, some require further investigation and follow-up. Recognizing the complexity of multi-morbidity in Serbia is of great importance from both clinical and preventive perspectives given that it affects one-third of the population and may require adjustment of the healthcare system to address the needs of affected individuals. |
| Prevalence of multimorbidity in general practice: a cross-sectional study within the Swiss Sentinel Surveillance System (Sentinella) [136] | Excoffier S, Herzig L, N'Goran AA, Déruaz-Luyet A, Haller DM. | 2018 | 10.1136/bmjopen-2017-019616 | To estimate the prevalence of multimorbidity using a list of 75 chronic conditions derived from the International Classification for Primary Care, second edition and developed specifically to assess multimorbidity in primary care. Our aim was also to provide prevalence data for multimorbidity in primary care in a country in which general practitioners (gps) do not play a gatekeeping role in the health system. | In a country in which gps do not play a gatekeeping role within the health system, the prevalence of multimorbidity, as assessed using a list of chronic conditions specifically relevant to primary care, is high and increases with age. |
| Similar multimorbidity patterns in primary care patients from two European regions: results of a factor analysis [118] | Poblador-Plou B, van den Akker M, Vos R, Calderón-Larrañaga A, Metsemakers J, Prados-Torres A. | 2014 | 10.1371/journal.pone.0100375 | To compare the similarities among the multimorbidity patterns identified in primary care patients from two European regions (Spain and the Netherlands) with similar organizational features of their primary care systems, using validated methodologies. | The similarities found for the cardiometabolic, mechanical and psychiatric-substance abuse patterns in primary care patients from two different European countries could offer initial clues for the elaboration of clinical practice guidelines, if further evidenced in other contexts. This study also endorses the use of primary care electronic medical records for the epidemiologic characterization of multimorbidity. |
| Social Determinants and Health Behaviours among Older Adults Experiencing Multimorbidity Using the Canadian Longitudinal Study on Aging [132] | Wister, A. | 2021 | 10.1017/S0714980821000544 | To examine associations between lifestyle behavioral factors and appraisals of “healthy aging” among older adults experiencing multimorbidity. | The findings are examined using the SDHBM coupled with a resilience lens in order to elucidate how modifiable health behaviors can act as resources to mitigate multimorbidity adversities. This has implications for healthy aging for persons with multimorbidity, especially during the COVID-19 pandemic. |
| Social determinants of multimorbidity in Jamaica: application of latent class analysis in a cross-sectional study [73] | Craig, Leslie S.; Cunningham-Myrie, Colette A.; Hotchkiss, David R.; Hernandez, Julie H.; Gustat, Jeanette; Theall, Katherine P. | 2021 | 10.1186/s12889-021-11225-6 | To examine the social determinants of NCD multimorbidity in Jamaica, to better inform prevention and intervention strategies | This study provides a nuanced understanding of the social patterning of multimorbidity in Jamaica, identifying biological, health system, and structural determinants as key factors associated with specific multimorbidity profiles. Future research using longitudinal designs would aid understanding of disease trajectories and clarify the role of SDH in mitigating risk of accumulation of diseases. |
| Soft clustering using real-world data for the identification of multimorbidity patterns in an elderly population: cross-sectional study in a Mediterranean population [98] | Violán, Concepción; Foguet-Boreu, Quintí; Fernández-Bertolín, Sergio; Guisado-Clavero, Marina; Cabrera-Bean, Margarita; Formiga, Francesc; Valderas, Jose Maria; Roso-Llorach, Albert | 2019 | 10.1136/bmjope n-2019-029594 | To identify, with soft clustering methods, multimorbidity patterns in the electronic health records of a population ≥65 years, and to analyze such patterns in accordance with the different prevalence cut-off points applied analysis allows individuals to be linked simultaneously to multiple clusters and is more consistent with clinical experience than other approaches frequently found in the literature. | Multimorbidity patterns were obtained using fuzzy c-means cluster analysis. They are clinically meaningful clusters which support the development of tailored approaches to multimorbidity management and further research. |
| Spreading of diseases through comorbidity networks across life and gender [126] | Chmiel, A., Klimek, P., Thurner, S. | 2014 | 10.1088/1367-2630/16/11/115013 | To propose a specific phenomenological comorbidity network of human diseases that is based on medical claims data of the entire population of Austria | For the first time we are able to show that patients predominantly develop diseases that are in close network proximity to disorders that they already suffer. The model explains more than 85% of the variance of all disease incidents in the population. The presented methodology could be of importance for anticipating age-dependent disease profiles for entire populations, and for design and validation of prevention strategies. |
| Survival in relation to multimorbidity patterns in older adults in primary care in Barcelona, Spain (2010-2014): a longitudinal study based on electronic health records [99] | Ibarra-Castillo C, Guisado-Clavero M, Violan-Fors C, Pons-Vigués M, López-Jiménez T, Roso-Llorach A; Collaborators. | 2018 | 10.1136/jech-2017-209984 | To compare survival across older adults with different chronic multimorbidity patterns (cmps) | Mortality and survival vary according to cmps in older adults stratified by sex and age. Our findings are useful for guiding the design and implementation of clinical management strategies |
| The burden of cardiovascular morbidity in a European Mediterranean population with multimorbidity: a cross-sectional study [134] | Violán C, Bejarano-Rivera N, Foguet-Boreu Q, Roso Llorach A, Pons-Vigués M, Martin Mateo M, Pujol-Ribera E. | 2016 | 10.1186/s12875-016-0546-4 | To identify and describe the cardiovascular diseases among the patients with multimorbidity | More than 50 % percent of patients with multimorbidity had cardiovascular diseases, the most frequent being hypertension. The presence of cardiovascular risk factors and the cardiovascular risk profile were higher in the multimorbidity group than the non-multimorbidity group. Hypertension, diabetes and dyslipidemia constituted the most prevalent multimorbidity pattern. |
| The epidemiology of multimorbidity in primary care: a retrospective cohort study [131] | Cassell, Anna; Edwards, Duncan; Harshfield, Amelia; Rhodes, Kirsty; Brimicombe, James; Payne, Rupert; Griffin, Simon | 2018 | 10.3399/bjgp18X695465 | To describe the epidemiology of multimorbidity in adults in England, and quantify associations between multimorbidity and health service utilization | This study found that multimorbidity was associated with female sex, increased age, and lower SES. Physical–mental comorbidity made up a substantial proportion of all patients with multimorbidity (33.8%). The proportion of patients with multimorbidity who have a physical–mental comorbidity is higher among females, younger age groups and groups with greater levels of socioeconomic deprivation. Additionally, multimorbidity was highly associated with increased rates of GP consultations, prescriptions, and hospitalizations, which highlights the disproportionately large demand that patients with multimorbidity place on the UK’s overburdened healthcare system. |
| The influence of age, gender and socio-economic status on multimorbidity patterns in primary care. First results from the multicare cohort study [113] | Schäfer, Ingmar; Hansen, Heike; Schön, Gerhard; Höfels, Susanne; Altiner, Attila; Dahlhaus, Anne; Gensichen, Jochen; Riedel-Heller, Steffi; Weyerer, Siegfried; Blank, Wolfgang A.; König, Hans-Helmut; von dem Knesebeck, Olaf; Wegscheider, Karl; Scherer, Martin; van den Bussche, Hendrik; Wiese, Birgitt | 2012 | 10.1186/1472-6963-12-89 | To analyze the association of socio-demographic variables, and especially socio-economic status with multimorbidity in general and with each multimorbidity pattern | Our study confirms that the morbidity load of multimorbid patients is associated with age, gender and the socioeconomic status of the patients, but there were no effects of living arrangements and marital status. We could also show that the influence of patient characteristics is dependent on the multimorbidity pattern concerned, i.e. There seem to be at least two types of elderly multimorbid patients. First, there are patients with mainly cardiovascular and metabolic disorders, who are more often male, have an older age and a lower socioeconomic status. Second, there are patients mainly with ads and pain-related morbidity, who are more often female and equally distributed across age and socio-economic groups. |
| The modeling of internalizing disorders on the basis of patterns of lifetime comorbidity: associations with psychosocial functioning and psychiatric disorders among first-degree relatives [115] | Seeley JR, Kosty DB, Farmer RF, Lewinsohn PM. | 2011 | 10.1037/a0022621 | To comparatively evaluate three competing and theoretically plausible measurement models of putative internalizing disorders based on the patterns of lifetime comorbidity, to evaluate the extent to which viable measurement models are associated with the density of specific psychiatric disorders among first-degree relatives of probands, and to investigate the extent to which viable models of lifetime disorder comorbidity account for psychosocial functioning among probands at age 30. | Symptoms that have specific or relatively unique associations with depressed mood include sadness, loss of interest or pleasure, hypersomnia, suicidal ideation, and helplessness. Symptoms relatively specific to anxiety include apprehension and those linked to autonomic activation tied to the fight/flight/freeze response, such as symptoms common to episodes of panic (e.g., heart palpitations, rapid and shallow breathing, trembling, muscle tension; additional research might also seek to establish whether examples of diagnostic comorbidity observed here and elsewhere indicate problems with discriminant validity of dsm disorder criterion sets whereby two or more disorder labels are invoked to account for the same underlying process or phenomenology. |
| The patterns of Non-communicable disease Multimorbidity in Iran: A Multilevel Analysis [74] | Khorrami Z, Rezapour M, Etemad K, Yarahmadi S, Khodakarim S, Mahdavi Hezaveh A, Kameli M, Khanjani N. | 2020 | 10.1038/s41598-020-59668-y | To investigate the patterns of non-communicable disease multimorbidity and their risk factors at the individual and aggregated level. | The findings of this study showed that behavioral factors such as physical inactivity, tobacco use and urbanization are associated with multimorbidity in iran; and there is a need to allocate resources for controlling, preventing, and managing these risk factors. Prevention and control of multimorbidity requires health promotion programs that increase public awareness about modifiable risk factors, particularly among the at-risk populations. A deeper understanding of these patterns may lead to the development of preventive measures to reduce the burden of these diseases and offer new and comprehensive ways to manage these common conditions. |
| Trends of multimorbidity in 15 European countries: a population-based study in community-dwelling adults aged 50 and over [135] | Souza DLB, Oliveras-Fabregas A, Minobes-Molina E, de Camargo Cancela M, Galbany-Estragués P, Jerez-Roig J. | 2021 | 10.1186/s12889-020-10084-x | To analyse the prevalence trends of multimorbidity among European community-dwelling adults. | This information can be useful for policy makers when planning health promotion and prevention policies addressing modifiable risk factors in health. |
| Use of latent class analysis to identify multimorbidity patterns and associated factors in Korean adults aged 50 years and older [75] | Park, Bomi; Lee, Hye Ah; Park, Hyesook | 2019 | 10.1371/journal.pone.0216259 | To identify multimorbidity patterns in the general Korean population aged over 50 years using nationally representative survey data; and to explore whether such patterns were associated with certain sociodemographic characteristics and quality-of-life | The co-occurrence of chronic diseases was not attributable to chance. Multimorbidity patterns were associated with sociodemographic factors and quality-of-life. Our results suggest that targeted, integrated public health and clinical strategies dealing with chronic diseases should be based on an understanding of multimorbidity patterns; this would improve the quality-of-life of vulnerable multimorbid adults. |
| Using item response theory with health system data to identify latent groups of patients with multiple health conditions [76] | Prenovost, Katherine M.; Fihn, Stephan D.; Maciejewski, Matthew L.; Nelson, Karin; Vijan, Sandeep; Rosland, Ann-Marie | 2018 | 10.1371/journal.pone.0206915 | To introduce a novel application of IRT modeling using accessible medical data to present potentially clinically useful information on subgroups of high-risk patients | By applying MD-IRT models to data already existing in modern healthcare systems, we were able to identify diagnostic constellations among otherwise undifferentiated high-risk patients, thus efficiently grouping patients into clinically distinct subgroups with unique markers of the degree of complexity within each group. If validated, care management approaches tailored to each group’s needs and markers of complexity may more efficiently and effectively reach complex patients at risk for poor health outcomes. |
| Latent classes of posttraumatic psychiatric comorbidity in the general population [138] | Anthony J. Rosellini, Péter Szentkúti, Erzsébet Horváth-Puhó, Meghan L. Smith, Isaac Galatzer-Levy, Timothy L. Lash, Sandro Galea, Paula P. Schnurr, Henrik T. Sorensen, and Jaimie L. Gradusb, | 2021 | 10.1016/j.jpsychires.2021.02.013 | To identify and characterize novel latent classes of posttraumatic psychiatric comorbidity in this cohort. | Our prior study found elevated rates of all mental disorder categories in this trauma cohort compared to individuals who experienced a family member’s non-suicide death (Gradus et al., under review). In the current study, all classes were characterized by having more diagnoses in the five years following the traumatic event than in the entire period before. Although these findings suggest that our registry-defined traumatic events are associated with subsequent psychopathology, firm causal conclusions cannot be made. Some individuals had at least one similar diagnosis prior to the traumatic event. Further, the number of pre-event diagnoses likely was underestimated because information about psychiatric diagnoses before 1994 was unavailable (during the ICD-8 classification period). At the same time, having access to any longitudinal data on pre-event diagnoses is a strength; the literature has relied heavily on retrospective self-reports of both traumatic events and psychopathology. |
