# Supplementary material for: Social determinants of multimorbidity patterns: A systematic review
Source: Front Public Health. 2023 Mar 27;11:1081518. doi: 10.3389/fpubh.2023.1081518 (PMC10084932; doi:10.3389/fpubh.2023.1081518)
Supplement: Supplementary file 4 [file Table_4.DOCX]

Supplementary Material

# Table 4: Pattern extraction methods and results

| TITLE | METHOD OF OBTAINING PATTERNS | PATTERNS | INEQUALITIES/ FACTORS | POPULATION CHARACTERISTICS | DATA SOURCE AND SAMPLE SIZE |
| --- | --- | --- | --- | --- | --- |
| A cluster-based approach for integrating clinical management of Medicare beneficiaries with multiple chronic conditions [78] | Hierarchical cluster analysis (HCA) | Cluster CHF: congestive heart failure.  Cluster CKD: chronic kidney disease.  Cluster Neuro: neurological conditions.  Cluster DM: diabetes.  Cluster CA: cancer.  Cluster CPD: chronic pulmonary disease.  Cluster CVD: cardiovascular disease.  Cluster BH: behavioral health.  Cluster Obes: obesity.  Cluster OA: osteoarthritis.  Cluster HTN: hypertension.  Cluster HLP: lipid metabolism disorders.  Cluster Others. | Age, ethnic, sex, BMI and number of outpatients visits. | Patients < 65 years old were included since individuals in this age group who receive Social Security Disability Insurance benefits qualify for Medicare benefits.  Country: USA | Data from the Electronic Health Records Systems (EHRS) were obtained from patient encounters in our Accountable Care Organizations (ACO) and linked with the Medicare Shared Sav-ings Program (MSSP). The sample size was 44,645 patients. |
| A Latent Class Analysis of Multimorbidity and the Relationship to Socio-Demographic Factors and Health-Related Quality of Life. A National Population-Based Study of 162,283 Danish Adults. [41] | Latent class analysis | Relatively Healthy.  Hypertension.  Musculo- skeletal Disorders .  Headache-Mental Disorders.  Asthma-Allergy.  Complex Cardiometabolic Disorders.  Complex Respiratory Disorders. | Gender, age, educational level,  Cohabitation status, ethnic origin and work status. | Individuals with residence in Denmark aged 16 years and over.  Country: Denmark | Analyses in this study are based on data from the Danish national health survey coined “How are you? is a national, representative, cross-sectional survey with 162,283 individuals. |
| Age density patterns in patients medical conditions: A clustering approach. [79] | Hierarchical cluster analysis (HCA) | A (Suppurative otitis media and unspecified, other viral infections characterized by skin lesions. Other acquired deformities of limbs, otitis media nonsuppurative, specific developmental disorders of speech and language, chronic diseases of tonsils and adenoids, hyperkinetic disorders, acute bronchiolitis). B (amebiasis, acute tonsillitis, allergic rhinitis, hives, other non-infectious gastroenteritis and colitis, diarrhea and gastroenteritis of presumed infectious origin, candidiasis, acute infections of the upper airways of multiple and unspecified sites, viral conjunctivitis, omphalocele, bronchiolitis not specified as acute or chronic, asthma, other respiratory disorders). C (only caesarean delivery, neonatal jaundice due to other causes and unspecified, miscarriage, false labor, maternal care for other conditions predominantly related to pregnancy, excessive vomiting in pregnancy ). D (disorders of refraction and accommodation, acute sinusitis, other disorders of the nose and paranasal sinuses, acne, obesity, leiomyoma of the uterus, menstruation excessive, absent menstruation). E (gastroesophageal reflux disease, other joint disorders not elsewhere classified, back pain, other enthesopathies, shoulder injury, menopausal disorders and perimenopause, benign neoplasm of other and ill-defined parts of digestive system, prostate hyperplasia). F (other disorders of the urinary tract, essential hypertension (primary), senile cataract, angina pectoris, pneumonia, heart failure, other cardiac arrhythmias, other malignant neoplasms of the skin). | Sex and age. | Patients in Brazil.  Country: Brazil | Study a heterogeneous sample of 1.7 million patients in Brazil during a period of 17 months. |
| Analysis of multimorbidity networks associated with different factors in Northeast China: a cross-sectional analysis [119] | Network nodes | Related models | Sex, age, and hospitalisation duration. | Hospitals in Jilin Province, China.  Country: China | Analysed data obtained from the hospital information systems or electronic medical record systems of 15 general hospitals in Jilin Province. This cross-sectional analysis included 431295 inpatients. |
| Bayesian networks to identify potential high-risk multimorbidity and intervention clusters in inpatients: an explorative data mining study [120] | Bayesian networks | Related models | Hospital admission, emergency admission status, sex, and age. | Historical cohort study at the University Hospital Basel, an 850-bed tertiary care hospital in Switzerland.  Country: Switzerland | Performed an explorative, historical cohort study of 190,837 consecutive inpatient cases at a tertiary care centre with an integrated platform for routine healthcare data in Switzerland. From January 2012 through to December 2017. |
| Burden of multimorbidity in relation to age, gender and immigrant status: a cross-sectional study based on administrative data [116] | Factor analysis | Pattern 1:Psychiatric disorders.  Pattern 2 :cardiovascular, renal, pulmonary and cerebrovascular diseases.  Pattern 3: Neurological diseases.  Pattern 4:Liver diseases, AIDS/HIV and substance use.  Pattern 5: Tumours. | Age, sex and non-Italian citizenship. | All adults residing in Emilia-Romagna.  Country: Italy | Cross-sectional study based on demographic and clinical data retrieved from five data sources linked using the patient’s identification code: the hospital discharge record, the mental health information system, residential mental health-care discharge records, the outpatient pharmaceutical database and the regional mortality register database. The study population included 622 026 men and 751 011women. |
| Characteristics, service use and mortality of clusters of multimorbid patients in England: a population-based study [42] | Latent class analysis | **18-44 years**:  Depression, Anxiety and pain.  Pain, Hearing loss and hypertension.  Asthma, IBS and depression.  IBS, Depression and hearing loss.  PSM, Alcohol and depression.  **45-64 years:**  Hypertension, diabetes and pain.  IBS, hearing loss and pain.  Depression, pain and anxiety.  Asthma, pain and COPD.  Alcohol, PSM and pain  **65-84 years:**  Hypertension, diabetes and pain  Depression, pain and anxiety  CHD, diabetes and atrial fibrillation.  COPD, asthma and pain.  Pain, CHD and depression.  **85+ years:**  Hypertension, hearing loss, diabetes.  Pain, depression and constipation.  CHD, atrial fibrillation and heart failure.  Asthma, COPD and pain. | Age, sex, deprivation and smoke. | Patients aged 18 years and above.  Country: UK | Our analysis used the Clinical Practice Research Datalink (CPRD)-GOLD database where anonymised and longitudinal primary care clinical data are contributed by UK general (family) practices. The study included a representative set of  multimorbid adults (N = 113,211) |
| Chronic condition patterns in the US population and their association with health related quality of life [43] | Latent class analysis | Healthy. Vascular risk. Anxiety group. Heart disease. Severely-impaired | Age, gender, race, education, health insurance, marital status, income to poverty threshold ratio, smoke and BMI. | Participants aged 18 years and older of the U.S.  Country: USA | This cross-sectional study included data from the 2010 to 2015 MEPS. MEPS is a source of data on the cost and usage of healthcare in the US. The study sample is composed of 86,745 individuals |
| Clustering of 27,525,663 death records from the united states based on health conditions associated with death: An example of big health data exploration [127] | Self-organizing maps | Open wound/suffocation. Other physical harm. Neuro-degenerative disease. Other cancer. Lung cancer. Respiratory disease. Stroke. Gastro-intestinal cancer. Other geriatric disease. Urological/gastrointestinal disease/other infection. Other cardiovascular disease. Liver disease. Poisoning. Myocardial. Atherosclerotic heart disease and chronic kidney disease | Age, gender, race, education, resident status, and marital status. | Multiple Cause-of-Death Mortality Data from 2006 to 2016 of the National Vital Statistics System of the National Center for Health Statistics, which provides data obtained from death certificates in the US.  Country: USA | Data from 27,525,663 deceased people in the United States registered between 2006 and 2016 from the National Vital Statistics System of the National Center for Health Statistics were analyzed. |
| Clustering of Mental and Physical Comorbidity and the Risk of Frailty in Patients Aged 60 Years or More in Primary Care. [80] | Cluster analysis with K-means | Cluster 1, 2 and 3 | Age and BMI. | In patients ≥60years in a primary healthcare setting in Osijek (eastern Croatia).  Country: Croatia | A cross-sectional retrospective study of 159 patients included in the cluster analysis |
| Clusters of Multiple Complex Chronic Conditions: A Latent Class Analysis of Children at End of Life [44] | Latent class analysis | Neurological.  Cancer.  Cardiovascular | Sex, race/ethnicity, age, private insurance and medicaid eligibility. | Pediatric California Medicaid beneficiary.  Country: USA | The study was designed as a pooled cross-sectional with 1423 pediatric participants from the 2007 to 2008 California Medicaid data. |
| Comorbidity patterns and socioeconomic inequalities in children under 15 with medical complexity: a population-based study [45] | Latent class analysis | Oncology class.  Congenital  Perinatal class. Neurodevelopment class. Respiratory class | Age, socioeconomic position and sex. | Population of Catalonia (Spain). Catalonia under the age of 15.  Country: Spain | Two main sources of data were used: the central registry of insured persons was used to obtain the reference population and the Catalan Health Surveillance System database. It is a cross-sectional study with 3480 boys and 2470 girls children with medical complexity |
| Comorbidity profile of mental disorders among adolescents: A latent class analysis [46] | Latent class analysis | Comorbid emotional disorders class.  Comorbid behavioural disorders class. | Age, gender, race/ethnicity, parents’ education level, household income, urbanicity and biological parents living with adolescent. | Adolescents between 13 and 18 years of age in the United States.  Country: USA | The present study used data from the National Comorbidity Survey Adolescent Supplement (NCS-9), a cross- sectional study |
| Comorbidity Profiles Identified in Older Primary Care Patients Who Attempt Suicide [47] | Latent class analysis | Minimal comorbidity.  Chronic pain-osteoarthritis. Depression-chronic pain. Depression-medical comorbidity. | Age mean, sex, race, education, median income tertile and marital status. | 65 years and older who used Veterans Affairs health care services and had a first late-life suicide attempt between 2012 and 2014.  Country: USA | Cohort study with 2,131 patients of Veterans Affairs (VA) medical centers in the United States. |
| Comparative analysis of methods for identifying multimorbidity patterns: a study of 'real-world' data [81] | Hierarchical cluster analysis (HCA) and exploratory factor analysis (EFA) | **Women**  Cluster 1: other dorsopathies; neurotic, stress-related and somatoform disorders; other soft-tissue disorders; non-inflammatory disorders of female genital tract; other joint disorders; diseases of veins, lymphatic vessels and lymph nodes, not elsewhere classified; benign neoplasms; arthrosis.  Cluster 2: metabolic disorders; hypertensive diseases; obesity and other hyperalimentation; diabetes mellitus.  Cluster 3: mental and behavioural disorders due to psychoactive substance use; disorders of thyroid gland; mood (affective) disorders; disorders of bone density and structure.  Cluster 4: acute upper respiratory infections; diseases of oral cavity, salivary glands and jaws; dermatitis and eczema; mycoses.  **Men**  Cluster 1:  metabolic disorders; mental and behavioural disorders due to psychoactive substance use; hypertensive diseases; other dorsopathies; obesity and other hyperalimentation; diabetes mellitus.  Cluster 2: other soft tissue disorders; diseases of male genital organs; other joint disorders; diseases of esophagus, stomach and duodenum; diseases of veins, lymphatic vessels and lymph nodes, not elsewhere classified; hernia; benign neoplasms; arthrosis; other diseases of intestines.  Cluster 3: neurotic, stress-related and somatoform disorders; diseases of oral cavity, salivary glands and jaws; chronic lower respiratory diseases; acute upper respiratory infections; other diseases of upper respiratory tract; dermatitis and eczema; episodic and paroxysmal disorders; behavioural syndromes associated with physiological disturbances and physical factors; mood (affective) disorders; mycoses.  Cluster 4: other disorders of ear; visual disturbances and blindness; viral infections characterised by skin and mucous membrane lesions; disorders of skin appendages; disorders of conjunctiva; disorders of ocular muscles, binocular movement, accommodation and refraction; other disorders of the skin and subcutaneous tissue; infections of the skin and subcutaneous tissue; disorders of eyelid, lacrimal system and orbit. | Sex and age. | Individuals aged 45–64 years in 274 primary health care teams from 2010 in Catalonia (Spain).  Country: Spain | Cross-sectional study. Electronic health records for 408994 patients with multimorbidity. |
| Comparing Multimorbidity Patterns Among Discharged Middle-Aged and Older Inpatients Between Hong Kong and Zurich: A Hierarchical Agglomerative Clustering Analysis of Routine Hospital Records [82] | Hierarchical cluster analysis (HCA) | **Hong Kong**  Cluster H1: Cancer, non-metastatic, cirrhosis, diabetes, parkinson’s disease, peptic ulcer disease, severe constipation, heart failure and hypertension.  Cluster H2: Chronic pain, heart failure and hypertension.  Cluster H3: Atrial fibrillation, chronic kidney disease and myocardial infarction and hypertension,  Cluster H4: Heart failure, hypertension and chronic pain.  Cluster H5: Heart failure, hypertension and stroke.  Cluster H6: Cancer, metastatic, peripheral vascular disease, chronic pain, heart failure and hypertension.  Cluster H7: Alcohol misuse, asthma, dementia, depression, epilepsy, hepatitis B, hypothyroidism, inflammatory bowel disease, psoriasis, rheumatoid arthritis, schizophrenia, multiple sclerosis and heart failure and hypertension.  Cluster H8: Psoriasis, heart failure, hypertension and stroke.  Cluster H9: Cancer, lymphoma, stroke, heart failure and hypertension.  **Zurich**  Cluster Z1: Diabetes, parkinson’s disease, heart failure, hypertension and chronic pain.  Cluster Z2: Cancer, non-metastatic, peripheral vascular disease, heart failure and hypertension.  Cluster Z3: Atrial fibrillation. Chronic kidney disease, chronic pain, hypertension.  Cluster Z4: Cancer lymphoma, cancer metastatic, cirrhosis, depression, hepatitis B, inflammatory bowel disease, rheumatoid arthritis, schizophrenia, severe constipation and hypertension.  Cluster Z5. Hypothyroidism, epilepsy and hypertension.  Cluster Z6: Dementia, heart failure, psoriasis, chronic pain and hypertension.  Cluster Z7: Alcohol misuse, asthma, chronic pulmonary disease, epilepsy, inflammatory bowel disease, irritable bowel syndrome, multiple sclerosis and hypertension.  Cluster Z8: Hypertension, stroke, peptic ulcer disease and hypertension.  Cluster Z9: Myocardial infarction. Chronic kidney disease and hypertension. | Sex, age and country. | Patients aged ≥ 45 from all public hospitals in Hong Kong and from the University Hospital Zurich.  Countries: Switzerland and Japan | Retrospective analysis of clinical records of discharged patients from all public hospitals in Hong Kong during January 2010-December 2013 and from the University Hospital Zurich during August 2009-August 2017. |
| Comparisons of disease cluster patterns, prevalence and health factors in the USA, Canada, England and Ireland [48] | Latent Class Analysis | **Usa:**  Group 1:high probability of disease. Group 2:metabolic, cardiovascular, arthritis,  Cancer. Group 3:osteoporosis,arthritis, hypertension, psychological, cancer. Group 4:metabolic, arthritis, psychological, cancer  **Canada:**  Group 1:high probability of disease. Group 2:metabolic, arthritis, cancer”. Group 3:osteoporosis,arthritis, hypertension,psychological, cancer (female). Group 4:metabolic, arthritis, país, edad, psychological  **England:**  Group 1:high probability of disease. Group 2:metabolic, arthritis. Group 3:osteoporosis, arthritis, hypertension, psychological (female).Group 1:high probability of disease. Group 2:metabolic, cardiovascular. Group 3:osteoporosis,arthritis, hypertension (female).  Group 4:metabolic, arthritis.  **Ireland:**  Group 1:High Probability of Disease.  Group 2:Metabolic, Cardiovascular.  Group 3: Osteoporosis, Arthritis, Hypertension (Female).  Group 4:Metabolic, Arthritis. | Sex, education, income, smoke, BMI, alcohol and employment. | White and non-Hispanic people aged between 52–85 of the U.S., Canada, UK and Ireland.  Countries:USA, Canada, UK and Ireland. | Participants (n = 62,111) were drawn from the US Health and Retirement Study (n = 10,858); the Canadian Longitudinal Study on Ageing (n = 36,647); the English Longitudinal Study of Ageing (n = 7938) and The Irish Longitudinal Study on Ageing (n = 6668). |
| Complex comorbidity clusters in OEF/OIF veterans: the polytrauma clinical triad and beyond [49] | Latent class analysis | Cluster 1: Polytrauma clinical triad+ depression chronic disease.  Cluster 2: Polytrauma clinical triad.  Cluster 3: mental health, substance abuse.  Cluster 4: Sleep, amputation, chronic disease.  Cluster 5: Pain, moderate PTSD.  Cluster 6: Relatively healthy. | Age, sex, race/ethnicity, marital status and national guard/reserve. | Veterans from Afghanistan and Iraq (Operations Enduring and Iraqi Freedom, and Operation New Dawn).  Country: USA | Data for Fiscal Years 2008 to 2010 from the Veterans Health Administration Decision Support System National Data Extracts, the OEF/OIF roster, and the VA Vital Status file. The sample size was 191,797. |
| Contextual and individual inequalities of multimorbidity in Brazilian adults: a cross-sectional national-based study. [103] | Factor analysis (FA) | Factor 1: cardiometabolic.  Factor 2 (respiratory/mental/ muscle-skeletal). | Sex, age, skin colour, marital status, schooling in years, wealth index, private health plan, geographical area (urban/rural), state-level- education and state-level- income. | People aged 18 or over living in permanent housing, located in urban or rural areas, covering the country’s five major geographical regions, its 26 states and Federal District.  Country: Brazil | A national-based cross-sectional study was carried out in 2013. The sample comprised 60 202 individuals. |
| Deconstructing Complex Multimorbidity in the Very Old: Findings from the Newcastle 85+ Study [83] | Hierarchical cluster analysis (HCA) | Cluster A: Hypertention, heart failure, atrial fibrillation or flutter, cerebrovascular disease, peripheral vascular disease, asthma, thyroid disease, diabetes melluitusm cancer within 5 years and renal impairment.  Cluster B: Atrial fibrillation or flutter, inflammatory arthritis, thyroid disease, diabetes mellitus, urinary incontinence, visual impairment, hearing impairment, falls, and cognitive impairment.  Cluster C: ischemic heart disease, inflammatory arthritis, thyroid disease, diabetes mellitus and hearing impairment.  Cluster D: osteoarthritis, chronic obstructive pulmonary disease, asthma, urinary incontinence, falls and cognitive impairment.  Cluster E: atrial fibrillation or flutter, osteoporosis, visual impairment, hearing impairment and cognitive impairment  (higher than average prevalence of a condition defined as a ratio of prevalence in cluster). | Sex. | Members of the 1921 birth cohort living in Newcastle upon Tyne or North Tyneside (North East England) were recruited at around age 85.  Country: UK | Recruitment and baseline assessment took place over a 17-month period in 2006-2007. Data ascertained from general practice records and participant assessment. |
| Description of multimorbidity clusters of admitted patients in medical departments of a general hospital [84] | Multiple correspondence analysis (MCA) and expert knowledge | Cluster 1: alcoholic liver disease, alcoholic dependency syndrome and digestive tract and lung malignant neoplasms associated to a<50 age category.  Cluster 2: large intestine malignant neoplasm, lymphoma, myeloma, prostate malignant neoplasm, breast malignant neoplasm and other malignant neoplasms.  Cluster 3: malnutrition, Parkinson disease and other motility disorders, dementia and other mental disorders associated with a>80 age category.  Cluster 4: atrial fibrillation/flutter, cardiac failure, chronic kidney failure, and heart valve disease, more related to female gender and 71–80 age category.  Cluster 5: hypertension/hypertensive cardiomyopathy, type 2 diabetes mellitus, dyslipidaemia, ischaemic cardiomyopathy, obesity, and sleep apnoea, associated with a male gender and 61–70 to 71–80 age categories. | Gender, age and number of hospitalisations (stay time in days). | All patients discharged from all services of the Medic Area of the Lucus Augusti Hospital (Lugo, Spain).  Country: Spain | Data from the CMBD  (Conjunto mínimo básico de datos - Minimum Basic Hospital Data Set (MBDS)) of the Lucus Augusti Hospital (Spain), hospitalisations and patients, realising a retrospective cohort study among the 74220 patients Area between 01 January 2000 and 31 December 2015. |
| Differences in Clinical Outcomes of Adults Referred to a Homeless Transitional Care Program Based on Multimorbid Health Profiles: A Latent Class Analysis [50] | Latent class analysis | Low comorbidity.  High comorbidity.  High tri-morbidity. High alcohol use.  High medical illness. | Sex, age, race and insurance status. | Adults referred to Durham Homeless Care Transitions (DHCT).  Country: UK | Administrative data from a health system electronic health record for adults referred to the Durham Homeless Care Transitions program from July 2016 to June 2020. 497 patients were included. |
| Differences in psychiatric comorbidities and gender distribution among three clusters of personality disorders: A nationwide population-based study [133] | Expert knowledge | Cluster A: paranoid, schizoid, and schizotypal.  Cluster B: antisocial, borderline, histrionic, and narcissistic.  Cluster C: avoidant, dependent, and obsessive–compulsive. | Gender and age. | Patients with a diagnosis of the clusters. Taiwanese people.  Country: Taiwan | This study used the Taiwan national database between 1995 and 2013. 9845 cases were included. |
| Disability, quality of life and all-cause mortality in older Mexican adults: association with multimorbidity and frailty [114] | Principal-components analysis | Pattern 1: Cardiopulmonary.  Pattern 2: Vascular-metabolic.  Pattern 3: Mental-musculoskeletal. | Age (additional file). | Mexican adults aged 50 years and older and a smaller comparison group subjects aged 18– 49 years.  Country: Mexico | Analytical samples included 1410 respondents for disability and quality of life, and 1792 for mortality. This study performed a longitudinal analysis. |
| Effect of Multimorbidity on Health-Related Quality of Life in Adults Aged 55 Years or Older: Results from the SU.VI.MAX 2 Cohort [104] | Exploratory factor analysis | **Pattern A:** Respiratory impairments, ear, nose and throat impairments, digestive impairments, vertebral diseases, osteoporosis, arthritis and rheumatism, thyroid disease, anxiety/depression, sleeping troubles and memory impairments.  **Pattern B:** Hypertension, heart failure, arrhythmias and palpitations, ischemic cardiovascular impairments, respiratory impairments, adenoma or prostatic hyperplasia and diabetes. | Sex and age. | Older than 55 years at enrollment and received a geriatric assessment.  Country: France | It is an additional observational follow-up study. Data for 5,647 participants included in the SUpplementation en VItamines et Minéraux AntioXydants 2 (SU.VI.MAX 2) population-based trial. |
| Ethnic and geographic variations in multimorbidty: Evidence from three large cohorts [51] | Latent class analysis | **Charlson comorbidity**  LC1: ‘Healthier Patients’ with lower probabilities for most of the comorbidities.  LC2: ‘Diabetics’ with high level of renal disease.  LC3: ‘COPD and malignancy’  with high levels of renal disease. CHF, vascular and cerbrovascualr diseases.  **Elixhauser comorbidity index**. LC1: ‘Healthier Patients’ with higher probabilities for uncomplicated hypertension.  LC2: ‘Hypertension, COPD and arrhythmia’ with  high levels of fluid electrolyte disorder, renal disease, CHF, depression, vascular and cerebrovascular diseases.  LC3: ‘Diabetics’ with higher probabilities for uncomplicated hypertension, obesity and depression. | Race/ethnicity and geography, age, gender, marital status, service connected, homeless-status, and VA-region. | Veterans VHA National- Patient-Care, Pharmacy- Benefits-Management, and Vital-Status data-bases.  Country: USA | This study selected Veterans with diabetes mellitus (n = 1,263,906), chronic kidney disease (n = 2,190,564), and traumatic brain injury (n = 167,954) to cover a wide period of time from 1998 to 2010. |
| Ethnicity and psychiatric comorbidity in a national sample: evidence for latent comorbidity factor invariance and connections with disorder prevalence [102] | Factor analysis | Internalizing: major depressive disorder, dysthymic disorder, generalized anxiety disorder, panic disorder, social phobia and specific phobia.  Externalizing: antisocial personality disorder, nicotine dependence, alcohol dependence, marijuana dependence and other drug dependence. | Ethnic. | Non-institutionalized United States population at least 18 years of age.  Country: USA | D43,093 individuals who participated in the NESARC (2001-2002). Lifetime and 12-month DSM-IV diagnoses were made using the Alcohol Use Disorder and Associated Disabilities Interview Schedule—DSM-IV Version. |
| Examining health disparities by gender: A multimorbidity network analysis of electronic medical record [121] | Network nodes | Infectious and parasitic diseases.  Neoplasms.  Endocrine, nutritional and metabolic diseases, and immunity disorders.  Diseases of the blood and blood-forming organs.  Mental disorders.  Diseases of the nervous system.  Diseases of the sense organs.  Diseases of the circulatory system.  Diseases of the respiratory system.  Diseases of the digestive system.  Diseases of the genitourinary system.  Complications of pregnancy, childbirth, and the puerperium.  Diseases of the skin and subcutaneous tissue.  Diseases of the musculoskeletal system and connective tissue.  Congenital anomalies.  Certain conditions originating in the perinatal period.  Symptoms, signs, and ill-defined conditions.  Injury and poisoning. | Sex. | Patients of the Oklahoma State University Center for Health Systems Innovation (CHSI).  Country: USA | Data from the Oklahoma State University Center for  Health Systems Innovation (CHSI). The data warehouse contains an EMR on the visits of 58 million unique patients across 662 US hospitals (2000–2016). |
| Examining multimorbidity differences across racial groups: a network analysis of electronic medical records [122] | Network nodes | Infectious and parasitic diseases (1). Neoplasms (2). Endocrine. Nutritional and immunity disorders (3). Blood and blood-forming organs disorders (4). Nervous system disorders (6). Sense organ disorders (7). Pregnancy related diseases (12). Skin diseases (13). Musculoskeletal system disorders (14). Congenital anomalies (15). Perinatal period disorders (16) and injury/ poisoning (18) | Race/ethnicity. | Data from the Center for Health Systems Innovation at Oklahoma State University, which Conducts research on HIPAA compliant patient data provided by Cerner Corporation, a major Electronic Medical Record (EMR) provider.  Country: USA | This study recorded 14.1 million Whites, 3.46 million African Americans, 592,725 Hispanics, 400,521 Asians, 157,880 Native Americans, 25,414 Pacifc Islanders and 29,654 Biracial patients during the period 2000-2015. |
| Factors Associated With Multimorbidity Patterns in Older Adults in England: Findings From the English Longitudinal Study of Aging (ELSA) [52] | Latent class analysis | Cardiorespiratory/arthritis/cataracts class.  Metabolic class. | Sex, age, education, net financial wealth and lifestyle risk factor (smoking, drinking, physical activity level). | A representative cohort of men and women aged 50 or older living in England.  Country: UK | Data from the English Longitudinal Study of Aging (ELSA). This study analyzed data at Wave 2 (2004/2005). The sample at Wave 2 consisted of 9,171 participants |
| General practitioners records are epidemiological predictors of comorbidities: An analytical cross-sectional 10-year retrospective study [123] | Network nodes | Related models | Gender and age. | Patients living in the Salerno area (Italy).  Country; Italy | This cross-sectional study concerns a secondary analysis of the General Practitioners prescriptions of a population of 14,958 patients living in the Salerno area (Italy). |
| Global Multimorbidity Patterns: A Cross-Sectional, Population-Based, Multi-Country Study [105] | Exploratory factor analysis | Cardio-respiratory.  Metabolic.  Mental-articular.  Respiratory.  Other 1 (angina, cataract, diabetes, edentulism, hypertension and stroke).  Other 2(cataract, diabetes and stroke) | Country. | Older population (older than 50 years) non- institutionalized from Collaborative Research on Ageing in Europe project (Finland, Poland, and Spain) and the World Health Organization’s Study on Global Ageing and Adult Health (China, Ghana, India, Mexico, Russia, and South Africa).  Countries: Finland, Poland, Spain, hina, Ghana, India, Mexico, Russia, and South Africa. | Data from the Collaborative Research on Ageing in Europe and Study on Global AGEing and Adult Health studies. Nationally representative samples. These cross-sectional studies obtained data from 41,909 non- institutionalized older adults. |
| Identifying co-occurrence and clustering of chronic diseases using latent class analysis: cross-sectional findings from SAGE South Africa Wave 2 [53] | Latent class analysis | Concordant MM (high probabilities of having hypertension and diabetes).  Discordant MM (angina, asthma, chronic lung disease, arthritis and depression)). | Age, sex,BMI, years educated, alcohol, tobacco, add salt at table, self-reported vigorous intensity activity, residence, household wealth tertile and sleep quality. | South Africa. China, Ghana, India, Mexico and the Russian Federation. At least 45 years of age.  Country: South Africa. China, Ghana, India, Mexico and the Russian Federation. | This study was a cross-sectional study with 1967 individuals. |
| Identifying longitudinal clusters of multimorbidity in an urban setting: A population-based cross-sectional study [85] | Hierarchical cluster analysis (HCA) | Cluster A: Mental health+.  Cluster B: Cardiovascular+.  Cluster C: Pain+.  Cluster D: Liver+.  Cluster E: Dependence+.  Cluster F:Patients that do not belong to any cluster. | Gender, age, ethnicity, IMD quintile, alcohol, smoke and substance use. | Inner-city borough in south London, patients aged 18 years and over.  Country: UK | This is a population-based retrospective cross-sectional study using electronic health records of all adults aged 18 years and over, registered between April 2005 to May 2020 in general practices in one inner London borough. There were 826,936 patients registered. |
| Identifying multimorbidity patterns of non-communicable diseases in paediatric inpatients: a cross-sectional study in Shanghai, China [130] | Expert knowledge | patterns of multimorbidity among the different sex and age groups. | Age and sex. | Population aged 17 years and younger in Pudong New Area Shanghai, China.  Country: China | Cross-sectional study with a total of 193 432 paediatric inpatients. |
| Identifying Patterns of Multimorbidity in Older Americans: Application of Latent Class Analysis [54] | Latent class analysis | Minimal disease.  Nonvascular.  Vascular.  Cardio-stroke-cancer. Neurological disease.  Very sick. | Age, sex, ethnic, education level and marital status. | Medicare patients on the basis of age ≥65.  Country: USA | This study was a retrospective cohort study with 14052 participants in the Medicare Beneficiary Survey who had data available for at least 1 year after index interview. |
| Inequalities in multimorbidity among elderly: a population-based study in a city in Southern Brazil [106] | Factor analysis | Respiratory factor.  Cardio-metabolic factor.  Musculoskeletal/Mental/Functional disorders factor. | Economic level and educational level. | ≥ 60-year-old individuals from the urban area of Pelotas (South of Brazil).  Country: Brazil | This was a cross-sectional with a total of 1,844 elderly people identified, and 1,451 people interviewed. |
| Latent class analysis of multimorbidity patterns and associated outcomes in Spanish older adults: a prospective cohort study [55] | Latent Class Analysis | “Healthy” class. “Cardiorespiratory/mental/arthritis” class.  “Metabolic/stroke” class. | Age, sex, years schooling, income, marital status, number of medical visits and hospital admission. | Non-institutionalized people aged 50 years old and over living in Spain.  Country: Spain | The present study used data from “Edad con Salud”, a nationally representative survey of adult, non- institutionalized people in Spain. A total of 4753 persons participated in face-to-face structured interviews conducted at their homes between 2011 and 2012. |
| Learning multimorbidity patterns from electronic health records using Non-negative Matrix Factorisation [128] | Non-negative Matrix factorisation | Non-negative Matrix Factorisation (NMF) | Sex. | Patients with at least 5 years of follow-up  Country: UK | Patients with at least 5 years of follow-up; this resulted in a total number of 2,204,178 patients |
| Lifestyle and Socioeconomic Determinants of Multimorbidity Patterns among Mid-Aged Women: A Longitudinal Study [107] | Factor analysis | Psychosomatic.  Musculoskeletal.  Cardiometabolic.  Cancer.  Respiratory. | BMI, Physical activity, smoking, alcohol intake, education, occupation and ability to manage on income. | Self-reported women aged 45-50.  Country: Australia | Participants were from the Australian Longitudinal Study on Women’s Health. We included 4896 women |
| Multilevel Analysis of the Patterns of Physical-Mental Multimorbidity in General Population of São Paulo Metropolitan Area, Brazil [100] | Principal component analysis | **Women**  Irritable mood and headache.  Chronic disease and chronic pain.  Substance use disorders.  **Men**  Chronic pain and respiratory disease.  Psychiatric disorders.  Chronic diseases. | Sex, age, marital status, education, family income, mental health-care, Gini coefficient, area violence, area-level education and area level income were collected. | Individuals aged 18 years or older living in the São Paulo metropolitan area were selected through stratified, multistage area probability sampling. This area comprises the city of São Paulo and its 38 surrounding municipalities.  Country: Brazil | Cross-sectional data were drawn from the São Paulo Megacity Mental Health Survey. At the time of data collection, from May 2005 to May 2007. Sample of 4,615 subjects. |
| Multimorbidity among two million adults in China [86] | Hierarchical cluster analysis (HCA) | Cluster analysis (middle-aged male).  Cluster analysis (older male).  Cluster analysis (middle-aged female).  Cluster analysis (middle-aged female).  Cluster analysis (middle-aged female). | Sex and age. | Chinese people aged ≥45 years.  Country: China | This study used data between January 1st 2011 and December 31st 2015 from the Beijing Medical Claim Data for Employees. 2,097,150 participants |
| Multimorbidity Analysis of 13 Systemic Diseases in Northeast China [124] | Network nodes | Related models | Sex and age. | People who were hospitalized in 20 general hospitals in Jilin Province.  Country: China | A large-scale cross-sectional survey was implemented among people who were hospitalized in 20 general hospitals in Jilin Province in 2017. A total sample size of 1,300,683 inpatients |
| Multimorbidity and functional status in older people: a cluster analysis [87] | K-means clustering | Cluster 1, 2, 3 and 4. | Age, sex, educational attainment and BMI. | People aged 70 years or older, dependent and resident in Gipuzkoa (Spain).  Country: Spain | This was a cross sectional study with a total of 813 individuals. |
| Multimorbidity and health-related quality of life (HRQoL) in a nationally representative population sample: implications of count versus cluster method for defining multimorbidity on HRQoL [88] | Hierarchical clustering analysis (HCA) | Cluster 1: Heart or circulatory condition and arthritis.  Cluster 2: Anxiety disorder, major depression disorders. | Sex, age, BMI, labour force status, level of exercise, marital status, smoking status and index of socio-economic disadvantage-area deciles. | Respondents of the National Survey of Mental Health and Wellbeing conducted by the Australian Bureau of Statistics aged 16 to 85 years of age and living in private dwellings.  Country: Australia | A cross-sectional analysis of a nationally representative dataset, the 2007 National Survey of Mental Health and Wellbeing with  8841 patients |
| Multimorbidity and Hospital Admissions in High-Need, High-Cost Elderly Patients [56] | Latent class analysis | Class 1: Metabolic-ischemic heart disease.  Class 2: Neurological.  Class 3: Heart impairment.  Class 4: Cardio-respiratory.  Class 5: Cancer | Hospital admission. | Population in the province of Vicenza, in northeast Italy older than 65 years old.  Country: Italy | Data of a population on 190,000 residents served by the local health units. |
| Multimorbidity and Its Patterns according to Immigrant Origin. A Nationwide Register-Based Study in Norway [108] | Exploratory factor analysis technique | Mental health.  Respiratory/atopic.  Cardiovascular.  Cardio-endocrine.  Respiratory.  Mental-geriatric.  Muscular.  Malignant.  Endocrine.  Musculoskeletal.  Mental-psychosomatic.  Complex endocrine.  Mental-psychiatry.  Haematological.  Other. | Gender, age category and the three defined immigrant groups separately (Western countries (West Europe & North America), Eastern Europe,Other Non-Western (Asia, Africa & Latin America ). | Norwegian and Norway’s immigrant and older than 15 years old.  Country: Norway | This register-based study relies on merged data from the National Population Register and the Norwegian Health Economics Administration database. During the year 2008: 3,349,721 Norwegians and 389,807 immigrants were included in the study. |
| Multimorbidity gender patterns in hospitalized elderly patients [129] | Expert knowledge | Metabolic diseases (chronic  kidney disease, diabetes mellitus, hypertension, dyslipidemia, and obesity).  Cardiovascular  diseases (ischemic heart disease, heart failure, arrhythmias, cerebrovascular disease, and  peripheral vascular disease).  Respiratory diseases (chronic obstructive pulmonary disease,  asthma, pulmonary fibrosis, and sleep apnea).  Neurological-psychiatric diseases (neurological motor diseases, dementia, depression, and anxiety).  Osteoarticular diseases (osteoarthritis, osteoporosis, and osteoporotic fractures).  Cancer (active neoplasm).  Miscellanea (autoimmune disease, bowel disease, hepatopathy, anemia, and thromboembolic disease). | Sex. | Patients admitted to a hospital medical ward specialized in the care of multimorbidity patients in the University Hospital Mutua de Terrassa.  Country: Spain | This was a prospective cohort study valuating all patients admitted to a hospital medical ward specialized in the care of multimorbidity patients in the University Hospital Mutua de Terrassa from September 1, 2015, to December 31, 2016. 975 admissions of 885 patients were analyzed |
| Multimorbidity in the community-dwelling elderly in urban China [117] | Factor analysis | Pattern 1: Hearing disorder, cataract, joint disease, cancer.  Pattern 2: Liver disease, lung disease, gastrointestinal disease.  Pattern 3: Dyslipidemia, diabetes, hypertension, coronary heart disease, kidney disease. | Sex and age. | The subjects should be aged 60 years and older and have lived for a long time (2 years or more) in Nanjing, Jiangsu province.  Country: China | A cross-sectional study was conducted in the community governed by a Community Health Service Center in Nanjing, Jiangsu province. 2452 persons were enrolled. |
| Multimorbidity Patterns and 6-Year Risk of Institutionalization in Older Persons: The Role of Social Formal and Informal Care [89] | fuzzy c-means cluster | Pattern 1: Unspecific  Pattern 2: Musculoskeletal, respiratory and gastrointestinal.  Pattern 3: Sensory impairments and cancer.  Pattern 4: Metabolic and sleep disorders.  Pattern 5: Cardiovascular diseases, anemia and dementia.  Pattern 6: Psychiatric. | Age, sex, education and marital status. | Adults living in the Kungsholmen district of Stockholm, Sweden, who are age 60 years or older.  Country: Sweden | Data from the Swedish National study on Aging and Care in Kungsholmen were used to answer the research questions. 2571 community- dwelling participants with multimorbidity at baseline remained for the analyses. They were followed up for 6 years |
| Multimorbidity Patterns and Memory Trajectories in Older Adults: Evidence From the English Longitudinal Study of Aging [57] | Latent class analysis | Class 1: Heart Disease/Stroke.  Class 2: Asthma/Lung Disease.  Class 3: Arthritis/Hypertension.  Class 4: Depression/Arthritis.  Class 5: Hypertension/Cataracts/Diabetes.  Class 6: Psychiatric Problems/Depression.  Class 7: Cancer.  Class 8: Arthritis/Cataracts. | Age, gender, smoke, physical activity, marital status, wealth, education and BMI. | Older adults of UK.  Country: UK | The sample consisted of 11 449 respondents from the English Longitudinal Study of Aging |
| Multimorbidity patterns and risk of frailty in older community-dwelling adults: a population-based cohort study [77] | Latent class analysis | Pattern 1: Unspecific.  Pattern 2: musculoskeletal, respiratory & gastrointestinal diseases.  Pattern 3: Respiratory & gastrointestinal diseases.  Pattern 4: Metabolic & sleep disorder.  Pattern 5: Sensory impairments & cancer cardiovascular diseases.  Pattern 6: Anemia & dementia.  Pattern 7: Psychiatric. | Age, sex, education, civil status, alcohol consumption and smoking status. | Older adults, non institutionalised, living in Kungsholmen a district of Stockholm, Sweden.  Country: Sweden | Data from the Swedish National study on Aging and Care in Kungsholmen. The cross-sectional analyses included 2,534 participants |
| Multimorbidity patterns and their related characteristics in European older adults: A longitudinal perspective [58] | Latent class analysis | Severely impaired class.  Osteoarticular class.  Metabolic class.  Healthy class. | Sex, age, smoke status, education level, marital status, BMI, employment status, deprivation level and physical activity. | Older adults aged 50+ from Austria, Belgium, Czech Republic, Denmark, Estonia, France, Germany, Italy, Luxembourg, Slovenia, Spain, Sweden and Switzerland and Girona, a single region within Spain.  Country: Austria, Belgium, Czech Republic, Denmark, Estonia, France, Germany, Italy, Luxembourg, Slovenia, Spain, Sweden and Switzerland | A population-based longitudinal European study. 25,931 drawn from the Survey of Health, Ageing and Retirement in Europe |
| Multimorbidity patterns and their relationship to mortality in the US older adult population [59] | Latent class analysis | Class 1: Complex cardiometabolic.  Class 2: Cognitively impaired.  Class 3: Respiratory condition.  Class 4: Age-associated condition.  Class 5: Healthy. | Age, gender, ethnicity, education, marital status, income, health insurance, smoking, drinking and BMI. | US civilian non-institutionalized. Black, Hispanic, Asian and adults 50 and older.  Country: USA | This cross-sectional study with longitudinal mortality follow-up utilized data from NHIS 2002 to 2014. The study sample were 166,126 participants |
| Multimorbidity Patterns in Elderly Primary Health Care Patients in a South Mediterranean European Region: A Cluster Analysis [90] | Hierarchical cluster analysis (HCA) | **Women aged 65–79 years**  Cluster 1:  Hypertensive diseases  Metabolic disorders  Arthrosis  Obesity and other hyperalimentation  Diabetes mellitus.  Cluster 2:  Other dorsopathies  Disorders of bone density and structure  Other soft tissue disorders  Diseases of veins, lymphatic vessels and lymph nodes, not  elsewhere classified  Neurotic, stress-related and somatoform disorders  Other joint disorders.  Cluster 3:  Other forms of heart disease  Mood [affective] disorders  Disorders of thyroid gland  Other diseases of intestines  Disorders of lens  Diseases of oesophagus, stomach and duodenum  Chronic lower respiratory diseases  Noninflammatory disorders of female genital tract  Benign neoplasms  Hernia  Glaucoma.  Cluster 4:  Diseases of oral cavity, salivary glands and jaws  Other disorders of ear  Acute upper respiratory infections  Dermatitis and eczema  Behavioural syndromes associated with physiological  disturbances and physical factors  Episodic and paroxysmal disorders  Other diseases of urinary system  Other diseases of upper respiratory tract  Nerve, nerve root and plexus disorders  Mycoses  **Men aged 65–79 years**  Cluster 1:  Hypertensive diseases  Metabolic disorders  Diseases of male genital organs  Diabetes mellitus  Obesity and other hyperalimentation.  Cluster 2:  Other dorsopathies  Arthrosis  Other soft tissue disorders  Diseases of oral cavity, salivary glands and jaws  Other joint disorders  Other disorders of ear.  Cluster 3:  Chronic lower respiratory diseases  Mental and behavioural disorders due to psychoactive  substance use.  Cluster 4:  Other forms of heart disease  Ischaemic heart diseases.  **Women aged 80 years**  Cluster 1:  Hypertensive diseases  Metabolic disorders  Arthrosis  Other forms of heart disease.  Cluster 2:  Diseases of veins, lymphatic vessels and lymph nodes, not  elsewhere classified  Disorders of lens  Disorders of bone density and structure  Other dorsopathies  Other diseases of intestines  Neurotic, stress-related and somatoform disorders  Other soft tissue disorders  Other joint disorders.  Cluster 3:  Diabetes mellitus  Obesity and other hyperalimentation.  Cluster 4:  Obesity and other hyperalimentation  Diseases of oesophagus, stomach and duodenum  Hernia.  **Men aged 80 years**  Cluster 1:  Hypertensive diseases, diseases of male genital organs, metabolic disorders, other forms of heart disease, arthrosis, chronic lower respiratory diseases, diabetes mellitus.  Cluster 2:  Dosprders of lens, other dorsopathies of intestines, Hernia, diseases of esophagus, stomach and duodenum, Diseases of veins, lymphatic vessels, and lymph nodes, not elsewhere classified, other disorders of ear, other soft tissue disorders.  Cluster 3:  Ischaemic heart diseases, Renal failure, Diseases of arteries, arterioles and capillaries, Aplastic and other amaemias.  Cluster 4:  Obesity and other hyperalimentation, Glaucoma, Inflamatory polyarthropaties, Diseases of external ear. | Age and sex. | Catalonia, Spain. Older than 64 years.  Country: Spain | A cross-sectional study with 322,328 patients with multimorbidity. Data from 251 primary care centers in Catalonia, Spain. |
| Multimorbidity patterns in low-middle and high income regions: a multiregion latent class analysis using ATHLOS harmonised cohorts [60] | Latent class analysis | Cardio-metabolic. Respiratory-mental-articular. Healthy. | Age, lonelinesss, smoke and physical activity. | Self-reported demographic variables. 50 years old or older. From Africa, China, India, Russia, England, Northern Europe, Western Europe.  Country: Ghana, South Africa, UK, Denmark, Sweden, Greece, Italy, Spain, Austria, Belgium, France, Germany, Israel, Netherlands, Switzerland. | The total sample was 72 140 people aged 50+ years from three population-based studies (English Longitudinal Study of Ageing, Survey of Health, Ageing and Retirement in Europe Study and Study on Global Ageing and Adult Health) included in the Ageing Trajectories of Health: Longitudinal Opportunities and Synergies. |
| Multimorbidity patterns in old adults and their associated multi-layered factors: a cross-sectional study [61] | Latent class analysis | Pattern 1: degenerative/digestive diseases.  Pattern 2: cardiovascular diseases.  Pattern 3: metabolic disease. | Age, sleep quality, physical exercise, balanced diet, friendship network, social support, education, per capita monthly family income and types of basic medical insurance. | Old adults aged 60+ without cognitive impairment living in the selected residential building/villagers’ groups in Shanxi Province.  Country: China | Data from a cross-sectional questionnaire based study, that surveyed a total of 7480 participants |
| Multimorbidity patterns in primary care: interactions among chronic diseases using factor analysis [101] | Factor analysis | Cardio-metabolic.  Psychiatric-substance abuse. Mechanical-obesity-thyroidal. Psychogeriatric and depressive. | Sex and age. | Individuals belonging to 19 urban health centres (7 in Aragón and 12 in Catalonia) over the age of 14 whowere seen at least once during 2008 by their family doctor were included in the study.  Country: Spain | This observational, retrospective, multicentre study utilised information from the electronic medical records of 19 primary care centres from 2008. The study sample was 275,682 individuals |
| Multimorbidity patterns in the elderly: a prospective cohort study with cluster analysis [91] | K-means clustering | Nonspecific.  Musculoskeletal.  Endocrine-metabolic.  Digestive/digestive- respiratory.  Neuropsychiatric. Cardiovascular diseases | Sex and age. | 65–94 years of age in Barcelona, Catalonia (Spain).  Country: Spain | A cohort study with yearly cross-sectional analysis of electronic health records. Records from 190,108 patients with multimorbidity |
| Multimorbidity Patterns in the General Population: Results from the EpiChron Cohort Study [109] | Factor Analysis | **Women**  0-14 years:  Growth-development. Allergic.  15-29 years:  Allergic-growth-development. Endocrine-metabolic. Mental health.  30-44 years:  Endocrine. Metabolic. Neuromuscular-depressive  45-59 years:  Peripheral vascular. Metabolic. Neuromuscular-depressive.  45-59 years:  Peripheral vascular. Metabolic. Neuromuscular-depressive.  60-74 years:  Peripheral vascular. Metabolic. Neuromuscular-depressive. Cardiorespiratory.  75-89 years:  Neurodegenerative-vascular. Metabolic. Neuromuscular-degenerative. Cardiorespiratory.  ≥90 years:  Neuromuscular-degenerative. Cardiorespiratory.  **Men**  0-14 years:  Growth-development. Allergic.  15-29 years:  Allergic-growth-development. Metabolic. Mental health.  30-44 years:  Allergic. Metabolic. Metabolic. Mental health. Sensory processing.  45-59 years:  Cardiovascular. Metabolic. Mental health. Neuromuscular. Neuromuscular-depressive.  60-74 years:  Cardiorespiratory. Metabolic. Peripheral vascular and neuromuscular-depressive.  75-89 years:  Cardiorespiratory. Metabolic. Neurodegenerative-vascular. Neuromuscular-degenerative.  ≥90 years:  Cardiorespiratory. Neuromuscular-degenerative. Neurodegenerative-vascular. | Sex and age. | All public health system users of the Spanish region of Aragon.  Country: Spain | A retrospective, observational study based on data from the EpiChron Cohort Study realised between 1 January 2011 and 31 December 2011. The total sample was 1,253,292. |
| Multimorbidity patterns of chronic conditions and geriatric syndromes in older patients from the MoPIM multicentre cohort study [92] | Fuzzy C- means cluster analysis | Osteo-articular.  Psychogeriatric. M  Minor chronic disease.  Cardiorespiratory | Age, sex and household (alone, with relatives or other people, in a nursing  Home) | Older patients (≥65 years old) hospitalised at the internal medicine or geriatric services at five general teaching hospitals in three different regions of Spain.  Country: Spain | Multicentre, prospective cohort study. Sample of 740 patients a hospitalized of their chronic conditions between September 2016 and December 2018. |
| Multimorbidity patterns with K-means nonhierarchical cluster analysis [93] | K-means clustering | 6 cluster of each sex | Sex and age. | People from Catalonia (Spain).  Country: Spain | Cross-sectional study using electronic health records from 523,656 patients. Data were provided by the Information System for the Development of Research in Primary Care |
| Multimorbidity patterns, all-cause mortality and healthy aging in older English adults: Results from the English Longitudinal Study of Aging [62] | Latent class analysis | Cardiorespiratory/arthritis/cataracts.  Metabolic.  Relatively healthy. | Sex, age and education. | Men and women aged ≥50 years living in England.  Country: UK | Data from 9171 individuals of the English Longitudinal Study of Aging. The study commenced in 2002, and has been followed up every 2 years. |
| Multimorbidity Patterns, Frailty, and Survival in Community-Dwelling Older Adults [63] | Latent class analysis | Minimal disease.  Cardiovascular disease. Osteoarticular.  Neuropsychiatric.  High multisystem morbidity. | Age, sex and race. | Medicare beneficiaries aged 65 years and older.  Country: USA | 7,197 community-dwelling adults. Data are from the first five yearly rounds of NHATS, starting in 2011. |
| Multiple Chronic Conditions and Hospitalizations Among Recipients of Long-Term Services and Supports [64] | Latent Class Analysis | Cardiopulmonary.  Cerebrovascular.  All other. | Age, gender, race, ethnicity, marital status, education and income. | Ages 60 and older, enrolled in long term-services and supports within the preceding 60 days.  Country: USA | A secondary data analysis was conducted using an extant data set from a National Institute on Aging-funded longitudinal observational cohort study entitled, Health Related Quality of Life: Elders in Long Term Care. The parent study enrolled 470 participants. |
| Multiple chronic conditions: Implications for cognition - Findings from the Wisconsin Registry for Alzheimer's Prevention (WRAP) [65] | Latent Class Analysis | Depression.  Sleep.  Cardiovascular.  Healthy. | Gender, age, race, marital status, education, education in years, employment status and BMI | Subjects were English-speaking and 36 to 68 years of age and cognitively intact at the time of enrollment. Rolling enrollment for WRAP began in November 2001 and continues. WRAP participants are predominantly adult biological children of persons with Alzheimer’s Disease (AD), either confirmed by autopsy or deemed probable as defined by the National Institute of Neurological and Communicative Disorders and Stroke- Alzheimer’s Disease and Related Disorders Association research criteria, WRAP also enrolls healthy controls with no parental history of AD or dementia.  Country: USA | Analysis of existing longitudinal data derived from the Wisconsin Registry for Alzheimer’s Prevention with 1285 participants. Data are collected at the baseline, at approximately 4 years’ postbaseline, and then every 2 years thereafter. |
| Patrones de multimorbilidad en adultos jóvenes en Cataluna: un análisis de clústeres [94] | Hierarchical cluster analysis (HCA) | **Mujeres**  **19-24**  Cluster 1:Diseases of the oral cavity and salivary glands, acute upper respiratory tract infections, non-inflammatory disorders of the female genital tract, mental and behavioral disorders due to the consumption of psychoactive substances, other dorsopathies, neurotic disorders, stress-related disorders and somatoform disorders, disorders of the phaneres, dermatitis and eczema.  Cluster 2: Other joint disorders, Episodic and paroxysmal disorders, Benign tumors, Disorders of ocular muscles, binocular movement, accommodation and refraction, Deforming dorsopathies, Viral infections characterized by skin and mucous membrane lesions, Other soft tissue disorders, Deficiency anemias, Urticaria and erythema, Breast disorders.  Cluster 3: Intestinal infectious diseases, Other urinary tract diseases, Conjunctival disorders, Other viral diseases, Influenza and pneumonia, Other acute lower respiratory tract infections, Disorders of the eyelid, lacrimal apparatus and orbit.  Cluster 4: Poisoning by, adverse effects and underdosage of drugs, medicines and substances, Mycoses, Inflammatory diseases of the female pelvic organs, Infections with predominantly sexual mode of transmission.  **25-44**  Cluster 1:Other dorsopathies, Neurotic disorders, stress-related disorders and somatoform disorders, Mental and behavioral disorders due to the use of psychoactive substances, Non-inflammatory disorders of the female genital organs, Acute infections of the upper respiratory tract, Diseases of the oral cavity, salivary glands and jaws.  Cluster 2: Benign tumors, Other soft tissue disorders, Episodic and paroxysmal disorders, Other joint disorders, Diseases of veins and vessels and lymph nodes, not elsewhere classified, Nutritional anemias, Diseases of the esophagus, stomach and duodenum, Disorders of the thyroid gland, Disorders of the breast, Other diseases of the intestines.  Cluster 3:Dermatitis and eczema, Fan disorders, Mycosis, Poisoning by drugs, medicines and biological substances, Viral infections characterized by lesions of the skin and mucous membranes, Urticaria and erythema.  Cluster 4:Obesity and other types of hyperalimentation, Metabolic disorders, Hypertensive diseases, Diabetes mellitus.  **Hombres**  **19-24**  Cluster 1:Diseases of the oral cavity and salivary glands, Mental and behavioral disorders due to psychoactive substance use, Acute upper respiratory tract infections, Disorders of the pharynx, Other dorsopathies, Neurotic disorders, stress-related disorders and somatoform disorders.  Cluster 2:Other joint disorders, Dermatitis and eczema, Other soft tissue disorders, Viral infections characterized by lesions of the skin and mucous membranes, Mycoses, Urticaria and erythema.  Cluster 3:Benign tumors, Disorders of ocular muscles, binocular movement, accommodation and refraction, Diseases of the male genital organs, Deforming dorsopathies, Behavioral and emotional disorders whose onset usually occurs in childhood and adolescence, Visual disturbances and blindness.  Cluster 4: Trauma of unspecified part of the trunk, limb or region of the body, Trauma of the wrist and hand, Trauma of the ankle and foot, Trauma of the knee and leg.  **25-44**  Cluster 1: Mental and behavioral disorders due to the use of psychoactive substances, Other dorsopathies, Diseases of the oral cavity, salivary glands and jaws, Acute upper respiratory tract infections, Neurotic disorders, stress-related disorders and somatoform disorders, Other joint disorders, Other soft tissue disorders.  Cluster 2: Metabolic disorders, Obesity and other types of hyperalimentation, Hypertensive diseases.  Cluster 3: Other diseases of the upper respiratory tract, Chronic diseases of the lower respiratory tract.  Cluster 4: Benign tumors, Disorders of the pharynx, Viral infections characterized by lesions of the skin and mucous membranes. | Sex and age. | People with multimorbidity, aged 19 to 44 years.  Country: Spain | A descriptive transversal study with 530.798 patients |
| Patterns and Consequences of Multimorbidity in the General Population: There is No Chronic Disease Management Without Rheumatic Disease Management [66] | Latent class analysis | Pattern 1: low disease probability.  Pattern 2: cardio metabolic conditions.  Pattern 3: respiratory conditions.  Pattern 4: RMDs and depression. | Sex, age, BMI and smoking. | Portuguese National Health Survey. Participants aged 18 years or older.  Country: Portugal | Cross-sectional data from the Portuguese Fourth National Health Survey were analyzed (n = 23,754) |
| Patterns of Chronic Conditions and Their Associations With Behaviors and Quality of Life, 2010 [67] | Latent class analysis | Class 1: Healthy.  Class 2: Physical Health Conditions.  Class 3: Mental Health Conditions.  Class 4: Physical and Mental Health Conditions. | Tobacco use, BMI, leisure-time aerobic activity, strength training and aerobic activity strength training. | US population aged 18 years or older.  Country: USA | Data obtained from the summer wave of Porter Novelli’s 2010 HealthStyles database. 4,184 adults answered the questions |
| Patterns of chronic physical multimorbidity in psychiatric and general population [68] | Latent class analysis | Relatively healthy.  Musculoskeletal.  Hypertension and obesity.  Complex multimorbidity. | Age and gender. | “Somatic Comorbidities in psychiatric patients” at the Psychiatric Hospital Sveti Ivan, Zagreb, Croatia and European health interview survey. The inclusion criterion for both populations was being ≥18 years of age.  Country: Croatia | Cohort study. A sample of 1060 psychiatric patients and 837 participants from the general population. This analysis compares the results from two cross-sectional studies (Somatic Comorbidities in psychiatric patients” and European health survey) |
| Patterns of multimorbidity and demographic profile of latent classes in a Danish population-A register-based study [69] | Latent class analysis | **+65 years**  No or few diseases’.  ‘Diabetes, cholesterol’.  ‘Heart diseases’.  ‘Back disease, asthma allergy’.  ‘Many diseases’.  ‘COPD, cancer,  liver disease’.  ‘Mental illness,epilepsy’.  **45-64 years**  No or few diseases’.  ‘Diabetes, cholesterol’.  ‘Bone-, joint diseases’.  ‘Mental illness, epilepsy’.  ‘Heart diseases’.  ‘Many diseases’.  ‘Asthma, allergy’.  **-45 years**  ‘No or few  diseases’.  ‘Bone-, joint diseases’.  ‘Mental illness, epilepsy’.  ‘Asthma, allergy’.  ‘Diabetes, heart diseases’. | Age, sex, country of origin, marital status, employment and education. | All persons aged 16 years or older with permanent residence in Denmark.  Country: Denmark | Sample of 470,794 Danish population. This was drawn from register the Danish Civil Registration System. Only diseases that led to hospital contact within the previous 5 or 10 years according to the disease were included |
| Patterns of multi-morbidity and prediction of hospitalisation and all-cause mortality in advanced age [95] | Hierarchical cluster analysis (HCA) | **Maori patterns**  ‘Well’ (lower prevalence of  conditions).  Congestive heart failure and  atrial fibrillation.  Arthritis.  Cardiovascular disease, respiratory and mental health.  Diabetes.  Complex multimorbidity  (high prevalence of most conditions).    **Non maori patterns**  ‘Well’ (lower prevalence of conditions).  Congestive heart failure and  atrial fibrillation.  Depression and arthritis.  Cancer.  Respiratory and diabetes.  Stroke. | Age, sex and Deprivation Index. | A population based sample of Māori aged 80–90 years and non-Māori aged 85 years from a regionally defined area in the North Island.  Country: New Zealand | A sample of 421 Māori and 516 non-Māori. Data from Te Puāwaitanga O Nga Tapuwae Kia ora Tonu: Life and Living in Advanced Age; a Cohort Study in NZ (LiLACS NZ) is a cohort study of Māori (indigenous people in New Zealand) and non-Māori octogenarians |
| Patterns of multimorbidity in 4588 older adults: Implications for a nongeriatrician specialist [110] | Factor analysis | **55-59 years**  Pattern 1: Arrythmia, congestive heart failure and respiratory disease.  Pattern 2: Hypertension, eye disorder, impaired cognition, chronic kidney disease.  Pattern 3: impaired cognition, parkinson disease or epilepsy  Pattern 4: Psychiatric disorder, thyroid disease, osteoporosis.  **65-79 years**  Pattern 1: Arrythmia, ischemic heart disease and congestive heart failure.  Pattern 2: Psychiatric disorder, impaired cognition, Parkinson disease or epilepsy, stroke.  Pattern 3: Thyroid, osteoporosis, eye disorder  Pattern 4: Hypertension, metabolic disorders, obesity.  Pattern 5: Anemia, chronic kidney disease.  **≥80 years**  Pattern 1: arrhythmia, respiratory, ischemic heart disease, congestive heart failure.  Pattern 2: metabolic disorders, obesity.  Pattern 3: Anemia, cancer, chronic disease.  Pattern 4: osteoporosis, eye disorder  Pattern 5: impaired cognition | Age. | Patients aged 55 to 59 years and 65 years or older in Poland.  Country: Poland | Data from the PolSenior was a nationwide, multicenter, cross-sectional survey of the aged population in Poland. 4588 data participants were included |
| Patterns of Multimorbidity in a Population-Based Cohort of Older People: Sociodemographic, Lifestyle, Clinical, and Functional Differences [96] | Fuzzy c-means  cluster analysis algorithm | PSY-RESP: psychiatric and respiratory diseases.  HEART: heart diseases.  EYE-CANCER: eye diseases and cancer.  CNS-IMP: cognitive and sensory  Impairments.  RESP-MSK: respiratory and musculoskeletal diseases.  UNSPECIFIC.  ALL. | Sex, age, civital status, education, occupation, social network, smoking, alcohol consumption and physical activity. | Community-dwelling and institutionalized older adults aged 60 years and older. Of people born between 1898 and 1943, living in the Kungsholmen district of Stockholm (Sweden).  Country: Sweden | Data from the Swedish National Study on Aging and Care in Kungsholmen on 2,931 participants. Those who accepted were evaluated between 2001 and 2004 for the first time and subsequently followed up every 6 years (those aged <78 years) or every 3 years (those aged ≥78 years) |
| Patterns of Multimorbidity in Adults: An Association Rules Analysis Using the Korea Health Panel [125] | Network nodes | Network of frequent disease based on centrality by gender and age.  (a) men (HTN, PH, and dyslipidemias)  (b) women (HTN, osteoporosis without pathological fracture, dyslipidemias, gastritis, polyarthrosis, and DM)  (c) men aged under 65 (HTN, dyslipidemias, DM, gastritis, and allergic diseases)  (d) women aged under 65 (HTN, dyslipidemias, gastritis, polyarthritis, and other intervertebral disc disorders)  (e) men aged 65 or older(HTN, PH, DM, dyslipidemia, and cataract)  (f) women aged 65 or older (HTN, polyarthritis, osteoporosis, dyslipidemias, and cataract) | Sex and age. | 18 and older adults from the Korean Health Panel Survey.  Country: Korea | Data from the Korea Institute for Health and Social Affairs and the National Health Insurance Service. This was a cross-sectional study with 11,232 adults |
| Patterns of multimorbidity in the aged population. Results from the KORA-Age study [111] | Exploratory factor analysis | Cardiovascular/metabolic disorders.  Liver/lung/joint/eye disorders. Mental/neurologic disorders.  Gastrointestinal disorders and cancer. | Sex and age. | Individuals aged 65–94 who have participated in at least one of the four cross-sectional MONICA/ KORA surveys. These surveys have been conducted between 1984 and 2001 and included a random sample of the population of the city of Augsburg and its two surrounding counties in Southern Germany.  Country: Germany | Data from the present study derived from the “KORA-Age” study which is a follow-up. In total, 17,607 persons participated |
| Patterns of patients with multiple chronic conditions in primary care: A cross-sectional study [70] | Latent class analysis | Class 1: cardiometabolic diseases, IHD, kidney disease and anemia.  Class 2: cardiometabolic diseases and IHD.  Class 3: cardiometabolic diseases and obesity.  Class 4: cardiometabolic diseases and arthritis.  Class 5: cardiometabolic diseases and arthritis.  Class 6: cardiometabolic diseases, IHD and kidney disease.  Class 7: cardiometabolic diseases, obesity and arthritis.  Class 8: cardiometabolic diseases, obesity and arthritis. | Sex, age and country. | Aged 0-99 years who visited the National Healthcare Group Polyclinics.  Country: Singapore | A cross-sectional study of electronic medical records was conducted on 437,849 individuals. |
| Physical multimorbidity, depressive symptoms, and social participation in adults over 50 years of age: findings from the English Longitudinal Study of Ageing [71] | Latent class analysis | Relatively healthy.  Hypertension/diabetes. Respiratory.  Complex/multisystem. | Age, sex, net wealth, educational attainment, employment status, relationship status and smoking status. | English adults aged ≥50 years.  Country: UK | A Longitudinal Study with 11,391 participants. Data came from the English Longitudinal Study of Ageing (ELSA) |
| Prevalence and Patterns of Multimorbidity in a Nationally Representative Sample of Older Chinese: Results From the China Health and Retirement Longitudinal Study [97] | Hierarchical cluster analysis (HCA) | Cluster rural women.  Cluster urban women.  Cluster rural men.  Cluster urban men. | Gender and residencial regions. | Used a multi-stage sampling strategy covering 28 provinces, 150 counties or dis-tricts, and 450 villages or urban communities across the country.  Country: China | Results From the China Health and Retirement Longitudinal Study (2011-2015) and included 19,841 participants. |
| Prevalence and patterns of multimorbidity in Australian baby boomers: the Busselton healthy ageing study [72] | Latent class analysis | Relatively healthy.  Predominant respiratory & atopy.  Multi-morbid non cardio-metabolic.  Multi-morbid cardio-metabolic and other. | Age, sex, waist circumference, tobacco smoking, moderate/vigorous physical activity and alcohol consumption. | Adults born 1946–1964 residing within the City of Busselton’s local government electoral boundary in Western Australia.  Country: Australia | A cross-sectional phase of the Busselton Healthy Ageing Study (BHAS) between 2010 and 2015 with 5029 participants |
| Prevalence and Patterns of Multi-Morbidity in Serbian Adults: A Cross-Sectional Study [112] | Exploratory factor analysis | **20-44**  Men:  Factor 1: Non-communicable. Factor 2:Cardio-metabolic.  Women:  Factor 1: Non-communicable. Factor 2:Cardio-metabolic.  Factor 3: Respiratory.  **45-64**  Men:  Factor 1: Respiratory.  Factor 2: Cardio-metabolic.  Factor 3: Aggrerate.  Factor 4: Cardiovascular.  Women:  Factor 1: Cardio-metabolic.  Factor 2: Respiratory.  Factor 3: Cardiovascular.  **65 years or older**  Men:  Factor 1: Respiratory.  Factor 2: Cardio-metabolic.  Factor 3: Cardiovascular.  Factor 4: Mechanical/mental/metabolic  Women  Factor 1: Respiratory.  Factor 2: Cardio-metabolic.  Factor 3: Cardiovascular.  Factor 4: Mechanical/mental/metabolic | Sex and age. | National Health Survey (NHS 2013) of the Serbian population aged 20 or older.  Country: Serbia | A Cross-Sectional Study data from the 2013 National Health Survey, which included 13,103 individuals |
| Prevalence of multimorbidity in general practice: a cross-sectional study within the Swiss Sentinel Surveillance System (Sentinella) [136] | Expert knowledge | Cardiovascular. Neurological. Respiratory. Psychological. Hemato-immunological. Digestive. General. Metabolic. Ophthalmological. Ear. Dermatological. Urological. Female genital and male genital. | Age. | Patients of all ages in Switzerland.  Country: Switzerland | A cross-sectional study within the Swiss Sentinel Surveillance  System (Sentinella) in 2015. Data from 2904 patients |
| Similar multimorbidity patterns in primary care patients from two European regions: results of a factor analysis [118] | Exploratory factor analysis | **Spain**  Cardiometabolic.  Mechanical. Psychiatric-substance abuse. Depressive.  Psychogeriatric.  **Netherland**  Factor 1 (cardiovascular conditions,diabetes and obesity).  Factor 2 (psychosocial and neurologic diseases, and gastro-oesophageal reflux).  Factor 3 (musculoskeletal conditions, gastro-oesophageal reflux, hyperten-sion and depression.). | Sex and age. | Data for the Spanish population were obtained from electronic medical records of people over 14 years of age who consulted their general practitioner. Data for the Dutch population derived from the Registration Network Family Practices of The Netherlands.  Country: Netherlands | This observational, retrospective, multicentre study analysed information from primary care electronic medical records. The sample consists of 79,291 Dutch and 275,682 Spaniards. |
| Social Determinants and Health Behaviours among Older Adults Experiencing Multimorbidity Using the Canadian Longitudinal Study on Aging [132] | Expert knowledge | Cardiovascular/metabolic. Musculoskeletal.  Mental health | Age, sex, education level, household income, marital status, inmigration status, number of friends, relatives, housing problems, urban/rural, BMI, inactivity, sleep and appetite. | Canadian participants who were 65 years of age and older.  Country: Canada | A Longitudinal Study (2012-2015) Using baseline data from the Canadian Longitudinal Study on Aging (CLSA), 12272 Canadians |
| Social determinants of multimorbidity in Jamaica: application of latent class analysis in a cross-sectional study [73] | Latent class analysis | Relatively healthy class. Metabolic class.  Vascular-inflammatory class  Respiratory class. | Age and sex, socioeconomic position, housing to obtain fruit and vegetable, recreational areas in walking distance, greater perceived safety, neighbourhood infrastructure score, currently use alcohol, past or present smoker, low levels of physical activity, excessive fast-food consumption, consumes sugar-sweetened beverage at least consumption, consumes SSB at least once daily and health system. | A sample of 2848 Jamaicans, between 15 and 74 years.  Country: Jamaica | A cross-sectional study with a sample of 2551 respondents from the nationally representative Jamaica Health and Lifestyle Survey 2007/2008. |
| Soft clustering using real-world data for the identification of multimorbidity patterns in an elderly population: cross-sectional study in a Mediterranean population [98] | Fuzzy c-means algorithm | Pattern 1:Nervous and digestive.  Pattern 2: Respiratory, circulatory and nervous.  Pattern 3: Circulatory and digestive.  Pattern 4: Mental, nervous and digestive blood, female oldest-old dominant.  Pattern 5: Mental, nervous and digestive, female dominant.  Pattern 6: Nervous, musculoskeletal and circulatory female dominant.  Pattern 7: Genitourinary, mental and musculoskeletal, male dominant.  Pattern 8: Non- specified youngest-old dominant. | Sex, age, MEDEA index, number of drugs and number of visits. | Individuals aged 65–99 years of primary healthcare centres in Catalonia, Spain.  Country: Spain | A cross-sectional study (2012) with 916619 participants the Information System for Research in Primary Care (SIDIAP) database |
| Spreading of diseases through comorbidity networks across life and gender [126] | Network nodes | A and B: Certain infectious diseases.  C: Neoplasms.  D: Benign neoplasm.  E: Endocrine, nutritional and metabolic disease.  F: Mental and behavioural disorders.  G: Diseases of the nervous system.  H: Diseases of the eye and ear.  I: Diseases of the circulatory system .  J: Diseases of respiratory system.  K: Diseases of the digestive system.  L: Diseases of the skin and subcutaneous tissue.  M: Diseases of the musculoskeletal system.  N: Diseases of the genitourinary system. | Sex and age. | Persons receiving outpatient and inpatient care in Austria.  Country: Austria | Use a database of the Main Association of Austrian Social Security Institutions that contains pseudonymized claims data of all persons receiving outpatient and inpatient care in Austria between 1 January 2006 and 31 December 2007. The total sample of inpatients consists of 1,862,258 patients |
| Survival in relation to multimorbidity patterns in older adults in primary care in Barcelona, Spain (2010-2014): a longitudinal study based on electronic health records [99] | K-means clustering | Crude mortality rate.  Cardiovascular.  Digestive respiratory.  Endocrine metabolic.  Musculoskeletal.  Non-specific.  Standardized mortality rate. | Sex and age. | Adults aged 65–94 years in Barcelona, Spain.  Country: Spain | Prospective longitudinal observational study using electronic health records for 190108 people aged ≥65 years in Barcelona, Spain (2009–2014). |
| The burden of cardiovascular morbidity in a European Mediterranean population with multimorbidity: a cross-sectional study [134] | Expert knowledge | Cardiovascular morbidity group.  Non-cardiovascular morbidity group. | Age, sex, smoking, obesity and alcoholism. | Patients ≥19 years old assigned to 251 primary health care centres in Catalonia, Spain.  Country: Spain | A cross-sectional study in patients ≥19 years old assigned to 251 primary health care centres in Catalonia, Spain. A total of 1,749,710 individuals were included |
| The epidemiology of multimorbidity in primary care: a retrospective cohort study [131] | Expert knowledge | Patients with  Multimorbidity.  Patients with physical–mental comorbidity.  Patients with multimorbidity and physical–mental comorbidity | Sex, age, socioeconomic status and socioeconomic deprivation. | Adult patients (aged ≥18 years) who had been registered with a General Practicioners in England.  Country: UK | The study used a random sample of 403 985 adult patients (aged ≥18 years), who were registered with a general practice on 1 January 2012 and included in the Clinical Practice Research Datalink. |
| The influence of age, gender and socio-economic status on multimorbidity patterns in primary care. First results from the multicare cohort study [113] | Factor Analysis | Cardiovascular and metabolic disorders.  Anxiety, depression, somatoform disorders and pain. | Sex. | Multimorbid patients aged 65+ in 8 study centres distributed across Germany (Bonn, Düssedorf, Frankfurt/Main, Hamburg, Jena,Leipzig,Mannheim and Munich).  Country: Germany | It is a MultiCare cohort study of 3,189 multimorbid patients. Data were collected in interviews with general practitioners and in-depth patient interviews. |
| The modeling of internalizing disorders on the basis of patterns of lifetime comorbidity: associations with psychosocial functioning and psychiatric disorders among first-degree relatives [115] | Confirmatory factor analysis | Internalizing disorders.  Mood disorders.  Anxiety disorders.  Distress disorders.  Fear disorders. | Employment, household income, marital status, relationship quality, social adjustment. | Participants between ages 14 and 33 were initially recruited from nine randomly selected high schools within western Oregon.  Country: USA | An age-based cohort was followed longitudinally. Four diagnostic evaluations were conducted in an age-based cohort of 816 people over a 15-year interval from 1993 to 2007. |
| The patterns of Non-communicable disease Multimorbidity in Iran: A Multilevel Analysis [74] | Latent Class Analysis | **Males**  Class 1: Diabetes.  Class 2: Asthma and wheezing.  Class 3: Musculoskeletal diseases.  **Females**  Class 1: Asthma and wheezing.  Class 2: Pre-skeletal diseases.  Class 3: Musculoskeletal diseases. | Sex, age, physical activity, BMI, daily tobacco use, fruit and vegetables intake, education level, job and province level. | People aged 20–70 years, from 31 provinces (7033 from urban and 3036 from rural areas) in Iran.  Country: Iran | Data was inquired from the nationwide survey performed in 2011, according to the WHO stepwise approach on NCD risk factor with 10069 participants. |
| Trends of multimorbidity in 15 European countries: a population-based study in community-dwelling adults aged 50 and over [135] | Expert knowledge | Multimorbidity.  Cardiometabolic.  Musculoskeletal.  Respiratory.  Neurodegenerative.  Cancer. | Sex and country. | Multimorbid patients aged 50 or older from 15 Countries: Austria, Belgium, Czech Republic, Denmark, Estonia, France, Germany, Netherlands, Italy, Poland, Portugal, Spain, Sweden, Slovenia and Switzerland. | An ecological study with temporal series population based analysis was realised using data from the Survey of Health, Ageing and Retirement in Europe (SHARE) project. The sample consisted of 274,614 individuals. |
| Use of latent class analysis to identify multimorbidity patterns and associated factors in Korean adults aged 50 years and older [75] | Latent class analysis | Relatively healthy.  Cardiometabolic conditions.  Arthritis, asthma, allergy, depression, thyroid. | Age, gender, household income, education, occupation. | Adults 50 years of age and older who participated in the sixth Korean National Health and Nutrition Examination Survey.  Country: Korea | A cross-sectional study, Korea National Health and Nutrition Examination Survey (2013-2015) and its sample was 8,370 patients. |
| Using item response theory with health system data to identify latent groups of patients with multiple health conditions [76] | Latent class analysis | Complex Mental Health.  Complex Diabetes.  Liver Disease.  Cancer+Cardiac.  Cancer+Mental Health. | Sex, age, marital status, occupation, race, medical visits and non-face encounter, impatient admission. | Adult patients receiving care from the Veteran’s Health Administration (VHA) in 2014 that were at high-risk for hospitalization.  Country: USA | This was a retrospective cohort study using 68,400 high-risk Veteran’s Health Administration (VHA) patients. |
| Latent classes of posttraumatic psychiatric comorbidity in the general population [138] | Latent class analysis | Substance use with high comorbidity  Depression with high comorbidity  Broad high comorbidity | Median age, sex. Income, marital status | 1.4 million individuals who had one or more traumatic events recorded in Danish national healthcare and social registries.  Country: Denmark | Data were obtained from a cohort of approximately 1.4 million individuals who had one or more traumatic events recorded in Danish national healthcare and social registries between 1994 and 2016 |
