# Supplementary material for: Social determinants of multimorbidity patterns: A systematic review
Source: Front Public Health. 2023 Mar 27;11:1081518. doi: 10.3389/fpubh.2023.1081518 (PMC10084932; doi:10.3389/fpubh.2023.1081518)
Supplement: Supplementary file 5 [file Table_5.DOCX]

Supplementary Material

# Table 5: Social determinants according to the pattern extraction method

| Social determinants | Latent class analysis | Cluster techniques | Factor analysis | Machine learning | Expert selection |
| --- | --- | --- | --- | --- | --- |
| Sociodemographic characteristics | [41]  [42]  [43]  [44]  [45]  [46]  [47]  [48]  [49]  [50]  [51]  [52]  [53]  [54]  [55]  [57]  [58]  [59]  [60]  [61]  [62]  [63]  [64]  [65]  [66]  [68]  [69]  [70]  [71]  [72]  [73]  [74]  [75]  [76]  [77]  [138] | [78]  [79]  [80]  [81]  [82]  [83]  [84]  [85]  [86]  [87]  [88]  [89]  [90]  [91]  [92]  [93]  [94]  [95]  [96]  [97]  [98]  [99] | [100]  [101]  [103]  [104]  [108]  [109]  [110]  [111]  [112]  [113]  [114]  [115]  [116]  [117]  [118] | [119]  [120]  [121]  [123]  [124]  [125]  [126]  [127]  [128] | [129]  [130]  [131]  [132]  [133]  [134]  [135]  [136] |
| Socioeconomic status | [41]  [42]  [43]  [44]  [45]  [46]  [47]  [48]  [49]  [50]  [51]  [52]  [53]  [54]  [55]  [57]  [58]  [59]  [61]  [62]  [63]  [64]  [65]  [69]  [71]  [73]  [74]  [75]  [76]  [77]  [138] | [78]  [85]  [87]  [88]  [89]  [95]  [96]  [98] | [100]  [102]  [103]  [105]  [106]  [107]  [108] | [122]  [127] | [131]  [132] |
| Lifestyle | [42]  [43]  [48]  [52]  [53]  [57]  [58]  [59]  [60]  [61]  [65]  [66]  [67]  [67]  [71]  [72]  [73]  [74]  [77] | [78]  [80]  [85]  [87]  [88]  [96] | [107] |  | [132]  [134] |
| Social networks | [46]  [61] | [96] | [115] |  | [132] |
| Living area characteristics | [49]  [51]  [53]  [69]  [70]  [73]  [74] | [82]  [88]  [97] | [100]  [103]  [105] |  | [132]  [135] |
| Health service use | [43]  [44]  [50]  [55]  [56]  [59]  [61]  [73]  [76] | [78]  [84] | [103] | [119]  [120] |  |
